# Compact zinc finger base editors that edit mitochondrial or nuclear DNA in vitro and in vivo

**DOI:** 10.1038/s41467-022-34784-7

**Published:** 2022-11-23

**Authors:** Julian C. W. Willis, Pedro Silva-Pinheiro, Lily Widdup, Michal Minczuk, David R. Liu

**Affiliations:** 1 Merkin Institute of Transformative Technologies in Healthcare, Broad Institute of MIT and Harvard, Cambridge, MA USA; 2 Department of Chemistry and Chemical Biology, Harvard University, Cambridge, MA USA; 3 Howard Hughes Medical Institute, Harvard University, Cambridge, MA USA; 4 MRC Mitochondrial Biology Unit, University of Cambridge, Cambridge, UK

**Keywords:** Targeted gene repair, Genetic engineering

## Abstract

DddA-derived cytosine base editors (DdCBEs) use programmable DNA-binding TALE repeat arrays, rather than CRISPR proteins, a split double-stranded DNA cytidine deaminase (DddA), and a uracil glycosylase inhibitor to mediate C•G-to-T•A editing in nuclear and organelle DNA. Here we report the development of zinc finger DdCBEs (ZF-DdCBEs) and the improvement of their editing performance through engineering their architectures, defining improved ZF scaffolds, and installing DddA activity-enhancing mutations. We engineer variants with improved DNA specificity by integrating four strategies to reduce off-target editing. We use optimized ZF-DdCBEs to install or correct disease-associated mutations in mitochondria and in the nucleus. Leveraging their small size, we use a single AAV9 to deliver into heart, liver, and skeletal muscle in post-natal mice ZF-DdCBEs that efficiently install disease-associated mutations. While off-target editing of ZF-DdCBEs is likely too high for therapeutic applications, these findings demonstrate a compact, all-protein base editing research tool for precise editing of organelle or nuclear DNA without double-strand DNA breaks.

## Abstract (web summary)

Zinc finger (ZF) arrays are programmable DNA-binding proteins. Here the authors report ZF-DddA-derived cytosine base editors (DdCBEs) and optimise their architectures to improve targeting; they apply these variants in vitro and in vivo to mitochondrial base editing and show higher editing than ZF deaminases.

## Introduction

Mitochondria are essential organelles in almost all eukaryotic cells. Each cell contains hundreds of circular copies of mtDNA encoding a set of proteins, rRNAs, and tRNAs that facilitate mitochondrial ATP production^1–4^. Mutations in the mitochondrial genome can give rise to mitochondrial genetic diseases such as mitochondrial encephalopathy, lactic acidosis and stroke-like episodes (MELAS), and Leber hereditary optic neuropathy (LHON), among many others^5–8^. The ability to install precise sequence changes within mtDNA could be invaluable to study and potentially treat mitochondrial genetic diseases, which collectively afflict approximately one in 5000 people^9^.

ZF- and TALE-nucleases have been successfully used in mitochondria to selectively target mutation-containing mtDNA for cleavage and reduce heteroplasmy^10,11^. However, because double-strand break repair pathways are lacking in mitochondria^12,13^, such targeted-nuclease strategies result in target DNA loss, rather than in targeted sequence changes.

Base editors use programmable DNA-binding proteins together with a natural or laboratory-evolved DNA deaminase to mediate precise targeted sequence changes in DNA within human cells^14,15^. Because no system for the efficient import of nucleic acids into mitochondria has been identified thus far, CRISPR base editors, which require a guide RNA component, currently cannot be used effectively in mitochondria^16,17^.

In contrast, protein import into mitochondria is well-characterized^18^, raising the possibility that all-protein, CRISPR-free base editors might enable the precision editing of organellar as well as nuclear genomes. The discovery of the first dsDNA-specific cytidine deaminase (DddA) enabled our recent development of efficient CRISPR-free base editors that edit nuclear and organelle DNA^19^. The first all-protein base editors, DdCBEs, use programmable DNA-binding TALE repeat array proteins together with a split DddA and a uracil glycosylase inhibitor (UGI) to mediate targeted C•G-to-T•A editing in nuclear, mitochondrial, and chloroplast DNA^19–21^. Full-length DddA can be split at position G1397 into two catalytically inactive halves, a 108-residue N-terminal fragment (DddA^N^) and a 30-amino acid C-terminal fragment (DddA^C^). The binding of two TALE–split-DddA–UGI fusions to adjacent sites promotes the reassembly of functional DddA for deamination of target cytosines within the dsDNA spacing region between the adjacent target sites.

Due primarily to the large size of TALE repeat arrays, DdCBEs are too large to package in a single AAV construct for in vivo delivery, complicating their application in animals and as potential therapeutics (Fig. S1). TALE arrays can also be challenging to construct due to their repetitive sequence^22,23^, have certain target sequence requirements^24^, and add a large number of immunogenic epitopes when fused to a protein. Here we describe the development of all-protein zinc finger DdCBEs (ZF-DdCBEs) that can edit mitochondrial or nuclear DNA in vitro and in vivo. ZFs offer compact DNA recognition; each 28-residue ZF repeat recognizes three target nucleotides, while each 34-residue TALE repeat recognizes only a single nucleotide. In addition to being natively less repetitive in sequence and thus easier to construct, ZFs represent the most abundant class of proteins in the human proteome and are thought to be less immunogenic than most foreign proteins^25,26^. The development of ZF-DdCBEs thus offers more compact base editors with different targeting properties and potentially lower immunogenicity than TALE-based DdCBEs.

Church and coworkers previously reported efforts to develop ZF-targeted deaminases using a ZF array fused to activation-induced cytidine deaminase (AID)^27^. These efforts led to very low editing efficiencies in human cells because ZF arrays bind dsDNA but all cytidine deaminases reported until 2020 require a ssDNA substrate^28^. Independently, Kim and coworkers recently reported ZF deaminases (ZFDs) composed of a ZF array fused to split DddA and UGI^29^. ZFDs support base editing of mitochondrial or nuclear DNA in vitro, but their optimization was primarily limited to domain ordering and varying the length of the amino acid linkers connecting the ZF arrays and DddA halves. To develop efficient ZF-DdCBEs, including for in vivo applications, we built on our previous development of DdCBEs and comprehensively engineered their architecture, ZF scaffolds, and DddA deaminase components. Our v7 architecture supports a 10-fold average improvement in mitochondrial base editing efficiency over an initial v1 architecture that simply replaced TALE repeat arrays in DdCBE with ZF arrays, and a >3.6-fold average improvement over ZFDs in side-by-side comparisons. We identified four strategies to reduce off-target editing caused by spontaneous split DddA reassembly and integrated these approaches to engineer high-specificity ZF-DdCBE variants with lowered off-target editing and efficient on-target editing of mitochondrial or nuclear DNA. Their compact size enables ZF-DdCBEs to be delivered with a single AAV in vivo in mice, resulting in efficient mitochondrial base editing in the heart, liver, and skeletal muscle. Alongside high levels of on-target editing, we also observed high levels of off-target editing which likely preclude therapeutic applications. Nonetheless, ZF-DdCBEs enable compact, all-protein in vitro and in vivo base editing for the precise editing of nuclear or organelle DNA without double-strand DNA breaks suitable for research uses.

## Results

### Architecture engineering to optimize ZF-DdCBE on-target activity

**Fig. 1:**
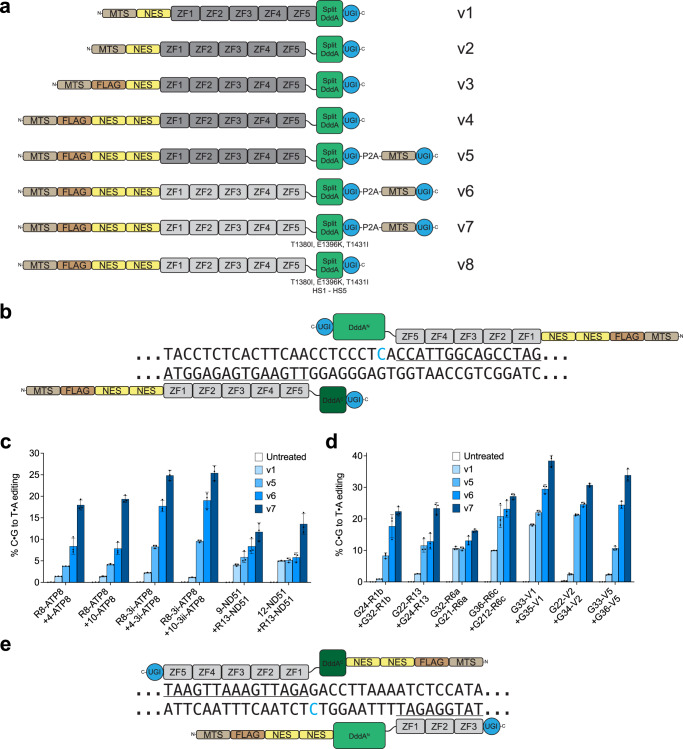
Optimizing ZF-DdCBEs increases base editing efficiency in mitochondria. **a** Architectures of optimized ZF-DdCBEs showing progression from v1 to v8. The components are a mitochondrial targeting signal, FLAG tag, nuclear export signal(s), ZF array with either canonical ZF scaffold (dark grey) or optimized ZF scaffold (light grey), Gly/Ser-rich flexible linker, split DddA deaminase (with or without activity-enhancing mutations and specificity-enhancing mutations) and UGI. **b** A v8 ZF-DdCBE pair with canonical C-terminal architecture. The ZF-DdCBE pair shown is 9-ND51+R13-ND51. **c**, **d** Mitochondrial DNA base editing efficiencies of HEK293T cells treated with (**c**) six optimized ZF-DdCBE pairs used to establish architectural improvements or (**d**) seven additional optimized ZF-DdCBE pairs. **e** A v8 ZF-DdCBE pair with N-terminal architecture. The ZF-DdCBE pair shown is LT51-Mt-tk+RB38-Mt-tk. For (**b**, **e**) ZF binding sites are underlined and the cytosine with the highest editing efficiency is colored in blue. For (**c**–**d**) values and errors reflect the mean ± s.d. of *n* = 3 independent biological replicates. The editing efficiencies shown are for the most efficiently edited C•G within the spacing region. Source data are provided as a Source Data file.

Our initial ZF-DdCBE architecture (designated v1) was based on TALE-targeted DdCBEs^19^ and consisted of a five-ZF (5ZF) array preceded by a mitochondrial targeting signal (MTS) from the human *ATP5F1B* gene and a nuclear export signal (NES) from MVM NS2 as previously reported for mitochondrially targeted ZF nucleases (mtZFNs)^10,30^, followed by a two-amino acid linker, one split DddA half, and one UGI (Fig. 1a). To target sites in human mtDNA, we used a previously characterized 5ZF array from the literature to form one half of a ZF-DdCBE pair^10^, and we designed two 5ZF arrays following the modular assembly approach^31,32^ that each formed the other half of a ZF-DdCBE pair. Using a total of six 5ZF arrays, this resulted in two ZF-DdCBE pairs targeting the mitochondrial *ATP8* gene and two ZF-DdCBE pairs targeting the mitochondrial *ND5* gene with 4-, 10-, 9-, and 12-bp spacing regions containing TC dinucleotides, respectively (Fig. S2a). ZF-DdCBE pairs throughout this study are named A+B where A and B specify the left and right ZF, respectively. We refer to this ZF-DdCBE architecture as C-terminal, in which split DddA is fused C-terminally to the ZF array (Fig. 1b). While iterated ZF selection approaches are considered to yield ZF arrays with higher target binding activity and specificity^33,34^, we chose the simpler modular assembly approach to determine if a highly accessible ZF design strategy readily available to most researchers could support ZF-DdCBEs.

When expressed in human HEK293T cells following plasmid transfection, this v1 ZF-DdCBE architecture resulted in base editing efficiencies ranging from 1–2% for four ZF-DdCBE pairs tested across two sites (Fig. S2b). These results establish that ZF-DdCBEs can be constructed using ZF arrays in place of TALE repeats and can successfully install targeted C-to-T edits in mitochondria in living cells, albeit with very low initial activity. We used these v1 ZF-DdCBEs as the starting point for development and optimization.

We hypothesized that ZF-DdCBE editing outcomes might be limited if the linker between the ZF array and the split DddA deaminase constrained access of reassembled DddA to the target nucleotide(s). We replaced the two-amino acid linker in architecture v1 with a 7- or 13-amino acid Gly/Ser-rich flexible linker, or a 32-amino acid XTEN linker. Across the four ZF-DdCBE pairs tested, using a 13-amino acid Gly/Ser-rich flexible linker supported the greatest improvements in editing efficiency, on average increasing editing efficiency 1.7-fold over v1 ZF-DdCBEs (Fig. S2b). We designated this architecture v2 (Fig. 1a).

We hypothesized that suboptimal cellular localization of ZF-DdCBEs might be impairing editing outcomes if they are transported into mitochondria inefficiently or remain partially localized in the nucleus. Since the mitochondrial import efficiency of a protein depends on its local structure adjacent to the MTS^35^, in an effort to improve ZF-DdCBE mitochondrial import we introduced an unstructured epitope (a FLAG or HA tag) immediately downstream of the MTS as previously reported for mtZFNs^10^. Across the four ZF-DdCBE pairs tested, inserting a FLAG tag led to an average improvement in editing efficiency over v2 of 1.5-fold (Fig. S2c). We designated this architecture v3 (Fig. 1a).

To minimize the fraction of ZF-DdCBE that was localized to the nucleus in order to maximize organelle editing efficiency, we tested the effect of adding an additional NES from HIV-1 Rev, MAPKK, or MVM NS2 to v3 ZF-DdCBEs, either downstream of the existing internal NES or at the C-terminus of the protein. Across the four ZF-DdCBE pairs tested, inserting an additional internal NES from MAPKK led to an average improvement in editing efficiency of 1.4-fold over v3 (Fig. S2d). We designated this architecture v4 (Fig. 1a).

Next we investigated whether incomplete inhibition of mitochondrial base excision repair could be limiting ZF-DdCBE editing efficiency. To test if different UGI positioning or copy number could enhance mitochondrial base editing efficiency, we moved the location of UGI within the fusion protein to a position N-terminal of the 5ZF array, appended a second copy of UGI to the C-terminus, or expressed a separate mitochondrially targeted UGI in trans using a ribosomal skipping P2A peptide (with or without removing the C-terminally fused UGI). Across the four ZF-DdCBE pairs tested, expressing an additional copy of MTS-UGI in trans led to an average improvement in editing efficiency over v3 of 1.3-fold (Fig. S2e). Combining this improvement with the v4 architecture to create v5 resulted in editing efficiency on average 3.4-fold over that of v1 ZF-DdCBEs across the four ZF-DdCBE pairs tested (Fig. 1a, c). Collectively, these data show that ZF-DdCBE editing efficiency can be substantially improved compared to the initial v1 architecture. These optimizations include increasing the linker length between the ZF array and split DddA, improving mitochondrial import, enhancing nuclear export, and further suppressing residual cellular UDG activity.

### Effects of ZF array length and composition on ZF-DdCBE performance

Next, we turned our attention to optimizing ZF arrays for ZF-DdCBEs. Natural ZF arrays are found in transcription factors that localize to the nucleus, and contain cryptic nuclear localization signals (NLSs) present within the ZF fold^36,37^. We hypothesized that cycling of nuclear import and export mediated by competition between NLS and NES motifs may impede localization of ZF-DdCBEs to the mitochondria and therefore limit mitochondrial base editing. We reasoned that shorter ZF arrays with fewer NLS-containing ZF repeats would exhibit weaker nuclear localization, and therefore may support higher mitochondrial editing efficiency due to improved mitochondrial localization.

To understand the effects of ZF array length on ZF-DdCBE editing efficiency, first we truncated each 5ZF to create a set of two 4ZFs and a set of three 3ZFs by removing either one or two individual ZFs, respectively (Fig. S3a). We tested the resulting four 4ZF+4ZF combinations and nine 3ZF+3ZF combinations in the context of ZF-DdCBEs derived from each of our original four ZF-DdCBE pairs (Fig. S3b–i). For each of the ZF-DdCBE pairs we saw that ZF truncation affected both the editing efficiency and the position of the target nucleotide(s) that are edited within the spacing region. While in general we observed that ZF-DdCBEs containing shorter ZFs exhibited lower editing efficiency, we identified six 3ZF+3ZF combinations with substantially higher editing efficiencies than their parent 5ZF+5ZF pairs despite using shorter ZF arrays. These data suggest that ZF arrays as short as 3ZF are sufficient to mediate efficient mitochondrial C•G-to-T•A base editing, and that the precise location of the ZF binding site, and therefore deaminase positioning, strongly influences which target bases are edited most efficiently.

To investigate the effects of ZF array length more systematically, we identified five sites within human mtDNA that comprise a TC-containing spacing region flanked by sequences consisting exclusively of (GNN)_n_ trinucleotides. We selected (GNN)_n_-rich sites because ZFs containing GNN-binding modules are predicted to have a higher binding affinity, on average, than ANN, TNN, or CNN-binding modules^38^ and therefore testing ZFs containing exclusively GNN-binding modules may minimize variability in binding affinity when designing ZF arrays by modular assembly. At each site we designed a panel of 3ZFs that could be extended outwards away from the spacing region to create longer 4ZF or 5ZF arrays which all shared the same split DddA positioning and therefore maintained a fixed spacing region, enabling a direct comparison (Fig. S4). We tested 16 ZF-DdCBEs containing 3ZF+3ZF pairs and compared their performance against 16 4ZF+4ZF and 16 5ZF+5ZF pairs (Fig. S5). The results indicated that on average longer ZF arrays correlated with increased editing efficiency, with 4ZF+4ZF pairs and 5ZF+5ZF pairs leading to an average 2.2- and 2.9-fold improvement relative to 3ZF+3ZF pairs, respectively.

We also investigated the effects of including an extended linker in 4ZF and 5ZF arrays, which has been reported to reduce DNA-binding strain in longer ZF arrays^39–42^. We compared the editing efficiency achieved by 16 4ZF+4ZF and 16 5ZF+5ZF ZF-DdCBE pairs against their counterparts in which an extended linker was incorporated into each ZF array (Fig. S5). We found that 4ZF and 5ZF arrays designed using exclusively canonical linkers supported higher editing efficiencies on average, and therefore we did not use extended linkers in subsequent designs.

### Defining alternative ZF scaffolds improves ZF-DdCBE performance

Next we explored alternative ZF scaffolds that might improve ZF-DdCBE editing efficiency by enhancing DNA-binding affinity or reducing the strength of the inherent cryptic NLS sequences that form part of the ZF fold. Each ZF repeat within a ZF array is linked together by short flexible linkers and consists of a beta-sheet motif, seven variable DNA-binding residues, and an alpha-helical motif. In this study, we define a ZF scaffold to consist of a beta-motif, an alpha-motif, and a flexible linker motif, independent of the DNA-binding residues that specify the targeted trinucleotide DNA sequence. The sequences of the beta-motif, alpha-motif, and flexible linker motif vary between individual ZF repeats within both natural and designed ZF arrays (Fig. S6a–d). We hypothesized that ZF-DdCBE editing efficiency could be improved by eliminating this sequence variation to create ZF arrays composed of identical repeating scaffolds exclusively containing motif sequences with superior performance. We defined a set of eight new ZF scaffolds, named X1-X8, and used these to create ZF arrays in which every ZF repeat shared an identical scaffold sequence. These eight scaffold sequences represent all possible combinations of the two beta-motifs, two alpha-motifs, and two linker motifs found in canonical *ZNF268*-derived ZFs^43^ (Fig. S6e). Across six ZF-DdCBE pairs of length varying from 3ZF to 5ZF tested at two target sites, we found that scaffold X1 conferred an average of 1.7-fold improvement relative to the canonical *ZNF268*-derived scaffold (Fig. S6f–k). These observations demonstrated that ZF scaffold engineering can create ZF-DdCBEs with higher editing efficiency across different sites and different ZF array lengths.

To explore whether other ZF scaffold sequences can confer even higher base editing activity to ZF-DdCBEs than canonical *ZNF268*-derived sequences, we searched natural ZF diversity for additional ZF scaffolds. We searched the human proteome for ZF-containing sequences and identified 3356 unique beta-motifs, 625 unique alpha-motifs, and 549 unique linker motifs. We calculated amino acid frequencies at each position within the motifs and used these to define 96 consensus beta-motifs, 18 consensus alpha-motifs, and 24 consensus linker motifs based on the most common amino acids at each position (Fig. S7). We constructed ZF-DdCBE variants based on the X1 scaffold in which we replaced for every ZF within the 5ZF array either the beta-motif only, alpha-motif only, or the linker motif only with one of the new consensus motifs. Testing these ZF-DdCBE pairs revealed a new beta-motif that conferred a 1.3-fold increase in editing over the X1 scaffold (Fig. S8a–d, g) and a new alpha-motif that conferred a 1.2-fold increase over the X1 scaffold (Fig. S8e, h). We found no new linker motifs that outperformed the X1 scaffold (Fig. S8f, i).

By combining the best-performing beta-motif and alpha-motif, we defined a new ZF scaffold V20 and variant V2. We also defined a new ZF scaffold AGKS derived from the human transcription factor Sp1C that showed increased editing efficiency over X1 (Fig. S9, Supplementary Note 1). We tested this set of four new ZF scaffolds (X1, V2, V20, and AGKS) using six ZF-DdCBE pairs at two sites (Fig. S10). For each ZF-DdCBE pair tested, editing efficiency was improved compared to the canonical *ZNF268*-derived scaffold for all four new ZF scaffold variants. Selecting the best-performing ZF scaffold for each pair led to an average 2.2-fold improvement over the canonical *ZNF268*-derived scaffold. We combined this change with v5 architecture to create v6 (Fig. 1a). Across the six ZF-DdCBE pairs tested, v6 on average increased base editing efficiency 6.6-fold over v1 and 2.0-fold over v5 (Fig. 1c). These results collectively establish that ZF-DdCBE base editing efficiency can be enhanced by optimizing the design of ZF arrays used for DNA targeting.

### Introducing DddA mutations enhances ZF-DdCBE base editing efficiency

As a final strategy to optimize the architecture and sequence of ZF-DdCBEs for on-target editing efficiency, we tested mutations in DddA for their ability to enhance ZF-DdCBE editing. We recently used phage-assisted continuous evolution (PACE) to evolve DddA deaminase variants that support improved TALE-based DdCBE activity^20^. To test if evolved DddA mutations improve ZF-DdCBEs, we assayed combinations of Q1310R, T1314A, S1330I, T1380I, and E1396K in DddA^N^ with and without T1413I in DddA^C^ (Fig. S11). Across the four ZF-DdCBE pairs tested, the triple mutant T1380I, E1396K, T1413I led to an average improvement in editing over canonical DddA of 1.6-fold. We combined these mutations with v6 architecture to create v7 (Fig. 1a). These results suggest that using a more active DddA variant can improve ZF-DdCBE editing outcomes.

To validate our ZF-DdCBE optimizations, we re-tested our v1, v5, v6, and v7 architectures at our original set of six ZF-DdCBE pairs at two sites. Across these six pairs, v7 ZF-DdCBEs achieved an average of 11-fold higher editing over v1 (Fig. 1c). To demonstrate that these architectural improvements are generalizable to ZF-DdCBEs targeting any sites across mtDNA, we tested seven new ZF-DdCBE pairs targeting seven different sites across four genes, and compared v1, v5, v6 and v7 architectures (Figs. 1d, S12). Across these seven pairs, v7 ZF-DdCBEs achieved an average of 9.5-fold higher editing relative to v1.

We note that for six of these seven pairs, one half of the ZF-DdCBE pair uses an N-terminal ZF-DdCBE architecture in which split DddA is fused N-terminally to the ZF array, while the other half of the ZF-DdCBE pair uses a canonical C-terminal fusion of split DddA. Importantly, N-terminal fusions of split DddA with TALE repeat arrays do not result in efficient DdCBEs, thus requiring that TALE-DdCBE halves must target opposite DNA strands. In contrast, the compatibility of ZF-DdCBEs with N-terminal or C-terminal split DddA fusions provides researchers with the flexibility to design ZF-DdCBE pairs that bind either the same or opposite DNA strands around the target nucleotide(s). Thus, ZF-DdCBE pairs can be designed in four different combinations: C-terminal+C-terminal (Fig. 1b), C-terminal+N-terminal, N-terminal+C-terminal, or N-terminal+N-terminal (Fig. 1e), providing additional targeting options not available to TALE-DdCBEs. Collectively, our findings integrate optimized architectures, improved ZF scaffolds, DddA activity-enhancing mutations, and split DddA fusion orientation flexibility to greatly enhance the editing efficiency of compact all-protein base editors.

**Fig. 2:**
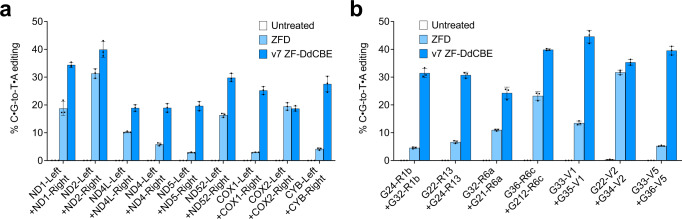
Optimized ZF-DdCBEs display increased base editing efficiency over ZFDs in mitochondria. **a**, **b** Comparison of mitochondrial DNA base editing efficiencies of HEK293T cells treated with either ZFD or optimized ZF-DdCBE pairs at genomic target sites chosen by (**a**) Lim et al.^29^, or (**b**) by this study. For (**a**, **b**) values and errors reflect the mean ± s.d. of *n* = 3 independent biological replicates. The editing efficiencies shown are for the most efficiently edited C•G within the spacing region. Source data are provided as a Source Data file.

To directly compare the performance between ZFDs recently reported by Kim and coworkers^29^ with that of optimized ZF-DdCBEs, we converted nine mtDNA-targeting ZFD pairs into the v7 ZF-DdCBE architecture and tested X1, AGKS and V20 ZF scaffolds. Across the nine sites tested, the best-performing ZF scaffold for each pair led to an average 3.6-fold improvement in on-target editing efficiency for ZF-DdCBEs compared to ZFDs (Fig. 2a, S13). In addition, we converted a separate set of seven optimized v7 ZF-DdCBEs into ZFD architectures and tested their relative performance at editing mitochondrial sites. The optimized ZF-DdCBEs led to an average 3.9-fold higher on-target editing efficiency compared to ZFDs across the seven pairs tested (Fig. 2b). Collectively, these side-by-side comparison data at 16 distinct mtDNA target sites suggest that the more extensively optimized ZF-DdCBEs offer substantially higher on-target editing efficiencies than ZFDs.

### Characterizing off-target editing by ZF-DdCBEs

**Fig. 3:**
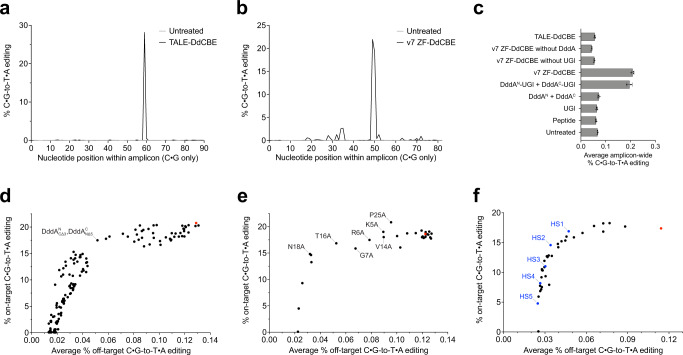
High-specificity ZF-DdCBE variants reduce mitochondrial off-target editing. **a** Mitochondrial DNA base editing efficiencies within amplicon ND4 of HEK293T cells treated with ND4-DdCBE. **b** Mitochondrial DNA base editing efficiencies within amplicon ATP8 of HEK293T cells treated with v7 ZF-DdCBE pair R8-3i-ATP8+4-3i-ATP8. **c** Off-target editing efficiencies within mitochondrial off-target amplicon ND5.1 of HEK293T cells treated with ND4-DdCBE, v7 ZF-DdCBE pair R8-3i-ATP8+4-3i-ATP8, or individual components of the v7 ZF-DdCBE architecture. **d**–**f** On-target and average off-target editing efficiencies within amplicon ATP8 of HEK293T cells treated with canonical v7 ZF-DdCBE pair R8-3i-ATP8+4-3i-ATP8 (colored in red) or variants containing (**d**) DddA^N^ and DddA^C^ truncations, (**e**) Ala point mutations within DddA^C^, or (**f**) combinations of DddA^N^ and DddA^C^ truncations, point mutations within DddA^C^, and/or fused catalytically inactivated DddA^N^. High-specificity variants HS1 to HS5 are colored in blue. For (**a**, **b**) and (**d**–**f**) values reflect the mean of *n* = 3 independent biological replicates. For (**c**), values and errors reflect the mean ± s.d. of *n* = 3 independent biological replicates. For (**d**–**f**) the editing efficiencies shown are for the most efficiently edited C•G within the spacing region. Source data are provided as a Source Data file.

We compared amplicon-wide (~200 bp) sequencing data for a high-performing TALE-based DdCBE pair^19^ and a v7 ZF-DdCBE pair, both targeting sites in mtDNA. We observed efficient on-target editing (28%) and very low frequencies of off-target editing for the TALE-based DdCBE pair (typically ≤0.2% C•G-to-T•A conversion at each off-target nucleotide in the amplicon), but much higher off-target editing of up to 2% at C•G base pairs scattered across the amplicon for the v7 the ZF-DdCBE pair (Fig. 3a, b). These results suggest that ZF-DdCBEs introduce a higher level of off-target edits than TALE-based DdCBEs, which if not minimized may limit their therapeutic applicability.

To test the effect of dose titration on on-target and off-target editing using TALE-based DdCBE and v7 ZF-DdCBE pairs, we incrementally lowered the amount of plasmid transfected with or without changing the duration of treatment (Fig. S14a, b). We observed that on-target editing efficiency for both editors increased from 24 to 72 h post-transfection concomitant with rises in off-target editing. Similarly, we found that higher doses of the ZF-DdCBE pair lead to increased on-target editing efficiency in addition to higher off-target editing (Fig. S14c). These data suggest that reducing the dose of plasmid DNA delivered does not provide a simple approach to eliminate off-target editing, without compromising on-target editing efficiency.

To investigate if the higher level of off-target editing activity exhibited by ZF-DdCBEs arises from spontaneous DddA reassembly, from ZF-dependent DddA reassembly, or both, we delivered individual components of the v7 ZF-DdCBE architecture into mitochondria (Fig. 3c). We used targeted amplicon sequencing to initially assess mtDNA-wide off-target editing activity. Transfected HEK293T cells expressing an inactive mitochondrially targeted short peptide as a negative control did not exhibit any detectable editing compared to untreated cells. Cells expressing mitochondrially targeted UGI also did not display any editing above background (Fig. 3c), demonstrating that the endogenous mutational load arising from spontaneous deamination is very low.

Cells expressing mitochondrially localized DddA^N^-UGI and DddA^C^-UGI displayed non-targeted editing, while cells expressing mitochondrially localized DddA^N^ and DddA^C^ did not (Fig. 3c). These results suggest that the spontaneous reassembly of split DddA halves is sufficient to give rise to untargeted deaminase activity, recapitulating the native-like activity of the full-length DddA toxin. While the natural base-excision repair (BER) pathway endogenous to mitochondria can adequately repair C-to-U deamination caused by DddA reassembly, when mitochondrial uracil BER is suppressed by UGI, C•G-to-T•A conversions are observed.

Delivering a representative v7 ZF-DdCBE slightly increased off-target editing compared to expression of DddA^N^-UGI and DddA^C^-UGI without ZFs, indicating a ZF-dependent component of off-target editing (Fig. 3c). We observed that removal of either UGI or the split-DddA from the ZF-DdCBE architecture abolished detectable off-target editing. Collectively, these results indicate that ZF-DdCBE off-target editing arises from spontaneous association of the DddA split halves under conditions of suppressed uracil BER by UGI, and that the inclusion of a ZF array can increase off-target editing.

ZF-DdCBE off-target editing could thus proceed via three different paths: (i) dual ZF-dependent off-target editing in which both ZF-DdCBE halves bind to off-target DNA sequences in close spatial proximity; (ii) single ZF-dependent off-target editing in which a single ZF-DdCBE protein binds to off-target DNA sequences and transiently recruits the other DddA half; or (iii) ZF-independent off-target editing in which the two DddA split halves spontaneously reassemble without requiring ZF binding. We hypothesized that weakening the interaction between the DddA split halves could reduce single ZF-dependent and ZF-independent off-target editing, without necessarily impairing on-target editing efficiency.

We previously reported that delivery into mitochondria of DddA^N^-UGI and DddA^C^-UGI preceded by 3xHA tag and 3xFLAG tag sequences, respectively, gave rise to no detectable C•G-to-T•A conversion above background^19^. In contrast, the delivery of both DddA^N^-UGI and DddA^C^-UGI each preceded by a Gly/Ser-rich flexible linker produced measurable C-to-T editing in mtDNA (Fig. 3c). To test whether the amino acid sequences immediately upstream of DddA^N^ and DddA^C^ could be modulated to change the level of editing activity observed, we systematically replaced the preceding Gly/Ser-rich flexible linker with sequences containing increasing numbers of negatively charged HA or FLAG tag motifs. We observed that the non-targeted editing activity decreased as the total negative charge density increased (Fig. S15). These results suggest that destabilization of the interaction between the split DddA halves can reduce off-target editing caused by spontaneous reassembly of DddA.

### Engineering high-specificity ZF-DdCBEs

These findings suggested several strategies to reduce ZF-DdCBE off-target editing by reducing the binding affinity between the split DddA halves. First, truncation of DddA^N^ and DddA^C^ or shifting the position of the split site within DddA may weaken the ability of the DddA halves to spontaneously reassemble in the absence of target DNA co-binding. Second, introducing point mutations into DddA^C^ might destabilize the binding affinity between the DddA halves and reduce their spontaneous association. Third, increasing electrostatic repulsion between DddA^N^ and DddA^C^ by introducing negatively charged residues upstream or downstream of DddA^N^ and DddA^C^ may also impede target-independent reassembly. Fourth, fusion of a catalytically inactivated DddA^N^ might outcompete spontaneous reassembly of DddA^N^ with DddA^C^ in the absence of target-templated co-localization. We tested each of these strategies using a 3ZF+3ZF v7 ZF-DdCBE pair (R8-3i-ATP8+4-3i-ATP8) targeting the mitochondrial *ATP8* gene in HEK293T cells and conducted high-throughput amplicon sequencing to detect on-target and off-target editing.

### DddA truncation to enhance ZF-DdCBE specificity

First, we explored the effects of DddA^N^ and DddA^C^ truncation on ZF-DdCBE performance. We created a series of ZF-DdCBE constructs in which DddA^N^ was incrementally C-terminally truncated by 1 to 6 residues, which we named ${{{{{{\rm{DddA}}}}}}}_{{{{{{\rm{C}}}}}}\triangle 1}^{{{{{{\rm{N}}}}}}}$ to ${{{{{{\rm{DddA}}}}}}}_{{{{{{\rm{C}}}}}}\triangle 6}^{{{{{{\rm{N}}}}}}}$. We also created a series of ZF-DdCBE constructs in which DddA^C^ was either incrementally truncated at its N-terminus by 1 to 15 residues, designated ${{{{{{\rm{DddA}}}}}}}_{{{{{{\rm{N}}}}}}\triangle 1-15}^{{{{{{\rm{C}}}}}}}$, or incrementally truncated at its C-terminus by 1 to 9 residues, designated ${{{{{{\rm{DddA}}}}}}}_{{{{{{\rm{C}}}}}}\triangle 1-9}^{{{{{{\rm{C}}}}}}}$ (Fig. S16a–d). We tested a matrix of ZF-DdCBE pairs encompassing all 175 possible combinations of one half of a ZF-DdCBE pair carrying canonical DddA^N^ or ${{{{{{\rm{DddA}}}}}}}_{{{{{{\rm{C}}}}}}\triangle 1-6}^{{{{{{\rm{N}}}}}}}$, and the second half of a ZF-DdCBE pair carrying either canonical DddA^C^, ${{{{{{\rm{DddA}}}}}}}_{{{{{{\rm{N}}}}}}\triangle 1-15}^{{{{{{\rm{C}}}}}}}$ or ${{{{{{\rm{DddA}}}}}}}_{{{{{{\rm{C}}}}}}\triangle 1-9}^{{{{{{\rm{C}}}}}}}$. We observed complete loss of on-target editing upon C-terminal truncation of DddA^N^ by more than five residues, N-terminal truncation of DddA^C^ by more than 14 residues, or C-terminal truncation of DddA^C^ by more than eight residues (Fig. S16e, g). Importantly, shorter truncations displayed a smooth, gradual decrease in on-target editing concomitant with a faster decline in off-target editing (Fig. S16f, h). We visualized these data in a XY-plot (Fig. 3d) and identified combinations that are left-shifted from the canonical ZF-DdCBE pair (reflecting lower off-target editing) while remaining as high on the Y-axis as possible (reflecting high on-target editing). The combination of ${{{{{{\rm{DddA}}}}}}}_{{{{{{\rm{C}}}}}}\triangle 3}^{{{{{{\rm{N}}}}}}}$ with ${{{{{{\rm{DddA}}}}}}}_{{{{{{\rm{N}}}}}}\triangle 5}^{{{{{{\rm{C}}}}}}}$ conferred a 3.1-fold reduction in off-target editing accompanied by only a 1.2-fold reduction in on-target editing compared to the canonical ZF-DdCBE pair. These results demonstrate that truncation of the split DddA halves can reduce ZF-DdCBE off-target editing while maintaining efficient on-target editing.

As an alternative or addition to truncating DddA^N^ and DddA^C^ to reduce ZF-DdCBE off-target editing, we also investigated the effects of shifting the position of the canonical G1397 split site within DddA to create split DddA halves with a longer DddA^N^ and a shorter DddA^C^, but did not observe better results than can be achieved by truncation alone (Fig. S17, Supplementary Note 2).

### Installing DddA point mutations to enhance ZF-DdCBE specificity

Second, we introduced point mutations into DddA^C^ in an effort to weaken the binding association between DddA^N^ and DddA^C^. We tested a series of 28 ZF-DdCBE constructs conducting Ala scanning mutagenesis across each position within DddA^C^ (Fig. 3e). We found that mutations such as K5A, R6A, G7A, T9A, V14A, T16A, N18A, and P25A led to reductions in off-target editing compared to canonical DddA^C^, without or with only modest reductions in on-target editing. In particular, N18A and P25A reduced average off-target editing by 10.6 and 1.4-fold, while retaining 80% or 112% of on-target editing compared to canonical DddA^C^, respectively.

Since Ala point mutations represent the deletion of side-chain interactions compared to the canonical protein, we hypothesized that the introduction of actively destabilizing mutations might further weaken the binding affinity between split DddA halves and reduce ZF-DdCBE off-target editing through a different mechanism. To investigate the effects of introducing positively charged residues into DddA^C^, we tested a series of 27 ZF-DdCBE constructs conducting Lys scanning mutagenesis across each position within DddA^C^ (Fig. S18a). We found that mutations T12K, V14K, N18K, and P25K each reduced off-target editing compared to canonical DddA^C^, without or with only modest reductions in on-target editing. For example, N18K reduced average off-target editing by 3.2-fold while retaining the same on-target editing as canonical DddA^C^.

We next investigated whether introducing a negatively charged mutation into DddA^C^ might reduce ZF-DdCBE off-target editing differently to positively charged mutations. We tested a series of 59 ZF-DdCBE constructs conducting either Asp or Glu scanning mutagenesis across each position within DddA^C^ (Fig. S18b, c). Our results identified the best-performing mutations as N20D, N20E, P25D, and P25E. For example, P25D reduced average off-target editing by 5.6-fold while retaining 88% of on-target editing compared to canonical DddA^C^. Collectively, these results suggest that introducing mutations into DddA^C^ that weaken the association between DddA^N^ and DddA^C^ can reduce off-target editing by ZF-DdCBEs while maintaining efficient on-target editing.

### Introducing negative charge at the termini of DddA to enhance ZF-DdCBE specificity

As a third approach to decreasing ZF-DdCBE off-target editing, we introduced negatively charged residues upstream or downstream of the split DddA halves to increase electrostatic repulsion and weaken their association. The G1397 split site in DddA is predicted to position the C-terminus of DddA^N^ and the N-terminus of DddA^C^ adjacent upon heterodimerization. In addition, the N-termini of DddA^C^ and DddA^N^ are predicted to be in close proximity (Fig. S16a). We created split DddA variants in which three, six, or nine residues in the 13-amino acid Gly/Ser-rich flexible linker upstream of DddA^N^ and DddA^C^ were mutated to either Glu or Asp residues (Fig. S19a). We also created variants in which three, six or nine Glu or Asp residues were inserted into the Gly/Ser-rich flexible linker downstream of DddA^N^. We tested 60 different ZF-DdCBE pairs with increasing levels of electrostatic repulsion, and identified combinations that improved target specificity (Fig. S19b, c). For example, variant D-6-GS+D-6-GS which has six Asp residues upstream of both DddA^N^ and DddA^C^ reduced average off-target editing by 2.0-fold while retaining 99% of on-target editing compared to the canonical ZF-DdCBE architecture. These results demonstrate that changes to the ZF-DdCBE architecture in regions outside DddA designed to weaken the association between DddA^N^ and DddA^C^ can also be used to reduce off-target editing.

### Capping with catalytically inactivated DddA^N^ to enhance ZF-DdCBE specificity

Lastly, we hypothesized that a catalytically impaired DddA^N^ fragment localized to DddA^C^ could reduce off-target ZF-DdCBE editing by competitively inhibiting the spontaneous intermolecular reassembly of DddA^N^ and DddA^C^ in the absence of binding to adjacent DNA half-sites. We first created a catalytically dead form of DddA^N^ (designated dDddA^N^) by installing the E1347A mutation into DddA^N^ and confirmed its inactivity in HEK293T cells (Fig. S20a). We explored whether fusing dDddA^N^ downstream of DddA^C^ could promote dDddA^N^ and DddA^C^ association in the absence of target DNA engagement, yet still support robust on-target editing when both ZF-DdCBE pairs are localized at the target site. We tested a series of ten ZF-DdCBE constructs in which dDddA^N^ was fused downstream of DddA^C^ using Gly/Ser-rich flexible linkers of varying length, either before or after the UGI domain, and either containing or omitting the additional two mutations T1380I and E1396K (Fig. S20b). Constructs preUGILink6dDddA and preUGILink6dDddI2K reduced average off-target editing by 3.4 and 14-fold while retaining 100% and 71% on-target editing compared to canonical ZF-DdCBE architecture (Fig. S20c). Our results demonstrate that C-terminal fusion of dDddA^N^ to DddA^C^ successfully produced ZF-DdCBEs with significantly reduced off-target editing profiles while maintaining efficient on-target editing. These findings validate an alternative approach to limiting ZF-DdCBE off-target editing that uses competitive inhibition between split deaminase halves rather than weakening their binding interaction.

### Combining multiple strategies to reduce ZF-DdCBE off-target editing

Having established four different approaches to reduce ZF-DdCBE off-target editing, we next investigated whether these approaches could be combined additively to create variants with even better specificity profiles (Fig. S21, Supplementary Note 3). To define a final set of high-specificity (HS) ZF-DdCBE variants, we created a shortlist of the top-performing single point mutations (N18K, N20E, P25A, P25K), truncations (${{{{{{\rm{DddA}}}}}}}_{{{{{{\rm{C}}}}}}\triangle 3}^{{{{{{\rm{N}}}}}}}$, ${{{{{{\rm{DddA}}}}}}}_{{{{{{\rm{N}}}}}}\triangle 5}^{{{{{{\rm{C}}}}}}}$) and dDddA^N^ architectures (preUGILink6dDddA, preUGILink13dDddA) and tested 35 combinations of their specificity-enhancing changes (Fig. 3f). From these results we selected a set of five variants that offer a balance between high on-target editing and low off-target editing, designated HS1 to HS5 (HS1 = N18K, HS2 = N18K + P25A, HS3 = N18K + P25K, HS4 = ${{{{{{\rm{DddA}}}}}}}_{{{{{{\rm{C}}}}}}\triangle 3}^{{{{{{\rm{N}}}}}}}$+ N18K + P25A, and HS5 = ${{{{{{\rm{DddA}}}}}}}_{{{{{{\rm{C}}}}}}\triangle 3}^{{{{{{\rm{N}}}}}}}$+ N18K + P25K.) HS1, HS2, HS3, HS4, and HS5 reduced average off-target editing by 4.0-, 10-, 18-, 66-fold, and down to background levels, while retaining 98%, 84%, 64%, 47%, and 27% on-target editing, respectively, compared to the canonical ZF-DdCBE pair. The HS variants we selected contain only mutations and truncations that display a greatly improved specificity profile yet are smaller or require no increase in protein size compared to canonical ZF-DdCBEs. We introduced our HS variants into the v7 ZF-DdCBE architecture and also removed the additional copy of mitochondrially targeted UGI expressed in trans, which we found to have minimal effect on on-target editing efficiency, designating these resulting high-specificity variants v8^HS1^ to v8^HS5^ (Fig. 1a).

To demonstrate that these HS variant-containing v8 advancements are generally applicable to ZF-DdCBE pairs targeting any site of interest in mtDNA and are transferrable to N-terminal ZF-DdCBE architectures, we tested all five HS variants in the context of an additional eight 3ZF+3ZF v8 ZF-DdCBE pairs targeting eight different target sites across five mitochondrial genes (Fig. S22). We note that six of these eight pairs feature an N-terminal ZF-DdCBE architecture in which split DddA is fused N-terminally relative to the ZF array. Our results showed that v8^HS1^ to v8^HS5^ reduced off-target editing at all eight sites by an average of 2.3-, 7.4-, 13-, 22- and 37-fold compared to v7, while supporting on-target editing efficiencies of 126%, 98%, 78%, 66% and 48% that of v7, respectively. Interestingly, at several sites our HS variants not only reduced off-target editing as expected but also increased on-target editing relative to v7. These results confirm that the HS variants we identified support improved ZF-DdCBE specificity profiles across a variety of different mitochondrial sites, and across canonical or N-terminal DddA ZF-DdCBE architectures. In particular, v8^HS1^ shows generally superior performance relative to v7 (an average 2.3-fold reduction in off-target editing with little or no reduction in on-target editing across all eight sites tested), and we recommend using v8^HS1^ in preference to v7.

Lastly, we used the v8^HS1^ variant in nine ZF-DdCBE pairs derived from mtDNA-targeting ZFD pairs recently reported by Kim and coworkers^29^. Averaged across the nine pairs tested, v8^HS1^ variants reduced average off-target editing by 4.1-fold while retaining 90% on-target editing efficiency relative to v7 ZF-DdCBEs (Fig. S23). Moreover, v8^HS1^ ZF-DdCBEs supported an average 3.1-fold higher on-target editing compared to ZFDs, concomitant with a 2.6-fold increase in average off-target editing. Our optimized v8^HS1^ ZF-DdCBEs offer substantial improvements in on-target editing over ZFDs, although these are also accompanied by increased off-target editing.

Collectively, these results demonstrate that strategies to reduce off-target editing caused by spontaneous split DddA reassembly can be integrated to engineer high-specificity ZF-DdCBE variants with reduced off-target editing and efficient on-target editing.

### Installing disease-associated edits in mtDNA in cells in vitro

**Fig. 4:**
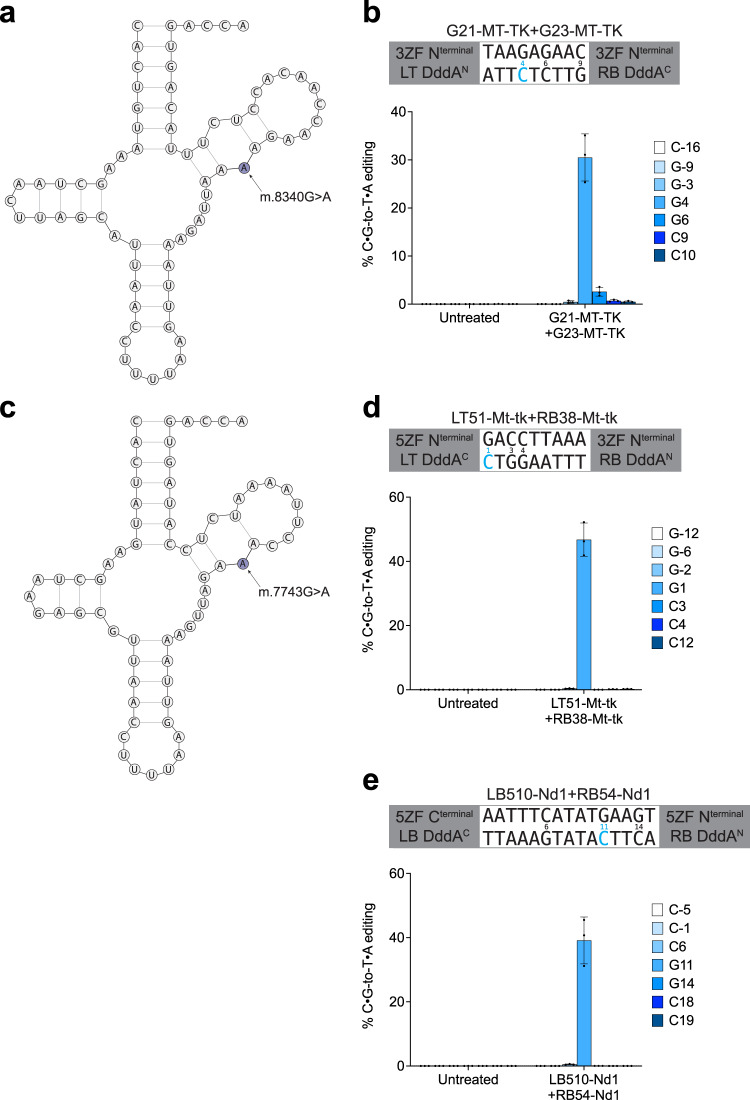
ZF-DdCBEs install pathogenic mutations in cultured cells in vitro. **a** The m.8340G>A mutation in human *MT-TK* disrupts the T-arm of mt-tRNA^Lys^. **b** Mitochondrial DNA base editing efficiencies of HEK293T cells treated with an optimized ZF-DdCBE pair designed to install m.8340G>A. **c** The m.7743G>A mutation in mouse *Mt-tk* disrupts the T-arm of mt-tRNA^Lys^. **d** Mitochondrial DNA base editing efficiencies of C2C12 cells treated with an optimized ZF-DdCBE pair designed to install m.7743G>A. **e** Mitochondrial DNA base editing efficiencies of C2C12 cells treated with an optimized ZF-DdCBE pair designed to install m.3177G>A. For (**b**, **d** and **e**), values and errors reflect the mean ± s.d. of *n* = 3 independent biological replicates. For each site the DNA spacing region, split DddA orientation, ZF array lengths, and ZF-targeted DNA strands (LT left top, LB left bottom, RB right bottom) are shown, and the cytosine with the highest editing efficiency is colored in blue. Source data are provided as a Source Data file.

To demonstrate the utility of ZF-DdCBEs to install disease-associated mutations, we designed ZF-DdCBEs to install the m.8340G>A mutation within *MT-TK* in HEK293T cells. This mutation is associated with mitochondrial myopathy and retinopathy, creating a mismatch in the T-arm of mt-tRNA^Lys^ that impairs mitochondrial translation^44–47^ (Fig. 4a). We tested a panel of three left 3ZF ZF-DdCBEs with five right 3ZF ZF-DdCBEs in both deaminase orientations (DddA^N^+DddA^C^ and DddA^C^+DddA^N^), forming a total of 30 different combinations in v7 architecture (Fig. S24a). Our top initial hit was able to install the m.8340G>A edit with an efficiency of 11% (Fig. S24b). For this best-performing ZF-DdCBE combination, we tested extending each 3ZF to 4ZF or 5ZF but observed no improvement in on-target editing (Fig. S24c). By testing alternative ZF scaffolds we found that v7^AGKS^ architecture improved editing results, and this optimized ZF-DdCBE pair installed the m.8340G>A mutation with an efficiency of 31% (Fig. 4b). No substantial bystander editing was observed in the spacing region aside from 2.6% editing at position m.8342, which would create an additional mismatch in the mt-tRNA^Lys^ T-arm and be expected to further magnify the disease phenotype. These results show that ZF-DdCBEs can install targeted disease-associated mutations in human cells with high efficiency and precision, creating model cell lines for the study of human mitochondrial genetic diseases.

We next investigated whether ZF-DdCBEs could be used in other mammalian cell lines to create biological models of human genetic diseases. Towards creating a mouse model of the human m.8340G>A genetic disease, we explored installing the m.7743G>A mutation in mouse C2C12 cells (Fig. 4c). Because human *MT-TK* and mouse *Mt-tk* genes share only 60% sequence identity, this lack of sequence conservation necessitated designing and optimizing a new set of ZF-DdCBE pairs in the murine context. We tested a panel of 20 left 3ZF ZF-DdCBEs with 19 right 3ZF ZF-DdCBEs in both deaminase orientations, forming 760 pairwise combinations in v7^AGKS^ architecture (Fig. S25a). We identified 27 ZF-DdCBE pairs able to install the desired edit with efficiencies ranging from 5% to 23% (Fig. S25b). We optimized these pairs by extending each 3ZF to 4ZF, 5ZF, or 6ZF where possible in addition to testing alternative ZF scaffolds (Supplementary Note 4). We selected an optimized ZF-DdCBE pair (LT51-Mt-tk+RB38-Mt-tk) that offers a good balance between high on-target activity and low bystander or off-target editing. This final 5ZF+3ZF v7^AGKS^ ZF-DdCBE pair exhibited a 1.6-fold improvement relative to its corresponding 3ZF+3ZF pair, installing the m.7743G>A mutation at an efficiency of 47% and with excellent precision (Fig. 4d). We confirmed that the v8^HS1^ variant of this ZF-DdCBE pair decreased off-target editing by 14-fold and 10-fold, while retaining 37% and 48% on-target editing compared to v7 and v8, respectively (Fig. S25g). Collectively, these results show that ZF-DdCBEs can be used to create biological models of human genetic disease and install targeted disease-associated mutations in different cell lines from different organisms with good efficiency and precision.

As a second demonstration of using ZF-DdCBEs to create biological models of human genetic diseases, we installed the m.3177G>A mutation in mouse C2C12 cells, creating a missense E143K mutation in the mitochondrial *Nd1* gene associated with Leber’s hereditary optic neuropathy (LHON)^48,49^ (Fig. S26g). We tested a panel of 19 left 3ZF ZF-DdCBEs with 25 right 3ZF ZF-DdCBEs in both deaminase orientations, forming 950 pairwise combinations in v7^AGKS^ architecture (Fig. S26a). We identified 26 ZF-DdCBE pairs able to install the desired edit with efficiencies ranging from 5% to 20% (Fig. S26b). We optimized these pairs by extending each 3ZF to 4ZF, 5ZF, or 6ZF where possible in addition to testing alternative ZF scaffolds (Supplementary Note 5). We selected a pair (LB510-Nd1+RB54-Nd1) that showed a good balance between high on-target activity and low bystander or off-target editing. This final 5ZF+5ZF v7^AGKS^ ZF-DdCBE pair exhibited a 1.9-fold improvement relative to its corresponding 3ZF+3ZF pair, installing the m.3177G>A mutation at an efficiency of 39% and with excellent precision (Fig. 4e). To reduce off-target editing, we tested v8^HS^ variants of this ZF-DdCBE pair and observed that v8^HS1^ reduced average off-target editing by 6.8-fold and 5.9-fold, while retaining 27% and 32% on-target editing compared to v7 and v8 respectively (Fig. S26f). Collectively, these results establish ZF-DdCBEs as a useful tool for the creation of biological models of human genetic diseases through the efficient and precise installation of targeted disease-associated mutations.

### ZF-DdCBEs enable base editing of nuclear DNA

To test whether ZF-DdCBEs are capable of mediating targeted C•G-to-T•A conversion in nuclear DNA, we converted validated mitochondrial ZF-DdCBEs into nuclear ZF-DdCBEs. We selected sites in mtDNA that were edited by optimized 3ZF+3ZF ZF-DdCBEs with high efficiency in HEK293T cells and searched the human nuclear genome for corresponding sites with high sequence similarity. We identified nuclear sites that share conserved ZF binding sites with no mismatches, are separated by a spacing region within ±2 bp in length compared to the mtDNA target’s spacing region, and contain TC dinucleotides at similar positions within the spacing region compared to the target nucleotide(s) efficiently edited in mtDNA (Fig. S27).

**Fig. 5:**
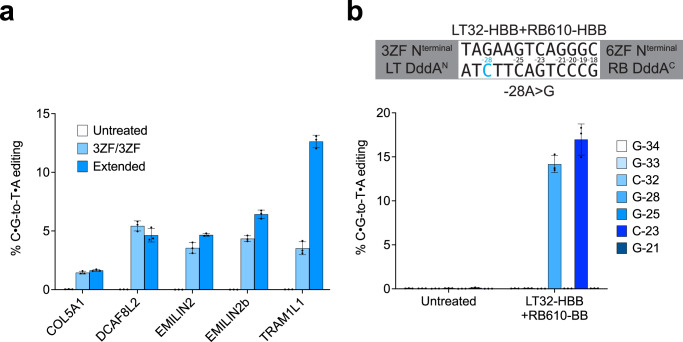
ZF-DdCBEs enable base editing of nuclear DNA. **a** Nuclear DNA base editing efficiencies of HEK293T cells treated with five 3ZF+3ZF nuclear-targeted ZF-DdCBE pairs, or ZF-DdCBE variants with extended ZF arrays. ZF-DdCBE pairs were designed to install edits within or nearby nuclear genes *COL5A1*, *DCAF8L2*, *EMILIN2*, *EMILIN2*, and *TRAM1L1*, respectively. The editing efficiencies shown are for the most efficiently edited C•G within the spacing region. **b** Nuclear DNA base editing efficiencies of HEK293T-HBB cells treated with an optimized ZF-DdCBE pair designed to correct the *HBB* −28A>G mutation. The DNA spacing region, split DddA orientation, ZF array lengths, and ZF-targeted DNA strands (LT left top, RB right bottom) are shown, and the pathogenic cytosine is colored in blue. For (**a** and **b**), values and errors reflect the mean ± s.d. of *n* = 3 indepe*n*dent biological replicates. Source data are provided as a Source Data file.

To create nuclear-targeted ZF-DdCBEs, we adapted our mitochondria-targeted v7 ZF-DdCBE architecture by replacing the N-terminal MTS and NES sequences with four NLS sequences (two SV40 bipartite NLS and two cMyc NLS), and removing the additional copy of mitochondrially targeted UGI expressed in trans. We tested four nuclear-targeted 3ZF+3ZF ZF-DdCBE pairs at five sites in nuclear DNA and observed editing efficiencies in HEK293T cells ranging from 1-5% across the five sites tested. We tested extending each 3ZF array to 4ZF, 5ZF, or 6ZF and observed improvements in editing efficiency for four of the five pairs tested, with on-target editing efficiencies ranging from 2–13% (Fig. 5a). These results establish that ZF-DdCBEs support all-protein nuclear base editing, even when designing ZFs using the simple modular assembly approach.

To demonstrate the ability of ZF-DdCBEs to correct disease-causing mutations in nuclear DNA, we corrected the −28A>G mutation in the promoter region of the human *HBB* gene that causes β-thalassemia^50^. We tested a panel of 24 left 3ZF ZF-DdCBEs with 24 right 3ZF ZF-DdCBEs in both deaminase orientations (Fig. S28a) in HEK293T-HBB cells that have a lentivirus-integrated 200-bp fragment of the mutated *HBB* promoter sequence locus^51^. We identified eight 3ZF+3ZF ZF-DdCBE pairs that performed the desired edit with 1–3% efficiencies (Fig. S28b). We optimized these pairs by extending each 3ZF to 4ZF, 5ZF, or 6ZF, and found that the most efficient ZF-DdCBE pair installed the desired edit with an editing efficiency of 14%, a 6.8-fold improvement relative to the unoptimized 3ZF+3ZF pair, together with 17% bystander editing corresponding to −23C>T (Fig. 5b). This bystander mutation lies downstream of the *HBB* promoter’s non-canonical TATA-box (CATAAA) bound by transcription factor TFIID^52^, and is not known to be associated with any globinopathy^53^. Collectively, these results demonstrate that ZF-DdCBEs can correct pathogenic mutations in nuclear DNA, albeit less efficiently than canonical nuclease base editors. Additional ZF optimization may further increase editing efficiency, as we anticipate that ZF-DdCBE nuclear editing efficiency will strongly correlate with ZF binding affinity and specificity.

### In vivo base editing of pathogenic target sites in mtDNA

An important advantage of the reduced size of ZF-DdCBEs compared to TALE-based DdCBEs is their ability to be packaged into a single AAV capsid for in vivo delivery. To validate that ZF-DdCBE pairs could be expressed as a single operon, we created rAAV2-CMV expression vectors^54^ encoding v8^HS1^ ZF-DdCBE pairs designed to install either the murine m.7743G>A or m.3177G>A mutation, and expressed under a single CMV promoter using a ribosomal skipping P2A peptide between each ZF-DdCBE half. We selected v8^HS1^ variants, which offer an improved specificity profile over v7, to reduce off-target editing in vivo. We verified that these constructs retained editing activity in C2C12 cells, installing either m.7743G>A or m.3177G>A with an editing efficiency of 38% and 16%, respectively (Figs. S25e, S26d). To facilitate bacterial cloning, we installed into the vector backbone a cassette for constitutive bacterial expression DddI, the natural protein inhibitor of DddA, at a location that would not be packaged into AAV genomes. These results demonstrate that ZF-DdCBE pairs can mediate good editing efficiency when expressed as a single gene (2.4 and 2.5 kb in length, respectively) that is much smaller in size than the AAV packaging limit of ~4.7 kb, suggesting that ZF-DdCBEs might be suitable for single AAV-mediated delivery (Fig. S1).

**Fig. 6:**
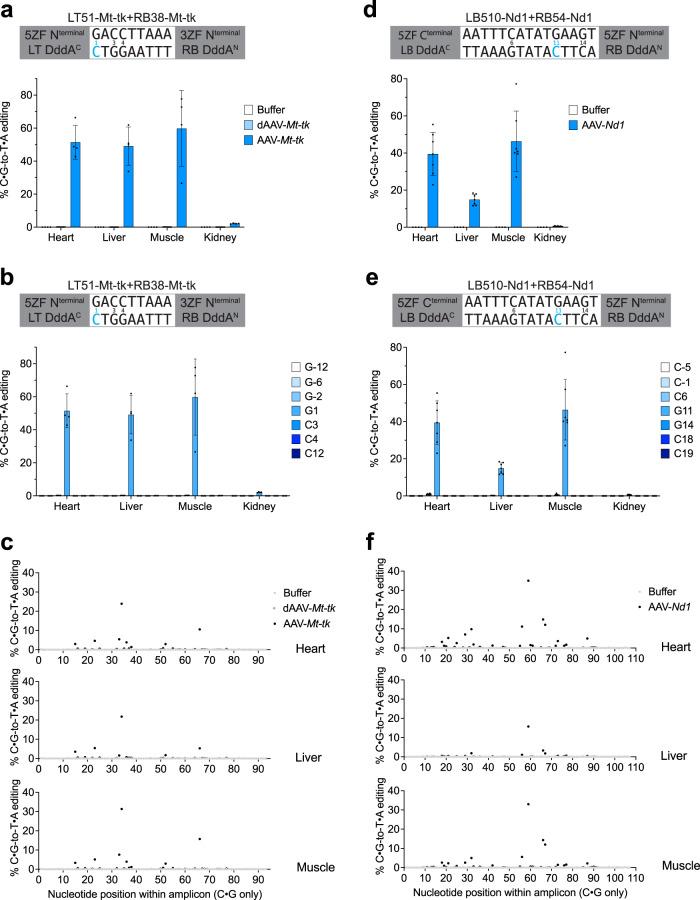
In vivo base editing of pathogenic sites in mtDNA. **a** Mitochondrial DNA base editing efficiencies installing m.7743G>A of tissue samples from mice treated with buffer, dAAV-*Mt-tk*, or AAV-*Mt-tk*. **b** Mitochondrial DNA base editing efficiencies of tissue samples from AAV-*Mt-tk*-treated mice. **c** Off-target editing efficiencies within representative mitochondrial off-target amplicon OT8 of tissue samples from mice treated with buffer, dAAV-*Mt-tk*, or AAV-*Mt-tk*. **d** Mitochondrial DNA base editing efficiencies installing m.3177G>A of tissue samples from mice treated with buffer or AAV-*Nd1*. **e** Mitochondrial DNA base editing efficiencies of tissue samples from AAV-*Nd1*-treated mice. **f** Off-target editing efficiencies within representative mitochondrial off-target amplicon OT7 of tissue samples from mice treated with buffer, or AAV-*Nd1*. For (**a** and **b**), values and errors reflect the mean ± s.d. of *n* = 4, 4 and 7 for mice treated with buffer, AAV-*Mt-tk*, or dAAV-*Mt-tk*, respectively. For (**c**), values reflect the mean of *n* = 4, 4 and 7 for mice treated with buffer, AAV-*Mt-tk*, or dAAV-*Mt-tk*, respectively. For (**d** and **e**), values and errors reflect the mean ± s.d. of *n* = 4 and 7 for mice treated with buffer or AAV-*Nd1*, respectively. For (**f**), values reflect the mean of *n* = 4 and 7 for mice treated with buffer or AAV-*Nd1*, respectively. Source data are provided as a Source Data file.

To investigate the performance of ZF-DdCBEs in vivo, after recombinant AAV2/9 production we delivered 7.5 × 10^11^ viral genomes (AAV-*Mt-tk* or AAV-*Nd1*, encoding v8^HS1^ ZF-DdCBE pairs installing m.7743G>A or m.3177G>A, respectively) into newborn P1 mice by intravenous injection and harvested tissue samples for DNA sequencing after 14–30 days. We chose this high dose in order to maximise our ability to detect in vivo editing in this proof-of-principle experiment. We observed robust editing in the heart, liver and quadriceps skeletal muscle, with average on-target editing activities of 51 ±10%, 49 ±12%, and 60 ±23% for AAV-*Mt-tk* and 39 ±12%, 15 ±3%, and 46 ±16% for AAV-*Nd1*, respectively, and with editing profiles similar to those observed in C2C12 cells in vitro (Fig. 6a, b, d, e). As a negative control, we did not observe editing following AAV delivery encoding the *Mt-tk*-targeting ZF-DdCBE pair containing the DddA-inactivating E1347A mutation (dAAV-*Mt-tk*) (Fig. 6a).

To assess in vivo off-target editing, we performed targeted amplicon sequencing at predicted ZF off-target sites. For mice treated with AAV-*Nd1* we sequenced seven amplicons that contain the top eight off-target ZF binding sites in mtDNA as predicted by sequence similarity (four off-target sites for the left 5ZF array containing three nucleotide mismatches and four off-target sites for the right 5ZF array containing either three or four nucleotide mismatches). For mice treated with AAV-*Mt-tk* we sequenced seven amplicons that contain 14 off-target ZF binding sites in mtDNA as predicted by sequence similarity (eight off-target sites for the left 5ZF array containing three or four nucleotide mismatches and six off-target sites for the right 3ZF array containing one nucleotide mismatch). We observed off-target editing at C•G base pairs scattered across each predicted off-target site, typically with efficiencies ≥10-fold lower that of the on-target edit in the same tissues, although some C•G base pairs flanking the predicted off-target ZF binding sites were edited more efficiently (Figs. 6c, f, S29, S30). The in vivo durability of AAV, which can support ZF-DdCBE expression throughout the 14–30 days of the experiment^55^, likely resulted in the accumulation of these off-target edits. The relatively high level of off-target editing we observed currently limits the use of ZF-DdCBEs for therapeutic applications. We anticipate that the use of transient mRNA or RNP delivery methods instead of AAV, or recently developed methods to limit the duration of AAV expression^56–58^, will reduce off-target editing in vivo. These results collectively demonstrate that ZF-DdCBEs enable efficient in vivo editing of mtDNA via single-AAV delivery, and can be used in mice to install disease-associated point mutations in a variety of tissues.

## Discussion

We created and extensively optimized ZF-DdCBEs capable of base editing both mitochondrial and nuclear DNA. ZF-DdCBEs have fewer design limitations and are substantially smaller than TALE-containing DdCBEs. We demonstrated that this size reduction facilitates packaging within a single AAV9 capsid for efficient in vivo base editing of mtDNA, in contrast with dual-AAV approaches used for the in vivo delivery of TALE-based DdCBEs^59^. We also identified approaches to reduce off-target editing by impeding spontaneous split DddA reassembly.

Since shorter ZF arrays are less expensive to construct, for researchers designing ZF-DdCBEs we suggest starting with pairs of 3ZF+3ZF ZF-DdCBEs, which can support efficient editing in mitochondria. For nuclear targets we suggest starting with longer 5ZF+5ZF ZF-DdCBEs. For maximum on-target editing efficiency, we recommend starting with v8 architecture using ZF scaffold X1. We recommend testing a panel of ZF-DdCBEs in both split DddA orientations (DddA^N^+DddA^C^ and DddA^C^+DddA^N^) for each target of interest. We anticipate that following these steps will enable the identification of efficient ZF-DdCBE pairs for a variety of applications. Optionally, researchers can subsequently test the effect of varying ZF array length from 3ZF to 6ZF for the most active ZF-DdCBE pairs identified in order to maximize editing efficiency. ZF arrays containing a higher proportion of GNN-binding modules may lead to improved ZF-DdCBE pairs (Supplementary Note 6). After identifying high-performing ZF-DdCBE pairs, researchers can consider testing alternative ZF scaffolds (AGKS, V2, V20) which may lead to additional improvements in performance, and then incorporating v8 variants HS1-HS5 when minimizing off-target editing is critical. These optional features (varying ZF array lengths, alternative ZF scaffolds, and HS variants) are recommended for researchers that require ZF-DdCBEs with optimized charcteristics.

Although straightforward, the modular assembly approach that we used for constructing ZFs (Supplementary Data 3) has a higher failure rate and can yield less potent DNA-binding ZF arrays than methods that use in vivo selection^34^. After having identified promising ZF arrays from initial ZF-DdCBE screens, more sophisticated approaches to ZF design, such as iterated library screening and selection that account for context-dependent effects^60,61^, should result in ZF-DdCBEs with more potent target binding activity and specificity.

ZF-DdCBEs have the flexibility to be designed in either N- or C-terminal architectures, offering researchers additional targeting options not available for TALE-based DdCBEs. We demonstrate that interchangeably using both canonical and N-terminal architectures allows ZF-DdCBEs to be designed to bind to either the same or opposite DNA strands around the target nucleotide(s). The diversity of ZF-DdCBE pairs we report in this study suggests that all possible configurations of split DddA orientation and N- or C-terminal architectures can work well, and demonstrates the freedom researchers have when designing ZF-DdCBEs to edit their sequence of interest. Several of the active ZF-DdCBE pairs in this study support efficient editing with much smaller spacing regions than TALE-DdCBEs, thus reducing the number of non-target cytosines within the editing window and minimizing bystander editing. Due to their smaller size it will be significantly cheaper and simpler to screen ZF-DdCBE combinations at each target site of interest compared to testing large numbers of TALE-based DdCBEs. Moreover, ZFs are highly abundant endogenous proteins in human cells, and are much less repetitive than TALEs – factors that are expected to translate into reduced immunogenicity.

We demonstrated that our optimized ZF-DdCBEs enable higher editing efficiencies than previously reported ZFDs^29^. Given that mitochondrial disease phenotype is dependent on the level of heteroplasmy, the ability to install mutations within mtDNA at high efficiency is important to accurately model the consequences of mitochondrial genetic disease.

ZF-DdCBEs lead to higher levels of off-target editing, even after our extensive optimizations, than TALE-DdCBEs or CBEs. As a result, the off-target editing displayed by ZF-DdCBEs in their current form is likely to be too high for therapeutic applications, especially at high doses and over sustained expression levels. Future efforts to further decrease off-target editing by ZF-DdCBEs may enable ZF-DdCBEs to become therapeutically relevant for the correction of pathogenic genetic mutations. We anticipate that the delivery of ZF-DdCBEs in mRNA or protein form should reduce off-target editing by limiting the exposure of cells to ZF-DdCBEs, as has been shown for nucleases and base editors^29,62–64^. Alongside such changes in delivery modality, we anticipate that changes in dosing, exploring the effects of delivering higher specificity variants (such as v8^HS2^ to v8^HS5^), or further ZF-DdCBE engineering efforts each offer potential routes to tackling this challenge. As Lim et al. reported improved specificity by making substitutions in the ZF scaffold^29^, it may be promising to investigate the effects of using the different ZF scaffolds we identify here on off-target editing. In their present form, the optimized ZF-DdCBEs we describe here can be useful tools for researchers studying mutations in mitochondrial DNA and for modelling the effect of making mutations in both cultured cells and in animals.

## Methods

### Ethics statement

This research complies with all relevant ethical regulations. All animal experiments were carried out in accordance with the UK Animals (Scientific Procedures) Act 1986 (Procedure Project Licence: P6C20975A) and EU Directive 2010/63/EU and authorized by the University of Cambridge Animal Welfare and Ethical Review Body.

### General methods and molecular cloning

All plasmids were constructed by Gibson assembly using NEBuilder HiFi DNA Assembly Master Mix (New England Biolabs) or synthesized and cloned by Twist Biosciences and transformed into MachOne T1^R^ chemically competent *E. coli* cells (Thermo Fisher Scientific). DNA primers were ordered from Integrated DNA Technologies. PCR primer oligonucleotide sequences are provided in Supplementary Data 3. PCR was performed using PrimeSTAR GXL DNA Polymerase (Takara Bio). Synthetic DNA was ordered as eblock or gblock fragments from Integrated DNA Technologies (IDT). Codon optimization was performed either manually or using IDT’s Codon Optimization Tool. Plasmid DNA was amplified by rolling circle amplification using a TempliPhi Amplification Kit (Cytiva) prior to Sanger sequencing for sequence confirmation. Plasmids were purified using QIAprep Spin Miniprep kits (Qiagen) and quantified using a NanoDrop One spectrophotometer (Thermo Fisher Scientific).

### General mammalian cell culture conditions

HEK293T (CRL-3216) and C2C12 (CRL-1772) cells were purchased from American Type Culture Collection (ATCC) and cultured and passaged in DMEM supplemented with GlutaMAX (Thermo Fisher Scientific) and 10% (v/v) FBS (Gibco, qualified). Cells were incubated, maintained and cultured at 37 °C with 5% CO_2_. Cell lines were authenticated by their respective suppliers and tested negative for mycoplasma.

### Tissue culture transfection and genomic DNA extraction

Cells were seeded on 48-well poly-D-lysine-coated plates (Corning), or 48-well collagen-coated plates (Corning) where specified, in a volume of 250 µl per well at a density of 6 × 10^4^ cells/ml for human cells or a density of 2 × 10^4^ cells/ml for C2C12 cells. 24 h after seeding, cells were transfected with a total of 25 µl lipofection mix in Opti-MEM (Thermo Fisher Scientific) containing 1 µg plasmid DNA (500 ng each ZF-DdCBE) and 1.5 µl Lipofectamine 2000 (Thermo Fisher Scientific) at approximately 40% confluency. Cells were harvested 3 days after transfection for genomic DNA (gDNA) extraction. Medium was removed and cells were washed once with PBS (Thermo Fisher Scientific). Cells were lysed by the addition of 80 µl freshly prepared lysis buffer (10 mM Tris-HCl (pH 8.0), 0.05% SDS and 25 µg/ml proteinase K (Thermo Fisher Scientific)) and incubated at 37 °C for 1 h before proteinase K was inactivated at 80 °C for 30 min. Genomic DNA was stored at −20 °C until used.

### High-throughput DNA sequencing of genomic DNA samples

Genomic sites of interest were amplified from genomic DNA samples and sequenced on an Illumina MiSeq. Amplification primers containing Illumina forward and reverse adapters (Supplementary Data 1) were used for a first round of PCR (PCR1) to amplify the genomic region of interest. 25 µl PCR1 reactions were performed using Phusion Hot Start II High-Fidelity DNA Polymerase (Thermo Fisher Scientific) with 2 µl genomic DNA extract and supplemented with 0.5X SYBR Green I (Thermo Fisher Scientific), and monitored by quantitative PCR (CFX96, Bio-Rad). The PCR1 protocol was 98 °C for 120 s, then 30 cycles of 98 °C for 10 s, 62 °C for 20 s and 72 °C for 30 s, and followed by a final 72 °C extension for 120 s. Unique Illumina barcodes were added to each sample in a secondary PCR (PCR2). 25 µl PCR2 reactions were performed using Phusion Hot Start II High-Fidelity DNA Polymerase (Thermo Fisher Scientific) with 2 µl unpurified PCR1 product. The PCR2 protocol was 98 °C for 120 s, then 10 cycles of 98 °C for 10 s, 61 °C for 20 s and 72 °C for 30 s, and followed by a final 72 °C extension for 120 s. PCR2 products were pooled by common amplicons and purified by gel electrophoresis with a 2% agarose gel using a QIAquick Gel Extraction kit (Qiagen). DNA was quantified using a Qubit dsDNA High Sensitivity Assay kit (Thermo Fisher Scientific) and sequenced using an Illumina MiSeq with single-end reads, using Illumina MiSeq Control software 3.1. Sequencing results were computed with a minimum sequencing depth of approximately 10,000 reads per sample.

### Analysis of high-throughput sequencing data for targeted amplicon sequencing

Sequencing reads were demultiplexed using MiSeq Reporter (Illumina) and analyzed by amplicon using CRISPResso2 (version 2.1.3)^65^ using default parameters. Supplementary Data 1 contains a list of amplicon sequences used for alignment. A cleavage offset of −8 was used and a 16 bp spacing region between ZF-DdCBEs was supplied in place of the input sgRNA sequence. A 10 bp window was used to quantify indels centered around the middle of the spacing region between ZF-DdCBEs. The output file Nucleotide_percentage_summary.txt was imported into Microsoft Excel (Microsoft) for quantification of editing frequencies. Reads containing indels within the 10 bp window are excluded for calculation of editing frequencies. The output file CRISPRessoBatch_quantification_of_editing_frequency.txt was imported into Microsoft Excel (Microsoft) for calculation of indel frequencies. Indel frequencies were computed by dividing the number of aligned reads containing insertions or deletions by the total number of aligned reads. Average off-target editing efficiencies were calculated by averaging the C•G-to-T•A editing efficiency across all C•G base pairs within the amplicon. For amplicons containing the spacing region targeted by a ZF-DdCBE pair, nucleotides ±10 bp upstream and downstream of the nucleotide with the highest on-target C•G-to-T•A editing efficiency were excluded from the analysis. All graphs were plotted using Prism 8 (GraphPad).

### Bioinformatic searches

ScanProsite^66^ was used to search the human proteome for ZF-containing sequences, submitting the motif x(6)-C-x(2)-C-x(12)-H-x(3)-H-x(5) as a query to scan against the UniProtKB protein sequence database, using *Homo sapiens* as a taxonomical filter. Sequence logos were generated using WebLogo 3^67^, available online at http://weblogo.threeplusone.com/create.cgi. Nuclear sites with high sequence similarity to validated mitochondrial ZF-DdCBE targets were identified using ZFN-Site^68^, available online at https://ccg.epfl.ch/tagger/targetsearch.html. Queries used settings of zero mismatches per half-site and disallowing left and right protein homo-dimerization.

### Viral vector production and in vivo animal experiments

ZF-DdCBE-expressing rAAV2-CMV vectors were used to generate recombinant AAV2/9 viral particles at the University of North Carolina at Chapel Hill Vector Core. Mice in a C57BL/6 J background were obtained from Charles River Laboratories. The animals were maintained in a temperature- and humidity-controlled animal care facility with a 12 hour light/12 h dark cycle and free access to water and food and sacrificed by cervical dislocation. Newborn mice (postnatal day 1 – males and females) were injected with 7.5 × 10^11^ AAV particles via the temporal vein using a 30 G, 30°-beveled needle syringe. Control mice were injected with similar volumes of vehicle buffer (1X PBS, 230 mM NaCl and 5% (w/v) D-sorbitol). Samples from the heart, quadriceps, liver and kidney were snap-frozen in liquid nitrogen at sacrifice and stored at −80 °C until used. Genomic DNA from mouse tissue samples was extracted using a DNeasy Blood & Tissue kit (Qiagen).

### Statistics and reproducibility

No statistical method was used to predetermine sample size. No data were excluded from the analyses. The experiments were not randomized. No statistical analysis was performed. The Investigators were not blinded to allocation during experiments and outcome assessment. Experiments were found to be reproducible between different data sets collected across a timescale of several months.

### Reporting summary

Further information on research design is available in the Nature Portfolio Reporting Summary linked to this article.

## Supplementary information

Supplementary Information

Reporting Summary

Supplementary Data 1

Supplementary Data 2

Supplementary Data 3

Description of Additional Supplementary Files

## Data Availability

High-throughput sequencing data have been deposited in the National Center of Biotechnology Information Sequence Read Archive under accession code PRJNA835113. Plasmids encoding select ZF-DdCBEs will be made available at Addgene for distribution. Amino acid sequences of all ZF-DdCBEs in this study are provided in Supplementary Data 1. Data from all ZF-DdCBE specificity optimization experiments are provided in Supplementary Data 2. A guide for designing and cloning ZF-DdCBE pairs together with PCR primer oligonucleotide sequences are provided in Supplementary Data 3. Source data are provided with this paper.

## Source data

Source Data

## Code availability

High-throughput sequencing data was analyzed using Crispresso2 (version 2.1.3)^65^.
