# Supplementary material for: Compact zinc finger base editors that edit mitochondrial or nuclear DNA in vitro and in vivo
Source: Nat Commun. 2022 Nov 23;13:7204. doi: 10.1038/s41467-022-34784-7 (PMC9684478; doi:10.1038/s41467-022-34784-7)
Supplement: Supplementary file 1 — Supplementary Information [file 41467_2022_34784_MOESM1_ESM.pdf]

Supplementary Information

Compact zinc finger base editors that edit mitochondrial or nuclear DNA *in vitro* and *in vivo*

Julian C. W. Willis[1,2,3], Pedro Silva-Pinheiro[4], Lily Widdup[1,2,3], Michal Minczuk[4], David R. Liu[1,2,3*]

Supplementary Figure 1. All-protein base editor size comparison 4

Supplementary Figure 2. ZF-DdCBE architecture optimization 5

Supplementary Figure 3. ZF array length and positioning influences ZF-DdCBE editing efficiency 7

Supplementary Figure 4. Design of ZF-DdCBEs at $(GNN)_n$-rich sites 9

Supplementary Figure 5. Extension of ZF array length improves ZF-DdCBE editing efficiency, but including extended linkers is detrimental 10

Supplementary Figure 6. Defining new ZF scaffolds improves ZF-DdCBE editing efficiency 11

Supplementary Figure 7. Defining new ZF scaffolds derived from the human proteome 12

Supplementary Figure 8. Identifying new ZF scaffolds derived from the human proteome that improve ZF-DdCBE editing efficiency 14

Supplementary Figure 9. Identifying new ZF scaffolds derived from ZFN268(F1) and Sp1C that improve ZF-DdCBE editing efficiency 16

Supplementary Figure 10. Optimized ZF scaffolds increase ZF-DdCBE editing efficiency 17

Supplementary Figure 11. DddA mutations enhance ZF-DdCBE editing efficiency 18

Supplementary Figure 12. Optimized ZF scaffolds increase ZF-DdCBE editing efficiency 19

Supplementary Figure 13. Identifying ZF scaffolds that support the highest editing efficiency for ZFD-derived ZF-DdCBEs 20

Supplementary Figure 14. Time course of TALE-DdCBE and ZF-DdCBE editing efficiencies over time 21

Supplementary Figure 15. Amino acid sequences immediately upstream of $DddA^N$ and $DddA^C$ influence non-targeted editing activity 23

Supplementary Figure 16. DddA truncation reduces ZF-DdCBE off-target editing 24

Supplementary Figure 17. Shifting the position of the canonical G1397 split site within DddA 25

Supplementary Figure 18. Introducing point mutations within $DddA^C$ 26

Supplementary Figure 19. Introducing negative charge at the termini of DddA 27

Supplementary Figure 20. Capping with catalytically inactivated $DddA^N$ 28

Supplementary Figure 21. Combining approaches to reduce ZF-DdCBE off-target editing 30

Supplementary Figure 22. $v8^{HS}$ ZF-DdCBE variants reduce off-target editing 31

Supplementary Figure 23. Comparison between $v8^{HS1}$ ZF-DdCBEs and ZFDs 32

Supplementary Figure 24. Optimized ZF-DdCBEs install m.8340G>A in HEK293T cells 33

Supplementary Figure 25. Optimized ZF-DdCBEs install m.7743G>A in C2C12 cells 34

Supplementary Figure 26. Optimized ZF-DdCBEs install m.3177G>A in C2C12 cells 36

Supplementary Figure 27. Converting mitochondrial ZF-DdCBEs into nuclear ZF-DdCBEs 38

Supplementary Figure 28. Correction of a nuclear disease-causing mutation using ZF-DdCBEs 39

Supplementary Figure 29. Off-target editing analysis of mice treated with AAV-*Mt-tk* 40

Supplementary Figure 30. Off-target editing analysis of mice treated with AAV-*Nd1* 41

Supplementary Figure 31. Configurations and DNA sequences of spacing regions for ZF-DdCBE pairs used in this study 42

Supplementary Note 1. Defining new ZF scaffold AGKS 44

Supplementary Note 2. Shifting the position of the canonical G1397 split site within DddA 45

Supplementary Note 3. Combining approaches to reduce ZF-DdCBE off-target editing 46

Supplementary Note 4. Optimizing ZF-DdCBEs to install m.7743G>A within *Mt-tk* 47

Supplementary Note 5. Optimizing ZF-DdCBEs to install m.3177G>A within *Nd1* 48

Supplementary Note 6. Prevalence of GNN-binding modules in ZF arrays within active ZF-DdCBE pairs 49

Supplementary References 50

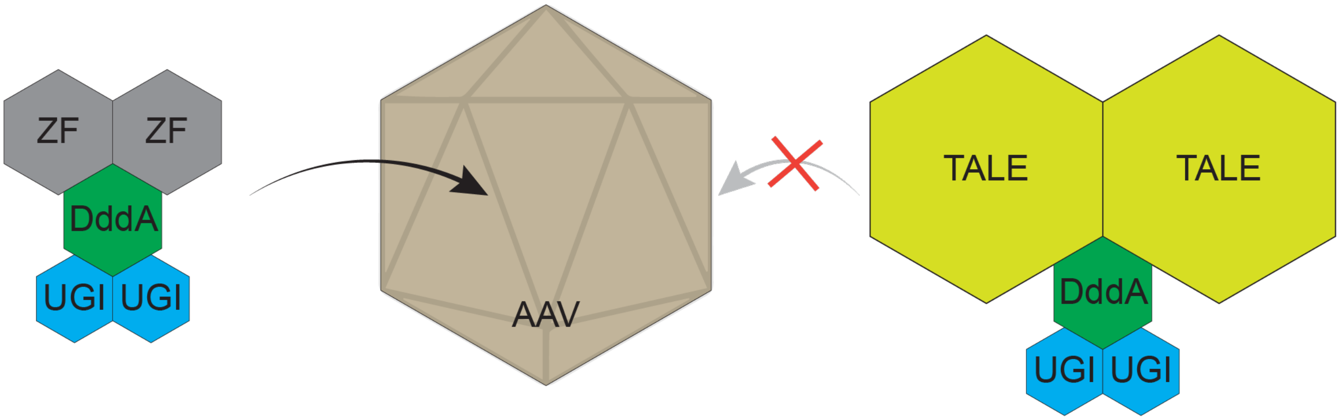

Supplementary Figure 1 | All-protein base editor size comparison. The area of each hexagon is proportional to the length of DNA sequence required to encode that protein. The total AAV packaging capacity of ~4.7 kb is represented proportionally in brown. The total size of DNA encoding a ZF-DdCBE is well below the AAV packaging capacity limit, whereas the total size of DNA encoding a TALE-DdCBE exceeds the packaging limit of a single AAV capsid. The ZF and TALE hexagons each represent a six-zinc finger (6ZF) array and an 18-repeat TALE array, respectively.

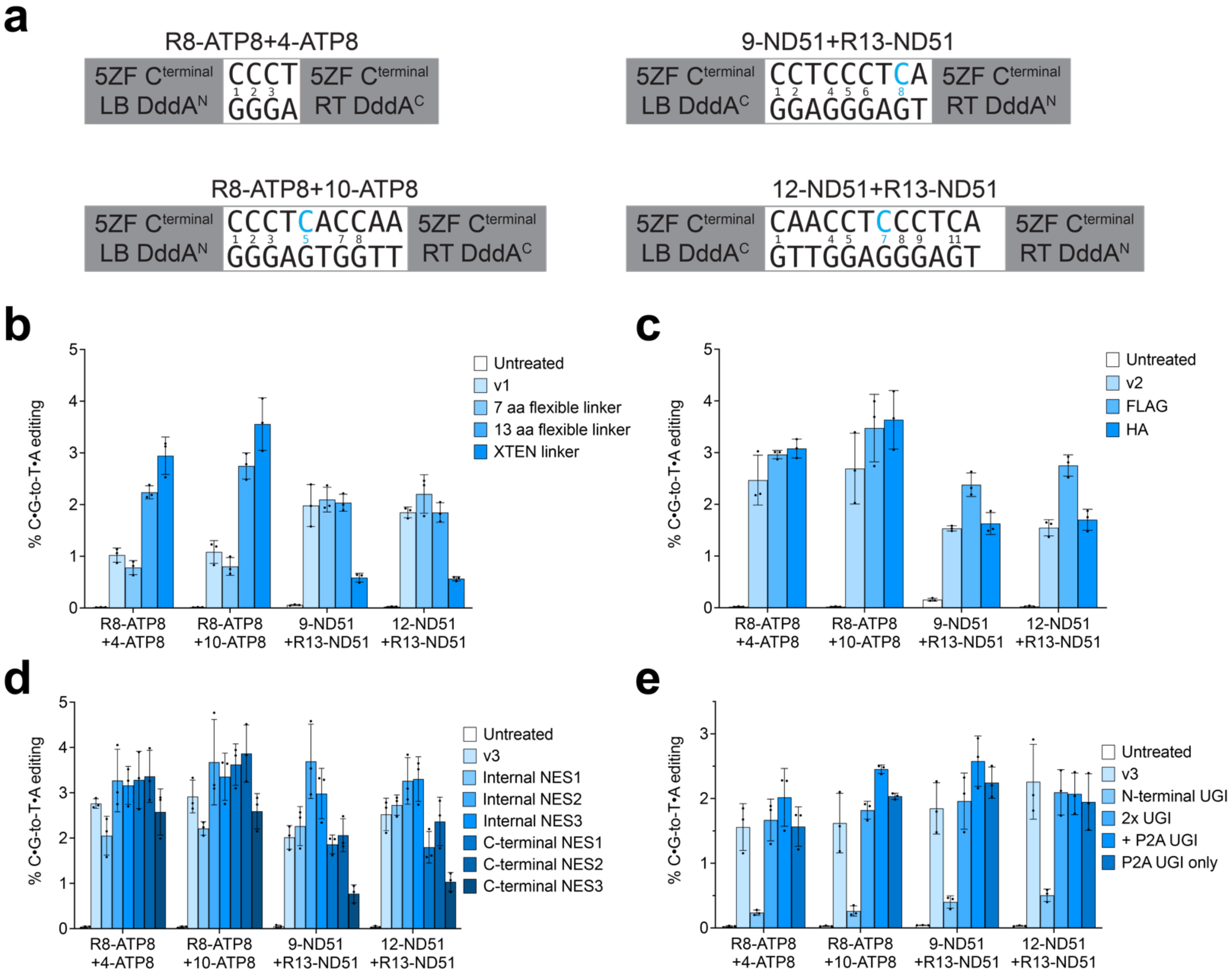

Supplementary Figure 2 | ZF-DdCBE architecture optimization. a, Initial mitochondrial ZF-DdCBE pairs used to establish v1 to v5 architectural improvements. For each site the DNA spacing region, split DddA orientation, ZF array lengths, architecture type (N- or C-terminal fusion of split DddA relative to the ZF array), and ZF-targeted DNA strands (LB=left bottom, RT=right top) are shown, and the cytosine with the highest editing efficiency is colored in blue. ZF-DdCBE naming convention follows A+B where A and B specify the left and right ZF, respectively. Nucleotide numbering starts with the first 5'-nucleotide in the spacing region designated position 1. For R8-ATP8+4-ATP8, nucleotide C5 has the highest editing efficiency. **b**-**e**, Mitochondrial DNA base editing efficiencies of HEK293T cells treated with four ZF-DdCBE pairs testing the effects of: (**b**) replacing the two-amino acid linker in architecture v1 with a 7- or 13-amino acid Gly/Ser-rich flexible linker, or a 32-amino acid XTEN linker; (**c**), inserting a FLAG or HA tag immediately downstream of the MTS in architecture v2; (**d**), adding an additional NES from HIV-1 Rev (NES1), MAPKK (NES2), or MVM NS2 (NES3) to architecture v3, either downstream of the existing internal NES or at the C-terminus of the protein; or (**e**), moving the location of UGI within the fusion protein to a position N-terminal of the 5ZF array, appending a

second copy of UGI to the C-terminus (2x UGI), or expressing a separate mitochondrially targeted UGI *in trans* using a self-cleaving P2A peptide (with (P2A UGI only) or without (+ P2A UGI) removing the C-terminally fused UGI) compared to architecture v3. Values and errors reflect the mean±s.d. of *n*=3 independent biological replicates. The editing efficiencies shown are for the most efficiently edited C•G within the spacing region. Source data are provided as a Source Data file.

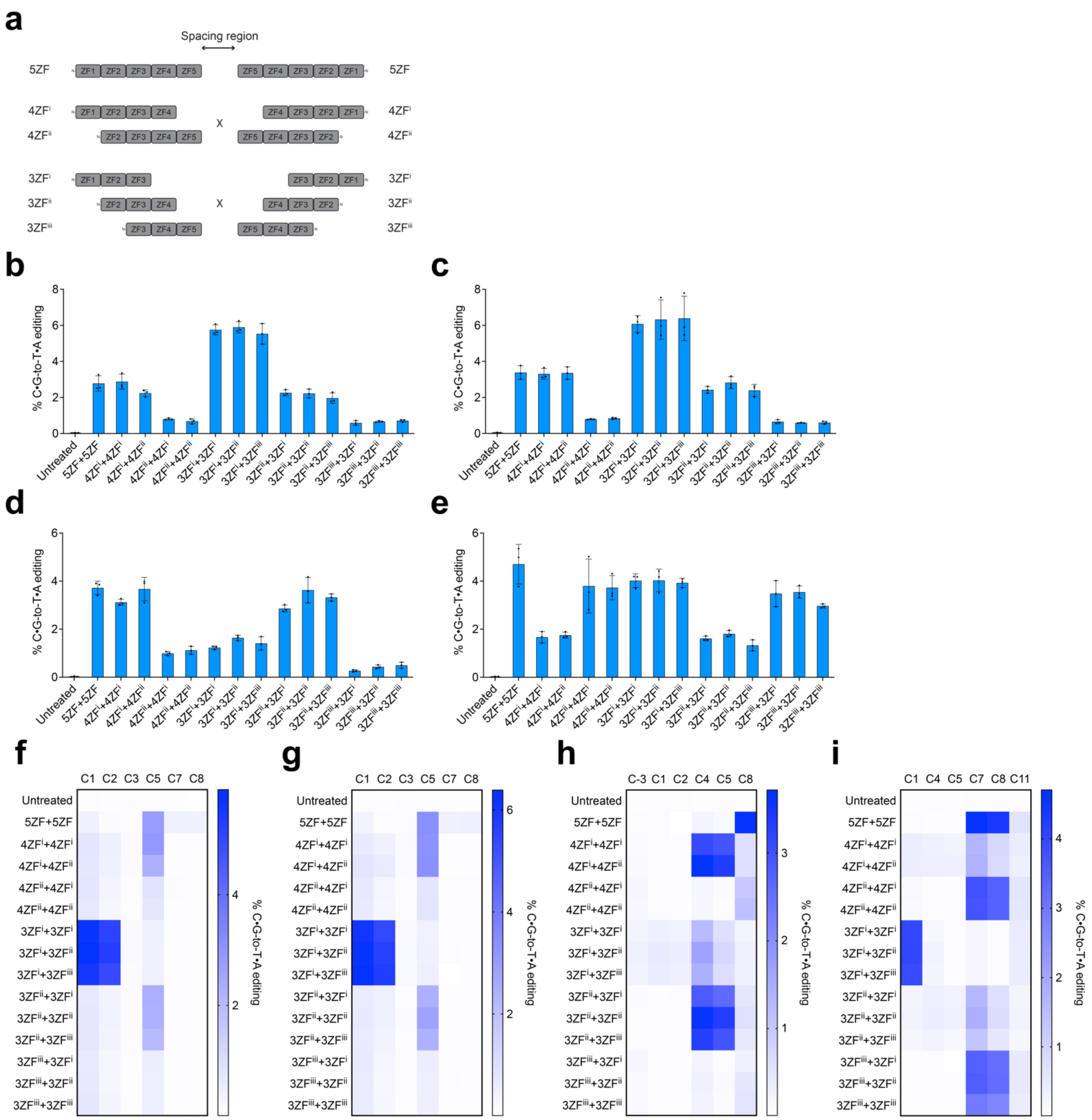

Supplementary Figure 3 | ZF array length and positioning influences ZF-DdCBE editing efficiency. a, Truncation of 5ZF arrays to create a set of two 4ZFs and a set of three 3ZFs by removing either one or two individual ZFs, respectively, creates four resulting 4ZF+4ZF combinations and nine 3ZF+3ZF combinations derived from the original 5ZF+5ZF ZF-DdCBE pair. **b-i**, Mitochondrial DNA base editing efficiencies of HEK293T cells treated with truncated v5 ZF-DdCBE pairs derived from (**b**, **f**) R8-ATP8+4-ATP8, (**c**, **g**) R8-ATP8+10-ATP8, (**d**, **h**) 9-ND51+R13-ND51, or (**e**, **i**) 12-ND51+R13-ND51. The editing efficiencies shown are for the most efficiently edited C•G within the spacing region. For **b-e**, values and errors reflect the mean±s.d. of *n*=3 independent biological replicates. For **f-i**, values reflect the mean of *n*=3 independent

biological replicates. Nucleotide numbering starts with the first 5'-nucleotide in the spacing region between the original 5ZF+5ZF pair designated position 1. Source data are provided as a Source Data file.

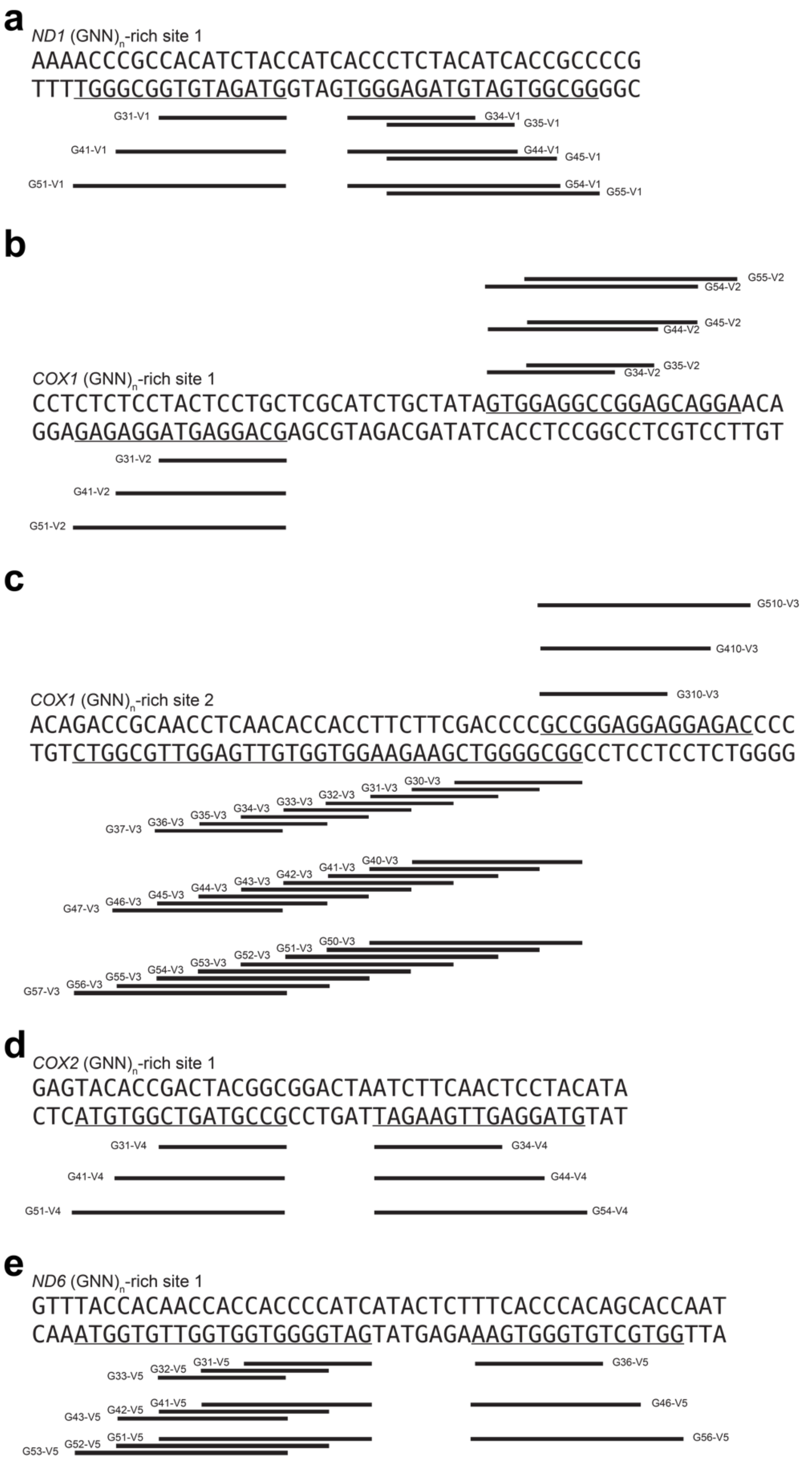

Supplementary Figure 4 | Design of ZF-DdCBEs at $(GNN)_n$-rich sites. Design of 3ZF, 4ZF and 5ZF arrays at (**a**) *ND1* $(GNN)_n$-rich site 1, (**b**) *COX1* $(GNN)_n$-rich site 1, (**c**) *COX1* $(GNN)_n$-rich site 2, (**d**) *COX2* $(GNN)_n$-rich site 1, and (**e**) *ND6* $(GNN)_n$-rich site 1. $(GNN)_n$ sequences are underlined, and ZF-targeted DNA sequences are indicated by thick black lines vertically above or below the corresponding DNA sequence.

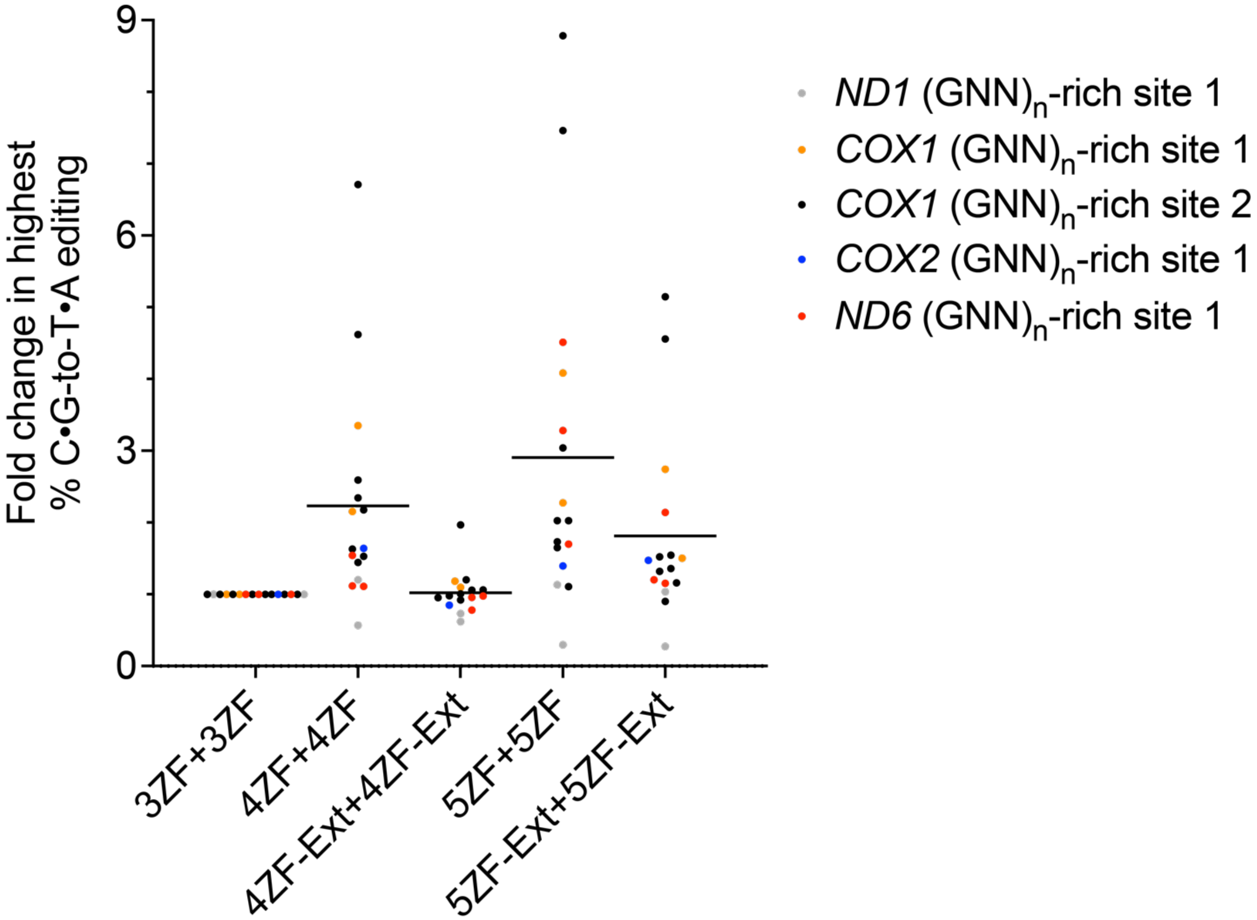

Supplementary Figure 5 | Extension of ZF array length improves ZF-DdCBE editing efficiency, but including extended linkers is detrimental. Mitochondrial DNA base editing efficiencies of HEK293T cells treated with 3ZF+3ZF, 4ZF+4ZF, or 5ZF+5ZF v5 ZF-DdCBE pairs targeting *ND1* $(GNN)_n$-rich site 1, *COX1* $(GNN)_n$-rich site 1 and 2, *COX2* $(GNN)_n$-rich site 1, and *ND6* $(GNN)_n$-rich site 1. To generate the ZF array length series, 3ZF arrays were extended outwards away from the spacing region to create longer 4ZF or 5ZF arrays, all of which share the same split DddA positioning and therefore maintained a fixed spacing region, as shown in Fig. S4. 4ZF-Ext+4ZF-Ext and 5ZF-Ext+5ZF-Ext reflect ZF-DdCBE pairs in which a single extended linker (TGSEKP) was incorporated into each ZF array, replacing the canonical linker (TGEKP) at that position. For N-terminal ZF-DdCBE architectures, the extended linker was inserted between ZF3 and ZF4 (the third and fourth ZF repeats) for both 4ZF-Ext and 5ZF-Ext arrays. For C-terminal ZF-DdCBE architectures, the extended linker was inserted between ZF1 and ZF2 for 4ZF-Ext arrays, or between ZF2 and ZF3 for 5ZF-Ext arrays. Values show the fold-change in editing efficiency compared to the corresponding 3ZF+3ZF pair, for the most efficiently edited C•G within the spacing region. Values reflect the mean±s.d. of n=3 independent biological replicates. The global mean for each data group of n=16 ZF-DdCBE pairs is shown as a horizontal black line. Source data are provided as a Source Data file.

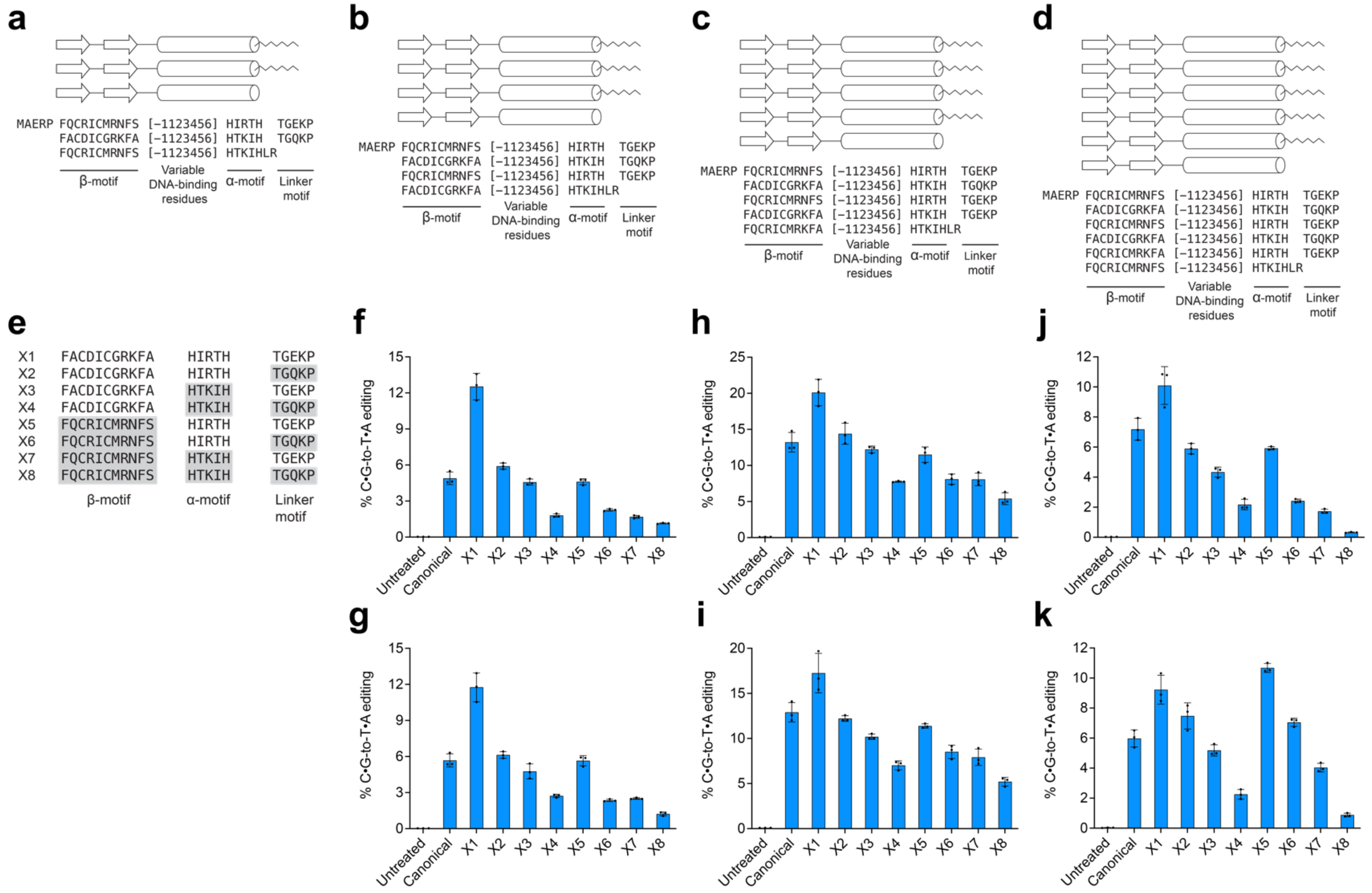

Supplementary Figure 6 | Defining new ZF scaffolds improves ZF-DdCBE editing efficiency. a-**d**, Secondary structure and amino acid sequence of canonical (**a**) 3ZF, (**b**) 4ZF, (**c**) 5ZF, and (**d**) 6ZF arrays. **e**, Amino acid sequences of ZF scaffolds X1 to X8. Different beta-motif, alpha-motif, and linker-motif sequences are colored in grey. **f**-**k**, Mitochondrial DNA base editing efficiencies of HEK293T cells treated with v5 ZF-DdCBE pairs (**f**) R8-ATP8+4-ATP8, (**g**) R8-ATP8+10-ATP8, (**h**) R8-3i-ATP8+4-3i-ATP8, (**i**) R8-3i-ATP8+10-3ii-ATP8, (**j**) 9-ND51+R13-ND51, or (**k**) 12-ND51+R13-ND51 with either canonical ZF scaffold or ZF scaffolds X1 to X8. For **f**-**k**, values and errors reflect the mean±s.d. of *n*=3 independent biological replicates. The editing efficiencies shown are for the most efficiently edited C•G within the spacing region. Source data are provided as a Source Data file.

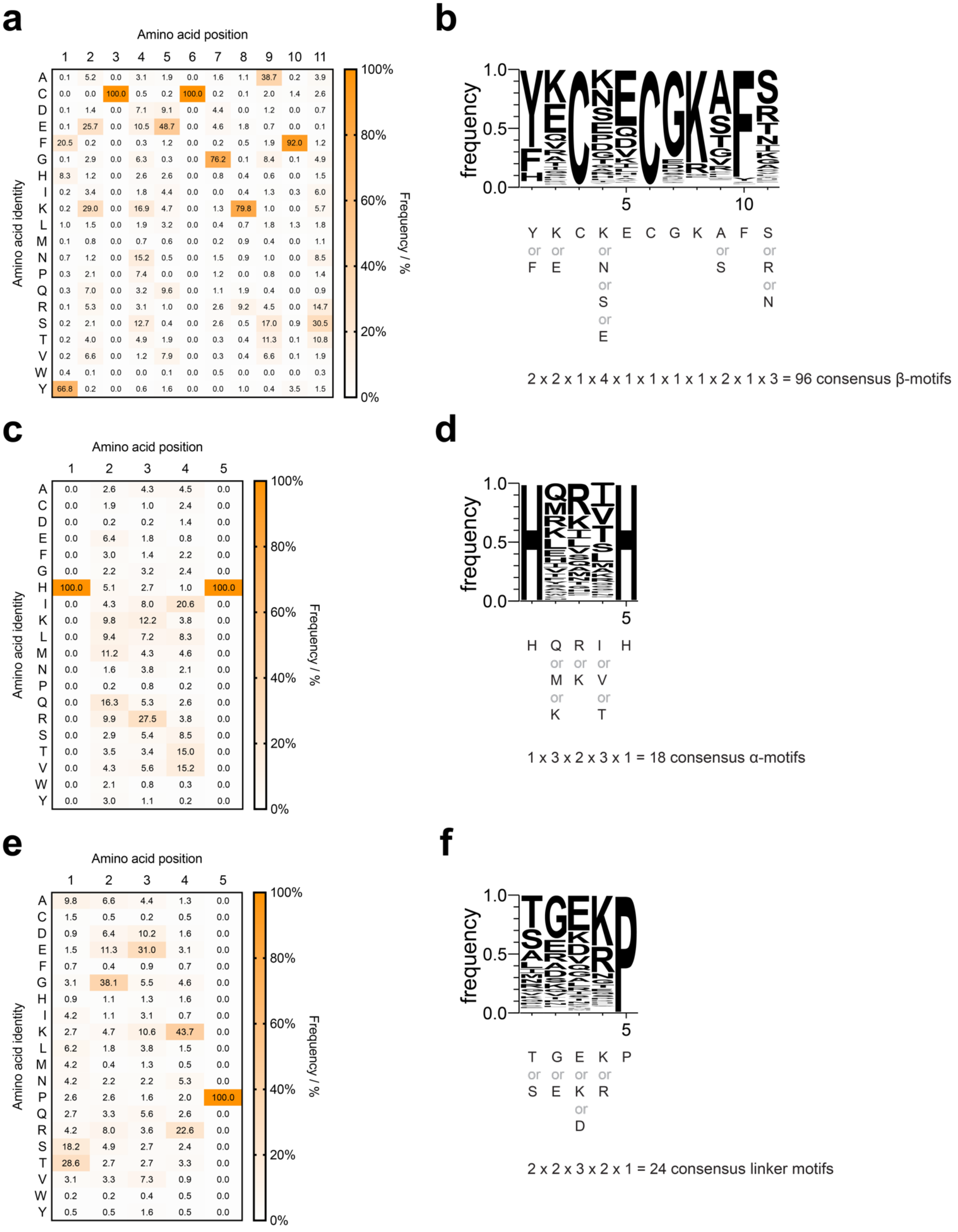

Supplementary Figure 7 | Defining new ZF scaffolds derived from the human proteome. a, **c**, **e**, Amino acid frequencies at each sequence position from (**a**) 3,356 unique beta-motifs, (**c**)

625 unique alpha-motifs, and (**e**) 549 unique linker motifs in the human proteome. **b**, **d**, **f**, Amino acid frequencies at each sequence position displayed as a sequence logo (top) used to define (**b**) consensus beta-motif, (**d**) consensus alpha-motif, and (**f**) consensus linker motif sequences by applying a 10% frequency cut-off at each sequence position (bottom).

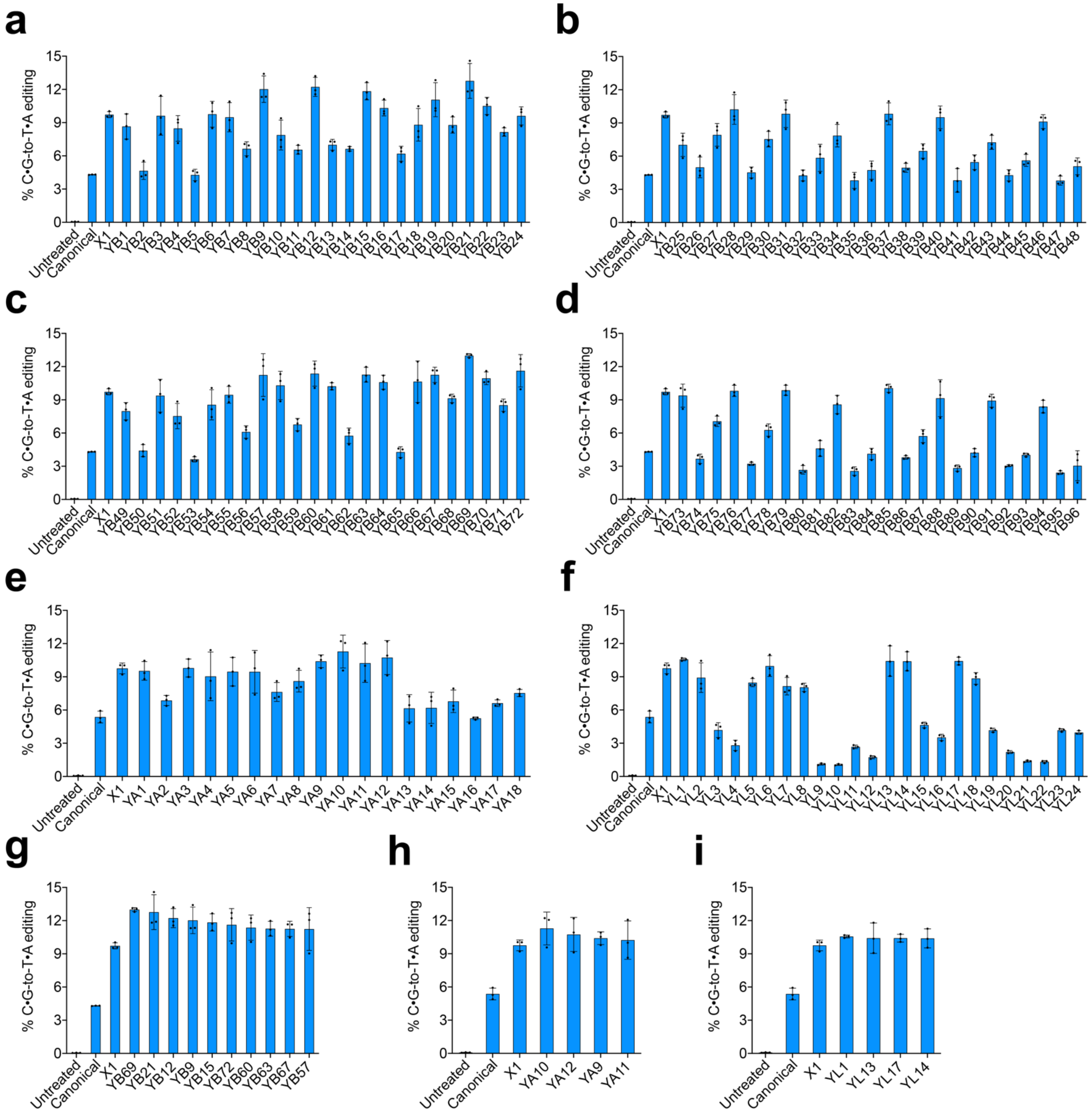

Supplementary Figure 8 | Identifying new ZF scaffolds derived from the human proteome that improve ZF-DdCBE editing efficiency. a-f, Mitochondrial DNA base editing efficiencies of HEK293T cells treated with v5 ZF-DdCBE pair R8-ATP8+4-ATP8 with either canonical or X1 ZF scaffolds, or ZF scaffolds containing (**a**) consensus beta-motifs YB1 to YB24, (**b**) YB25 to YB48, (**c**) YB49 to YB72, (**d**) YB73 to YB96, (**e**) consensus alpha-motifs YA1 to YA18, or (**f**) consensus linker motifs YL1 to YL24. **g-i**, The editing efficiencies of (**g**) the ten top-performing consensus beta-motifs, (**h**) four top-performing consensus alpha-motifs, or (**i**) four top-performing linker motifs. For **a**-**i**, values and errors reflect the mean±s.d. of *n*=3 independent biological replicates.

The editing efficiencies shown are for the most efficiently edited C•G within the spacing region. Source data are provided as a Source Data file.

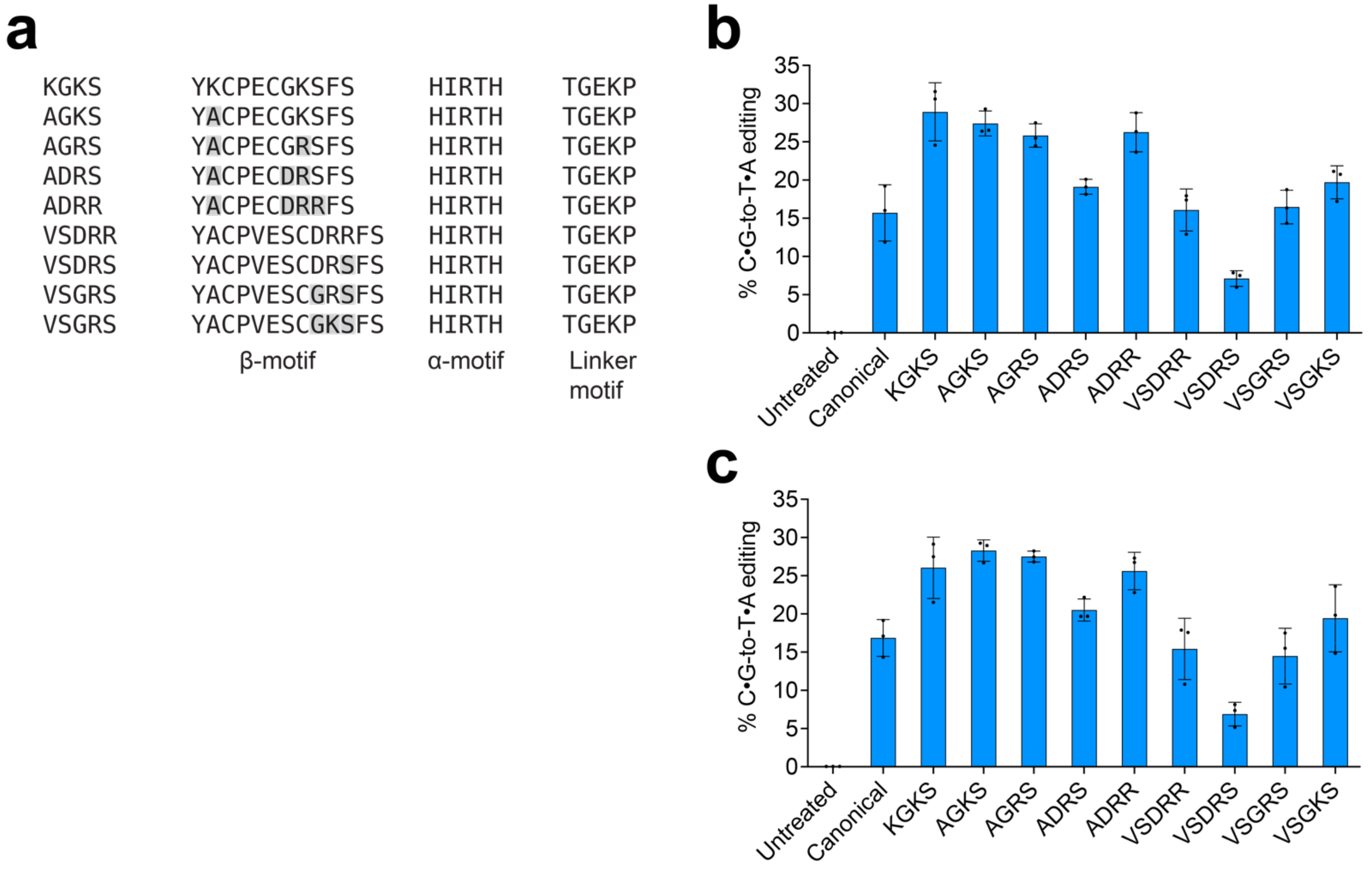

Supplementary Figure 9 | Identifying new ZF scaffolds derived from ZFN268(F1) and Sp1C that improve ZF-DdCBE editing efficiency. a, Amino acid sequences of ZF scaffolds based on ZF scaffold X1 and containing beta-motifs derived from *ZFN268*(F1) and Sp1C sequences. Amino acid changes are colored in grey. **b**-**c**, Mitochondrial DNA base editing efficiencies of HEK293T cells treated with (**b**) v5 ZF-DdCBE pairs R8-3i-ATP8+4-3i-ATP8, or (**c**) R8-3i-ATP8+10-3ii-ATP8 with either canonical ZF scaffold or ZF scaffolds from KGKS to VSGRS. For **b** and **c**, values and errors reflect the mean±s.d. of *n*=3 independent biological replicates. The editing efficiencies shown are for the most efficiently edited C•G within the spacing region. Source data are provided as a Source Data file.

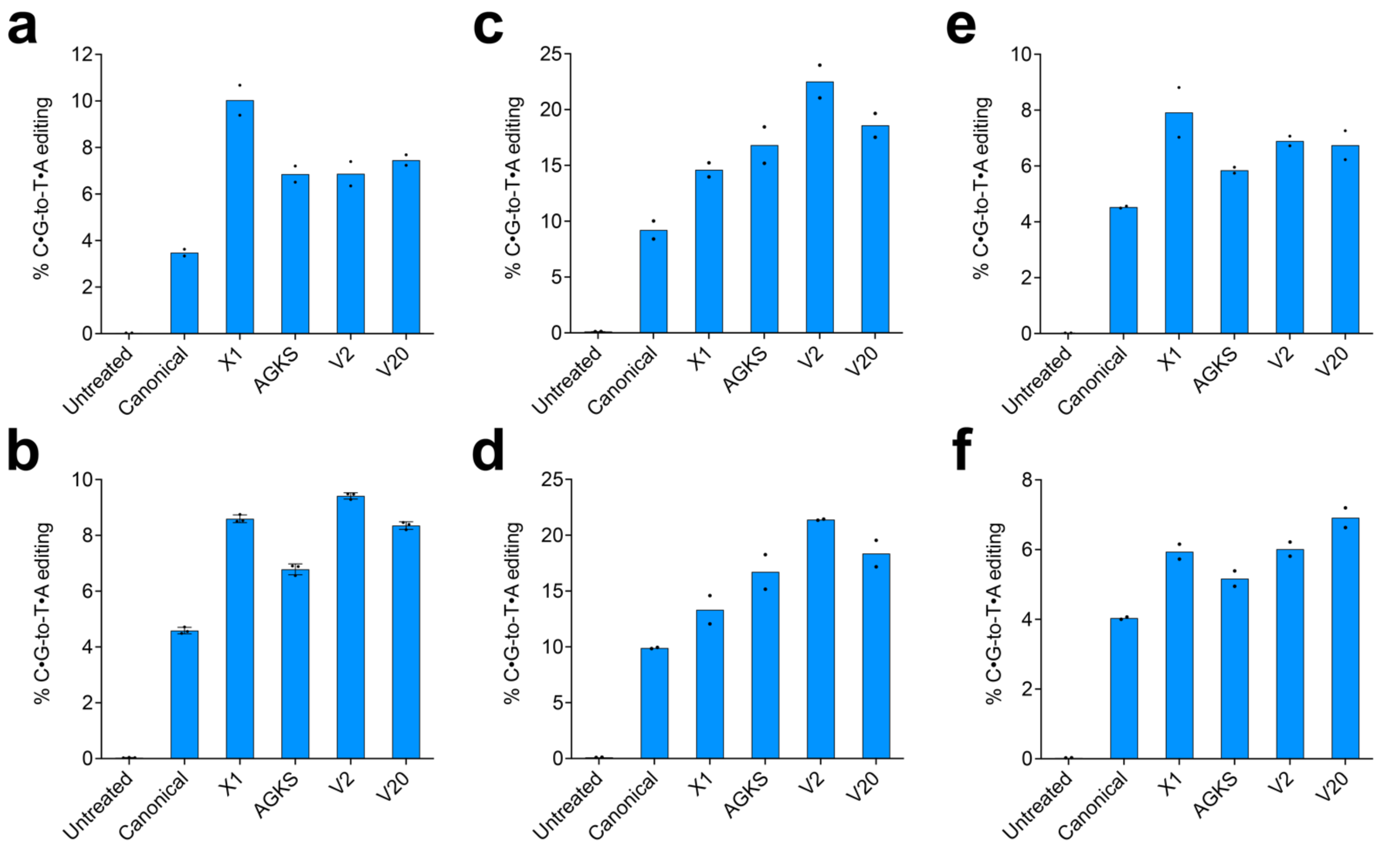

Supplementary Figure 10 | Optimized ZF scaffolds increase ZF-DdCBE editing efficiency. **a**-**f**, Mitochondrial DNA base editing efficiencies of HEK293T cells treated with (**a**) v5 ZF-DdCBE pairs R8-ATP8+4-ATP8, (**b**) R8-ATP8+10-ATP8, (**c**) R8-3i-ATP8+4-3i-ATP8, (**d**) R8-3i-ATP8+10-3ii-ATP8, (**e**) 9-ND51+R13-ND51, or (**f**) 12-ND51+R13-ND51 with either canonical or optimized ZF scaffolds. For **a** and **c**-**f**, values reflect the mean of *n*=2 independent biological replicates. For **b**, values and errors reflect the mean±s.d. of *n*=3 independent biological replicates. The editing efficiencies shown are for the most efficiently edited C•G within the spacing region. Source data are provided as a Source Data file.

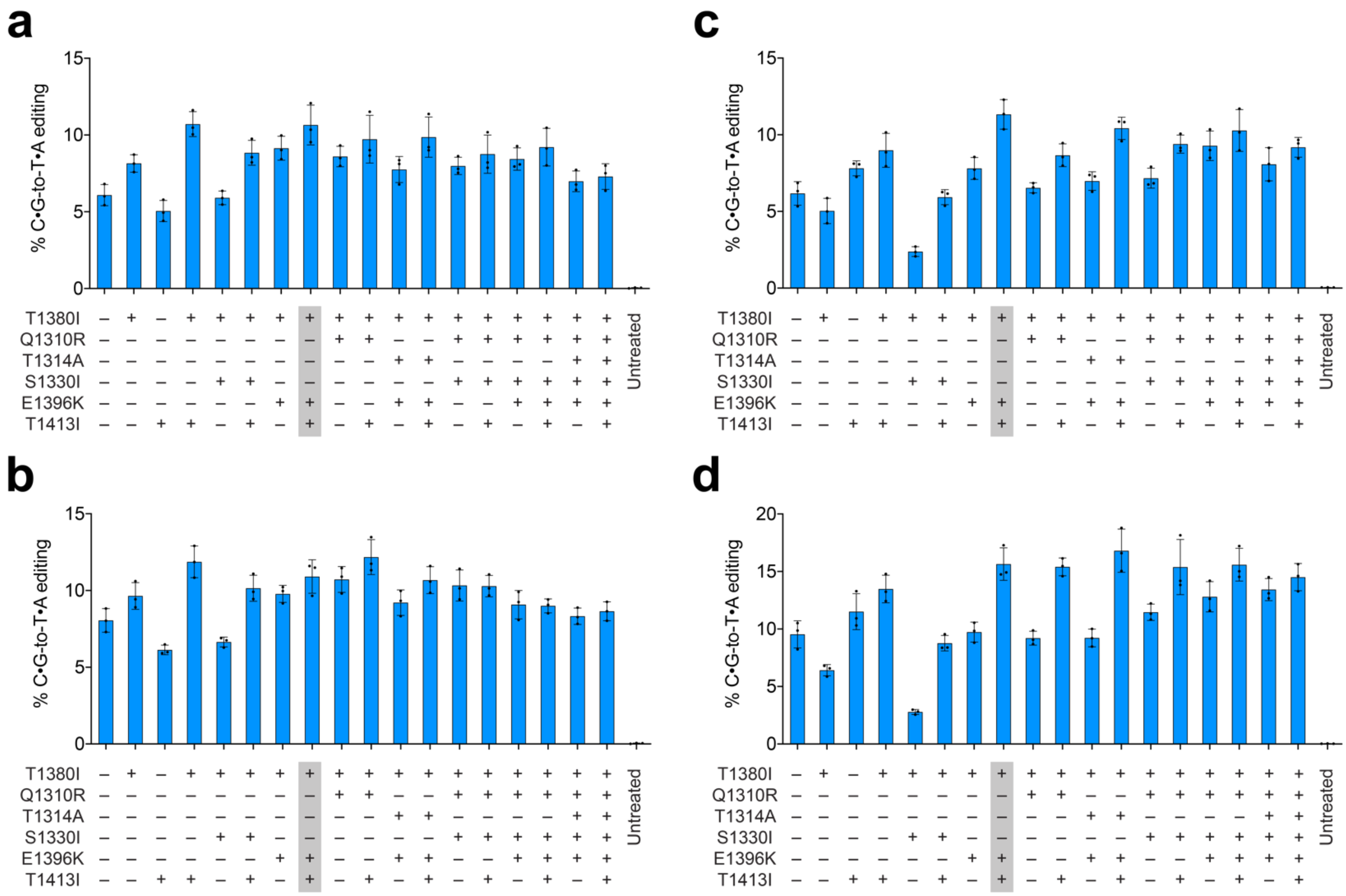

Supplementary Figure 11 | DddA mutations enhance ZF-DdCBE editing efficiency. a-d, Mitochondrial DNA base editing efficiencies of HEK293T cells treated with v5 ZF-DdCBE pairs (**a**) R8-ATP8+4-ATP8, (**b**) R8-ATP8+10-ATP8, (**c**) 9-ND51+R13-ND51, or (**d**) 12-ND51+R13-ND51 containing combinations of mutations in $DddA^N$ and $DddA^C$. The triple mutant T1380I, E1396K, T1413I is colored in grey. For **a-d**, values and errors reflect the mean±s.d. of *n*=3 independent biological replicates. The editing efficiencies shown are for the most efficiently edited C•G within the spacing region. Source data are provided as a Source Data file.

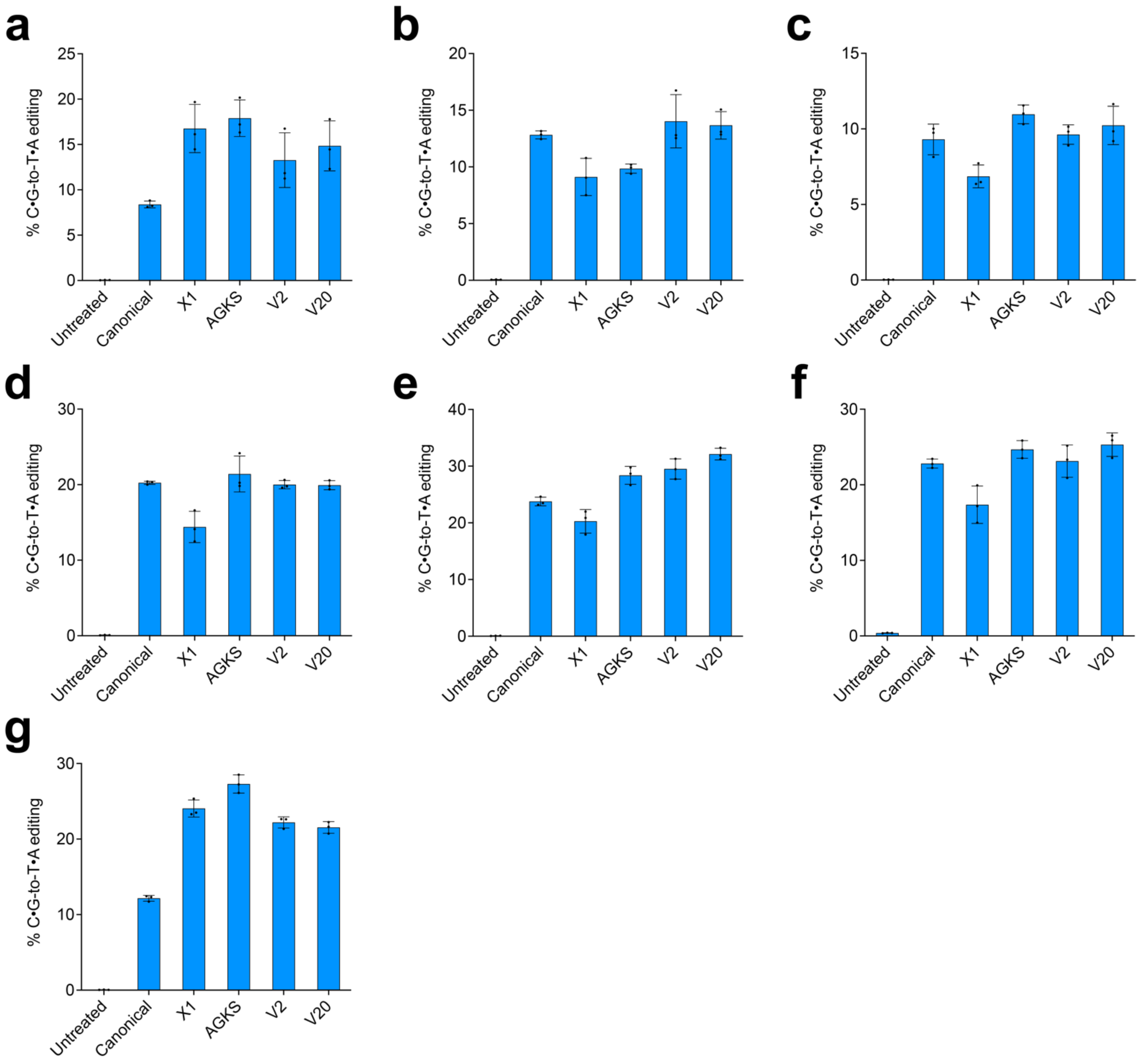

Supplementary Figure 12 | Optimized ZF scaffolds increase ZF-DdCBE editing efficiency. **a**-**g**, Mitochondrial DNA base editing efficiencies of HEK293T cells treated with v5 ZF-DdCBE pairs (**a**) G24-R1b+G32-R1b, (**b**) G22-R13+G24-R13, (**c**) G32-R6a+G21-R6a, (**d**) G36-R6c+G212-R6c, (**e**) G33-V1+G35-V1, (**f**) G22-V2+G34-V2, or (**g**) G33-V5+G36-V5 with either canonical or optimized ZF scaffolds. For **a**-**g**, values and errors reflect the mean±s.d. of n=3 independent biological replicates. The editing efficiencies shown are for the most efficiently edited C•G within the spacing region. Source data are provided as a Source Data file.

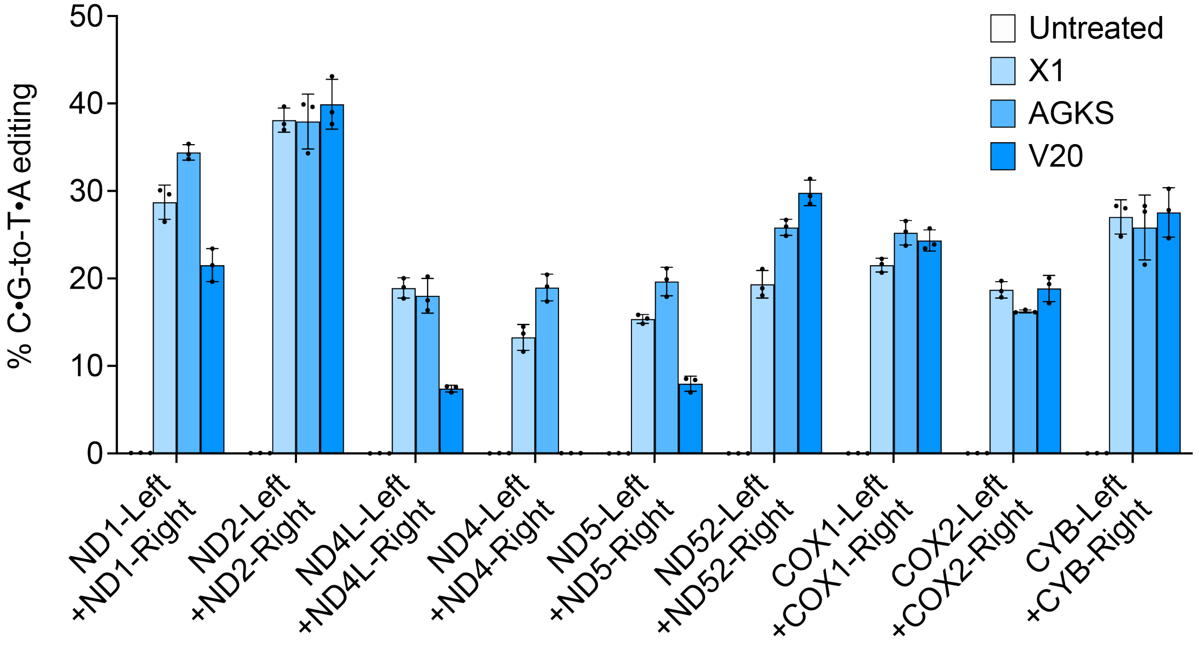

Supplementary Figure 13 | Identifying ZF scaffolds that support the highest editing efficiency for ZFD-derived ZF-DdCBEs. Mitochondrial DNA base editing efficiencies of HEK293T cells treated with v7 ZF-DdCBE pairs ND1-Left+ND1-Right, ND2-Left+ND2-Right, ND4L-Left+ND4L-Right, ND4-Left+ND4-Right, ND5-Left+ND5-Right, ND52-Left+ND52-Right, COX1-Left+COX1-Right, COX2-Left+COX2-Right, or CYB-Left+CYB-Right with the indicated optimized ZF scaffolds. Values and errors reflect the mean±s.d. of *n*=3 independent biological replicates. The editing efficiencies shown are for the most efficiently edited C•G within the spacing region. Source data are provided as a Source Data file.

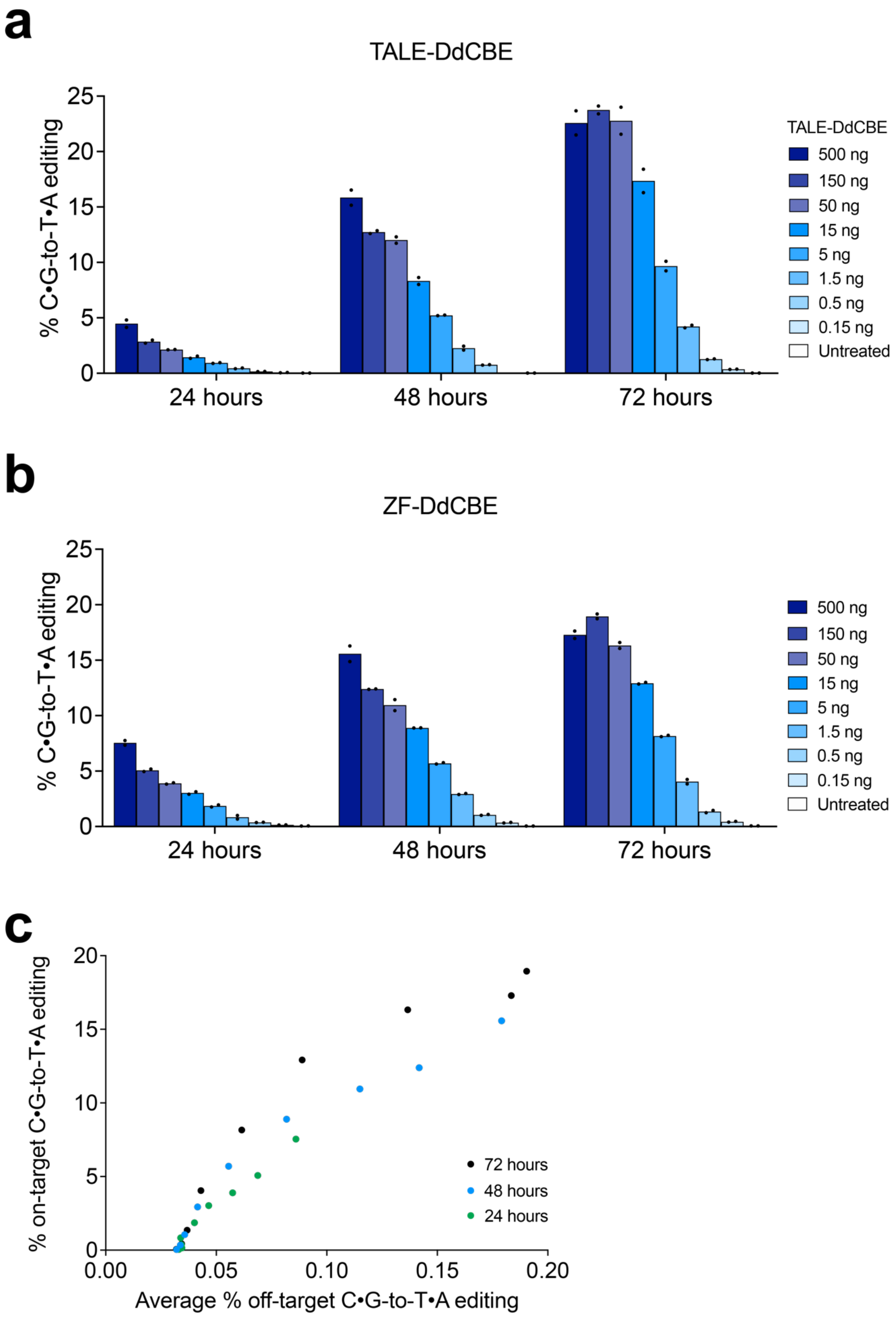

Supplementary Figure 14 | Time course of TALE-DdCBE and ZF-DdCBE editing efficiencies over time. a-b, Mitochondrial DNA base editing efficiencies of HEK293T cells

treated with (**a**) TALE-DdCBE pair ND4-DdCBE, or (**b**) v5 ZF-DdCBE pair R8-3i-ATP8+4-3i-ATP8 using the indicated amount of plasmid DNA. Cells were lysed after the indicated time period. **c**, On-target and average off-target editing efficiencies within amplicon ATP8 of HEK293T cells treated with v5 ZF-DdCBE pair R8-3i-ATP8+4-3i-ATP8 using 500, 150, 50, 15, 5, 1.5, 0.5, or 0.15 ng plasmid DNA, or untreated HEK293T cells. Cells were lysed after the indicated time period. For **a**-**b**, values reflect the mean of *n*=2 independent biological replicates. The editing efficiencies shown are for the most efficiently edited C•G within the spacing region. For **c**, values reflect the mean of *n*=2 independent biological replicates. The on-target editing efficiencies shown are for the most efficiently edited C•G within the spacing region. Source data are provided as a Source Data file.

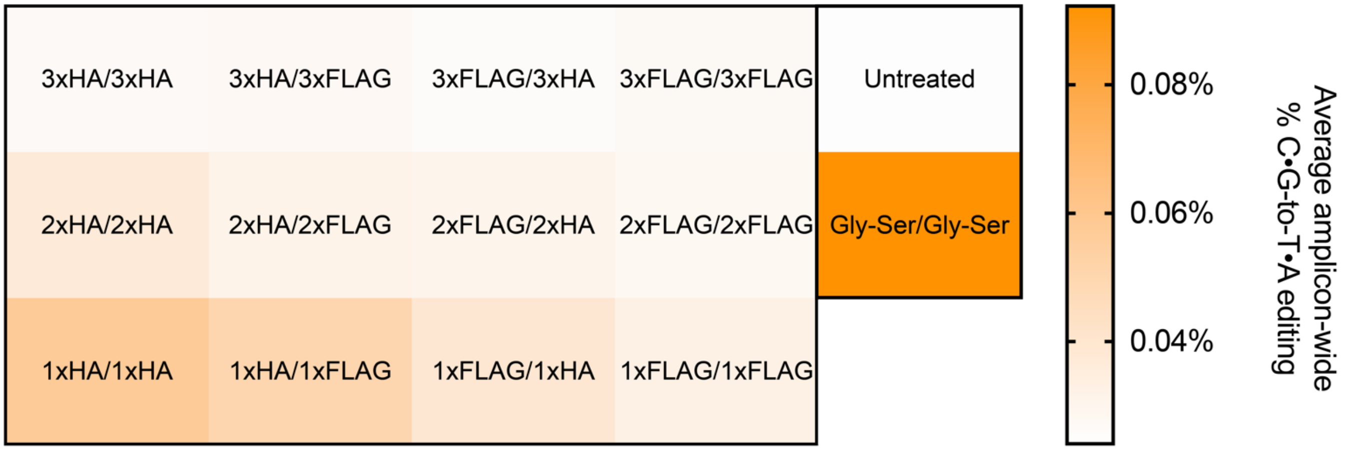

Supplementary Figure 15 | Amino acid sequences immediately upstream of DddA^{N} and DddA^{C} influence non-targeted editing activity. Average non-targeted editing efficiencies within amplicon ATP8 of HEK293T cells treated with DddA^{N}-UGI and DddA^{C}-UGI preceded by the indicated sequences. Naming convention follows A/B, where A and B correspond to the amino acid sequences immediately upstream of DddA^{N} and DddA^{C}, respectively. Values reflect the mean of *n*=3 independent biological replicates. Source data are provided as a Source Data file.

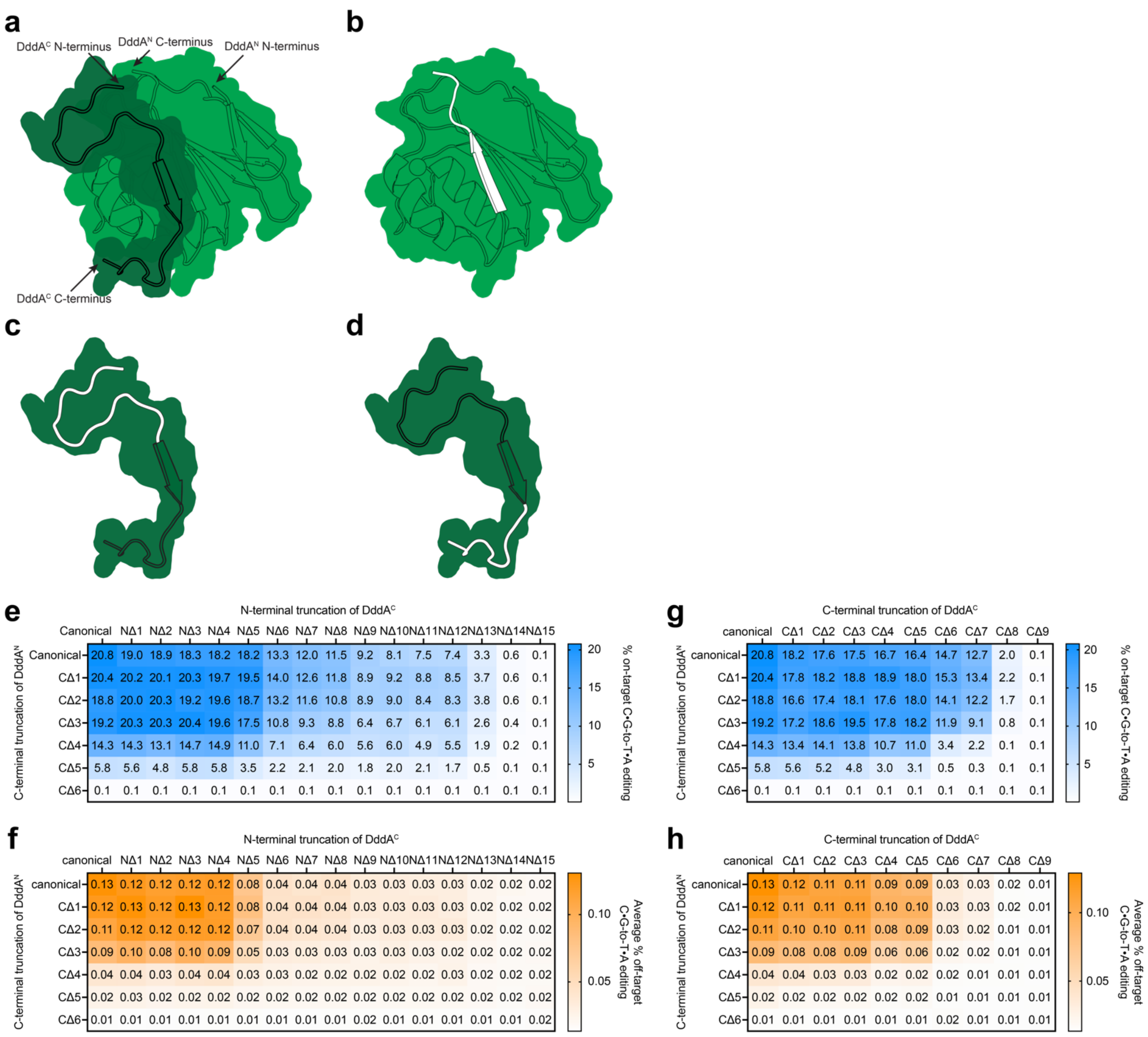

Supplementary Figure 16 | DddA truncation reduces ZF-DdCBE off-target editing.
a, Crystal structure of DddA (PDB 6U08) complexed with DddI, the natural protein inhibitor of DddA (not shown). $DddA^N$ and $DddA^C$ are colored in light green and dark green, respectively, and have N- and C-termini indicated. **b**-**d**, (**b**) C-terminal truncation of $DddA^N$, (**c**) N-terminal truncation of $DddA^C$, and (**d**) C-terminal truncation of $DddA^C$ are shown with residues incrementally removed colored in white. **e**-**h**, (**e** and **g**) On-target and (**f** and **h**) average off-target editing efficiencies within amplicon ATP8 of HEK293T cells treated with canonical v7 ZF-DdCBE pair R8-3i-ATP8+4-3i-ATP8 or variants containing $DddA^N$ and $DddA^C$ truncations. For **e**-**h**, values reflect the mean of n=3 independent biological replicates. The on-target editing efficiencies shown are for the most efficiently edited C•G within the spacing region. Source data are provided as a Source Data file.

a

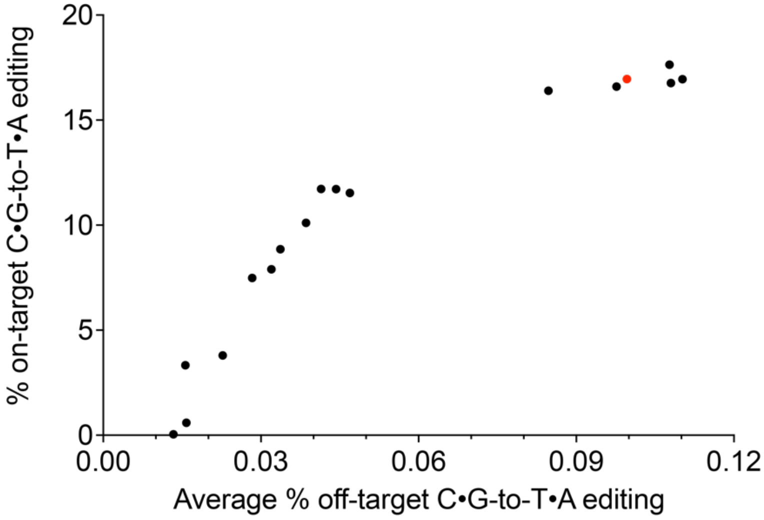

b

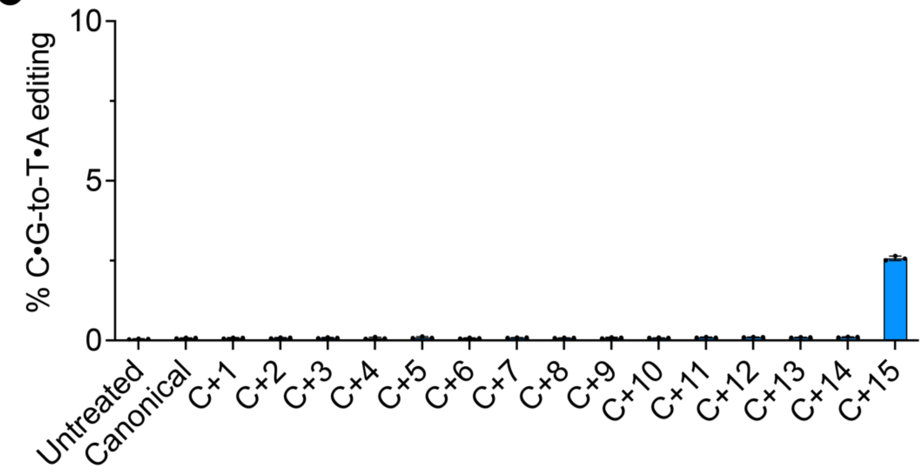

Supplementary Figure 17 | Shifting the position of the canonical G1397 split site within DddA. **a**, On-target and average off-target editing efficiencies within amplicon ATP8 of HEK293T cells treated with canonical v7 ZF-DdCBE pair R8-3i-ATP8+4-3i-ATP8 (colored in red) or variants containing C-terminally extended $DddA^N$ and N-terminally truncated $DddA^C$. **b**, Mitochondrial DNA base editing efficiencies of HEK293T cells treated with only a single ZF-DdCBE half (R8-3i-ATP8 from ZF-DdCBE pair R8-3i-ATP8+4-3i-ATP8) carrying canonical $DddA^N$ or C-terminally extended $DddA^N$ variants. Naming convention C+X signifies $\mathrm{DddA}^{\mathrm{N}}_{\mathrm{C+X}}$. For **a**, values reflect the mean of *n*=3 independent biological replicates. For **b**, values and errors reflect the mean±s.d. of *n*=3 independent biological replicates. The editing efficiencies shown are for the most efficiently edited C•G within the spacing region. Source data are provided as a Source Data file.

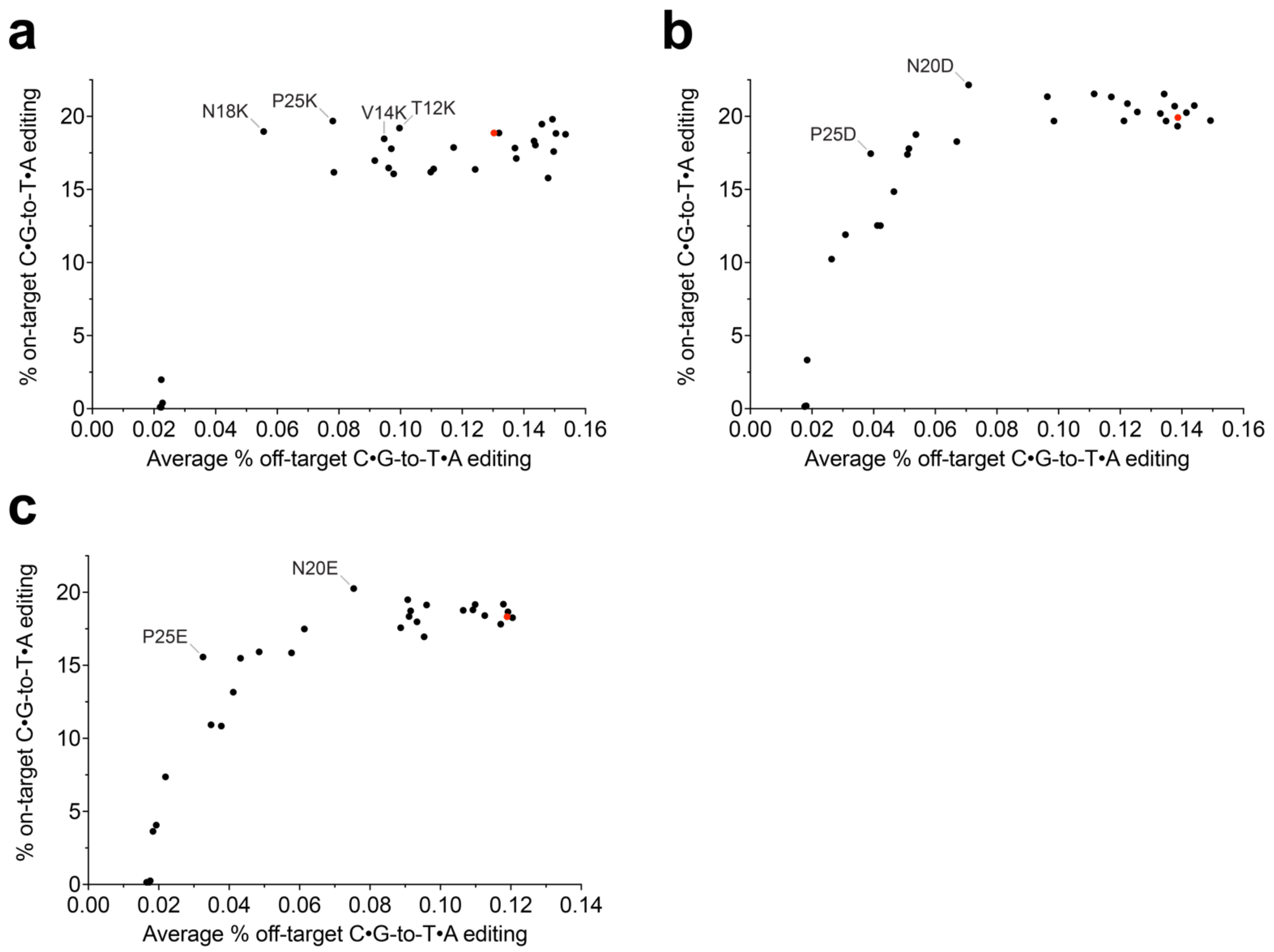

Supplementary Figure 18 | Introducing point mutations within DddA^{C}. a-**c**, On-target and average off-target editing efficiencies within amplicon ATP8 of HEK293T cells treated with canonical v7 ZF-DdCBE pair R8-3i-ATP8+4-3i-ATP8 (colored in red) or variants containing (**a**) Lys, (**b**) Asp, or (**c**) Glu point mutations within DddA^{C}. Values reflect the mean of *n*=3 independent biological replicates. The editing efficiencies shown are for the most efficiently edited C•G within the spacing region. Source data are provided as a Source Data file.

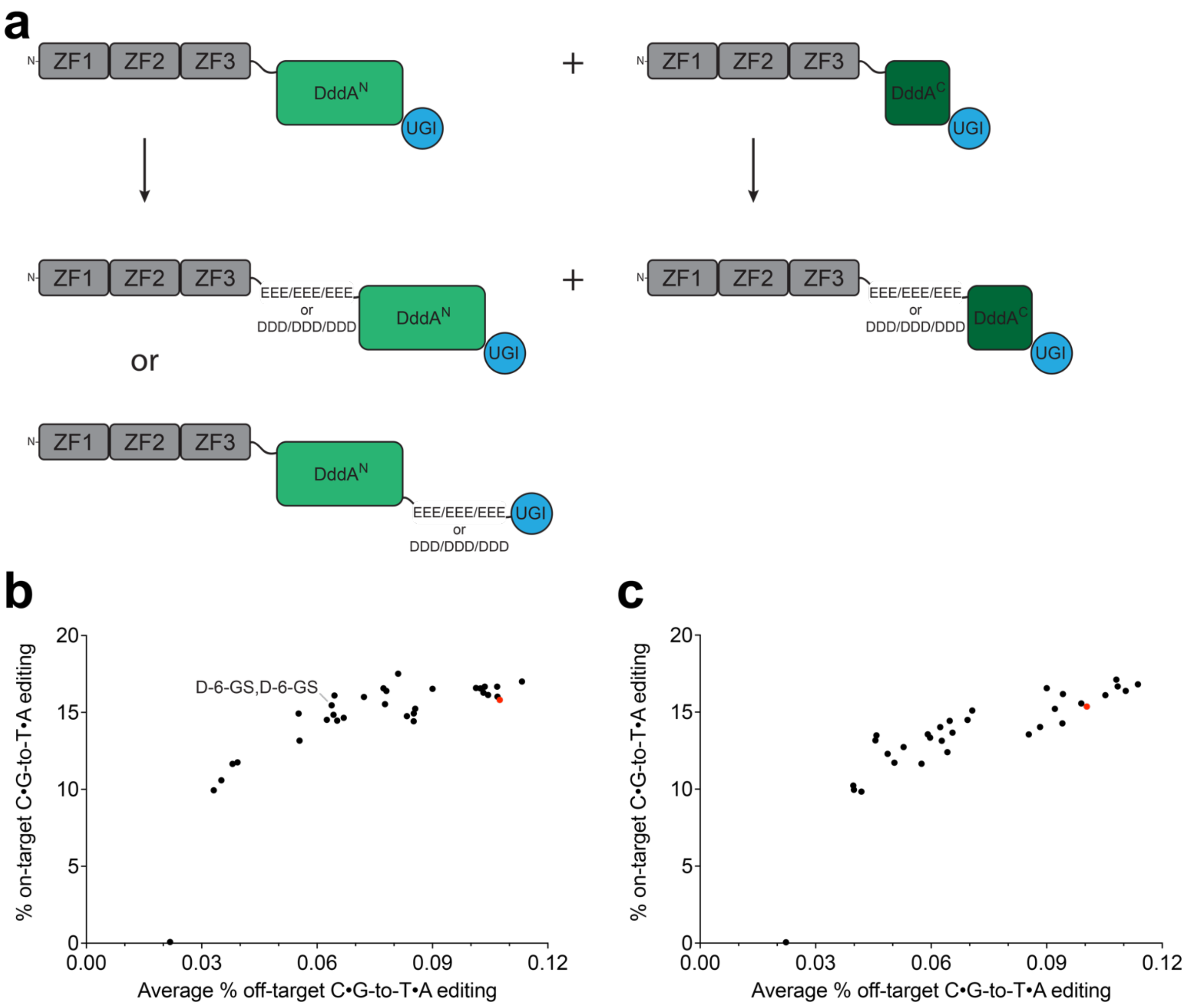

Supplementary Figure 19 | Introducing negative charge at the termini of DddA. Architectures of canonical ZF-DdCBEs and ZF-DdCBE variants containing a ZF array, Gly/Ser-rich flexible linker, split DddA deaminase, and UGI (N-terminal mitochondrial targeting signal, FLAG tag, and nuclear export signals are not shown). **a**, ZF-DdCBE variants are shown in which three, six, or nine residues in the 13-amino acid Gly/Ser-rich flexible linker upstream of DddA^{N} and DddA^{C} were mutated to either Glu (E) or Asp (D) residues. ZF-DdCBE variants are also shown in which three, six or nine Glu (E) or Asp (D) residues were inserted into the Gly/Ser-rich flexible linker downstream of DddA^{N}. **b-c**, On-target and average off-target editing efficiencies within amplicon ATP8 of HEK293T cells treated with canonical v7 ZF-DdCBE pair R8-3i-ATP8+4-3i-ATP8 (colored in red) or variants containing (**b**) Asp or (**c**) Glu residues upstream or downstream of DddA^{N} and DddA^{C}. Values reflect the mean of *n*=3 independent biological replicates. The editing efficiencies shown are for the most efficiently edited C•G within the spacing region. Source data are provided as a Source Data file.

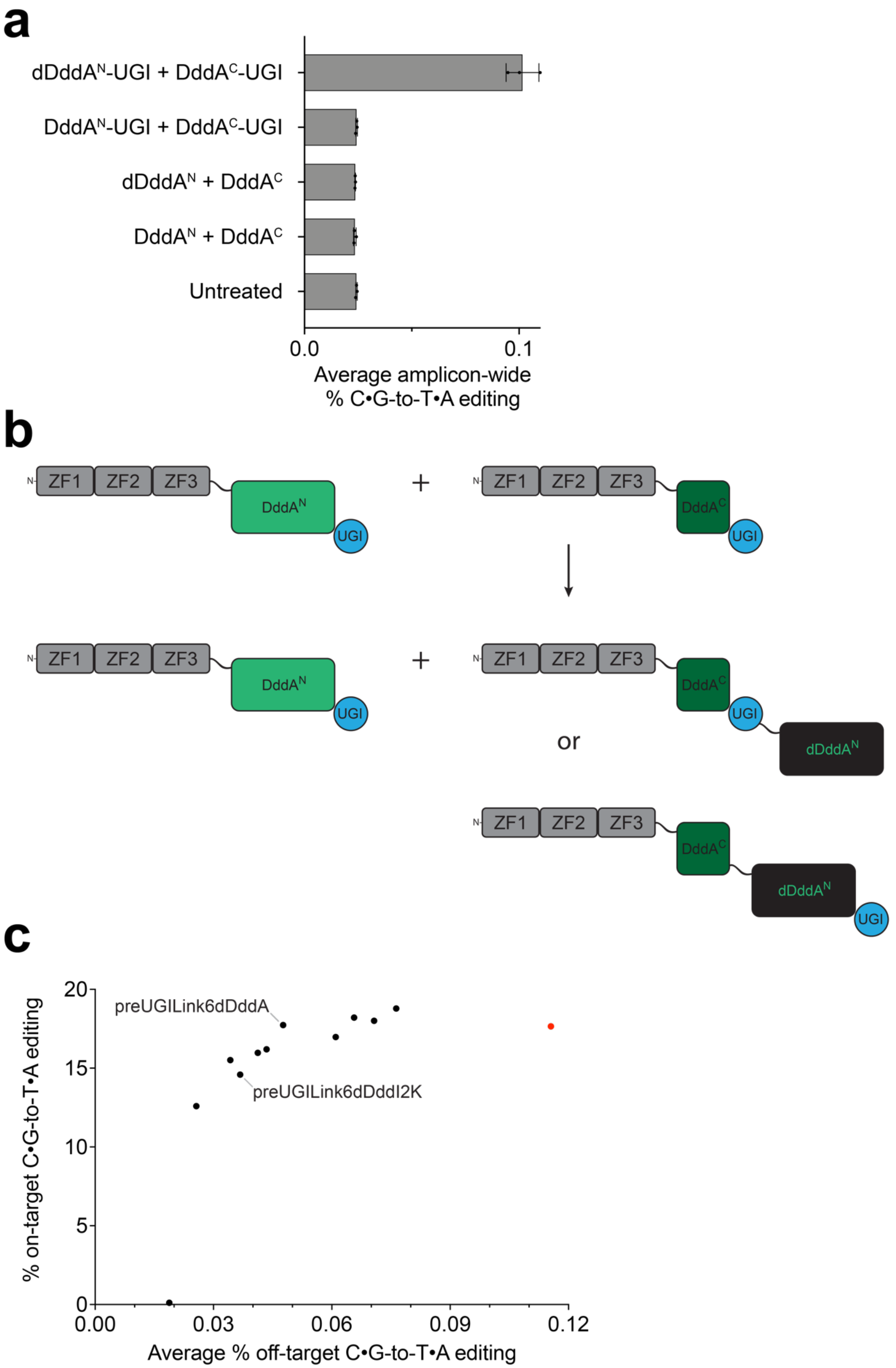

Supplementary Figure 20 | Capping with catalytically inactivated DddA^{N}. Architectures of canonical ZF-DdCBEs and ZF-DdCBE variants containing a ZF array, Gly/Ser-rich flexible

linker, split DddA deaminase, and UGI (N-terminal mitochondrial targeting signal, FLAG tag, and nuclear export signals are not shown). **a**, ZF-DdCBE variants are shown in which dDddA^{N} was fused downstream of DddA^{C} using Gly/Ser-rich flexible linkers, either before or after the UGI domain. **b**, Off-target editing efficiencies within mitochondrial off-target amplicon ATP8 of HEK293T cells treated with individual components of the v7 ZF-DdCBE architecture, with or without the DddA catalytically inactivating E1347A mutation. **c**, On-target and average off-target editing efficiencies within amplicon ATP8 of HEK293T cells treated with canonical v7 ZF-DdCBE pair R8-3i-ATP8+4-3i-ATP8 (colored in red) or variants containing fused catalytically inactivated DddA^{N}. For **b**, values and errors reflect the mean±s.d. of *n*=3 independent biological replicates. For **c**, values reflect the mean of *n*=3 independent biological replicates. The editing efficiencies shown are for the most efficiently edited C•G within the spacing region. Source data are provided as a Source Data file.

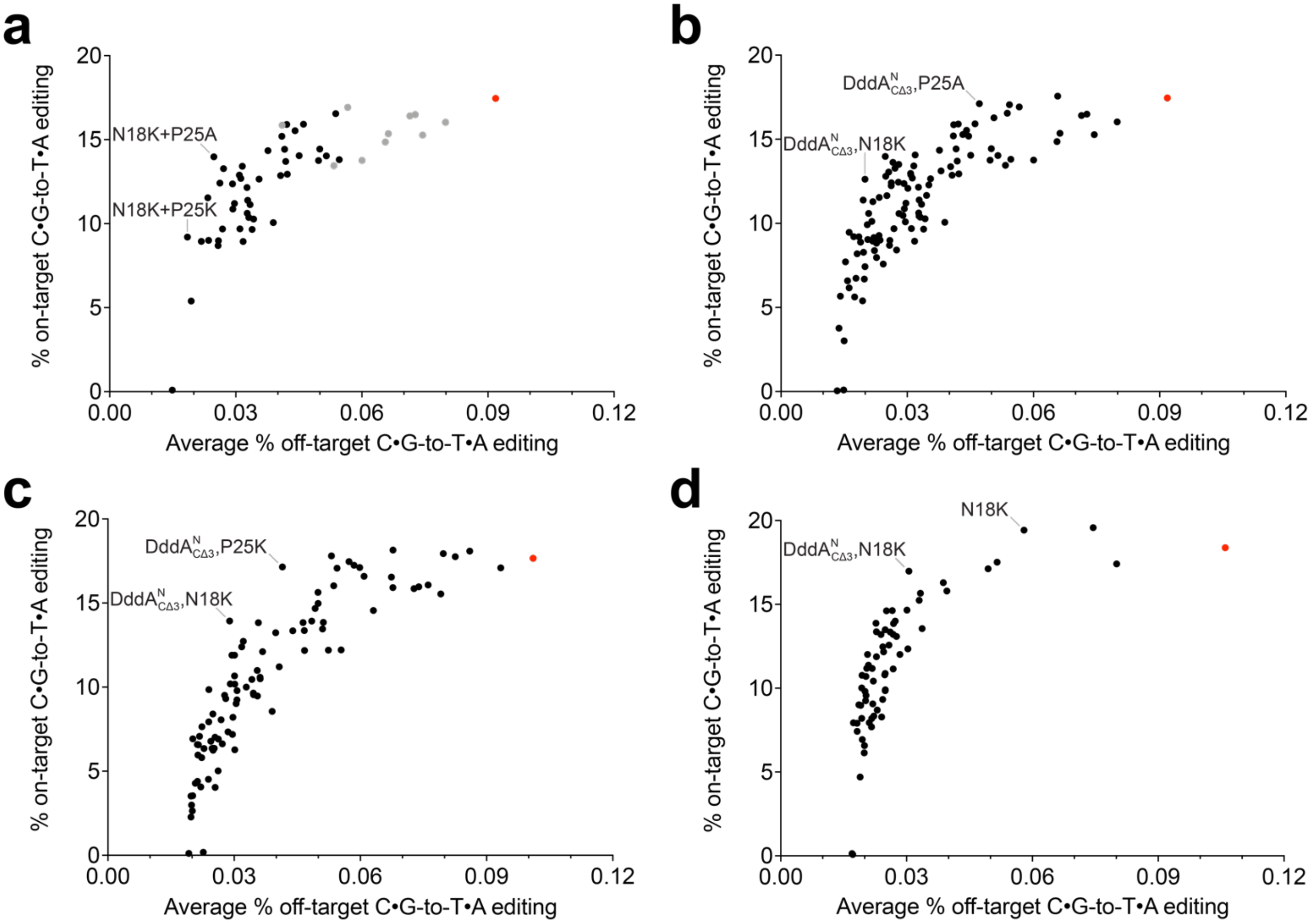

Supplementary Figure 21 | Combining approaches to reduce ZF-DdCBE off-target editing. **a**, On-target and average off-target editing efficiencies within amplicon ATP8 of HEK293T cells treated with canonical v7 ZF-DdCBE pair R8-3i-ATP8+4-3i-ATP8 (colored in red) or (**a**) variants containing one (grey) or two (black) $DddA^C$ point mutations from the following set: [K5A, R6A, G7A, T9A, V14A, P25A, T12K, V14K, N18K, P25K], (**b**) variants containing one or two $DddA^C$ point mutations from the following set: [K5A, R6A, G7A, T9A, V14A, P25A, T12K, V14K, N18K, P25K], in combination with either $DddA^N$ or $DddA^N_{C\Delta3}$, (**c**) variants containing one or two $DddA^C$ point mutations from the following set: [R6A, G7A, T9A, V14A, P25A, T12K, V14K, N18K, P25K], in combination with either $DddA^N$ and $DddA^C_{N\Delta5}$, or $DddA^N_{C\Delta3}$ and $DddA^C_{N\Delta5}$, (**d**) variants containing one, two or three changes in total, selected from any of the four approaches of single point mutations, truncations, electrostatic repulsion, and $dDddA^N$ capping. For **a**-**d**, values reflect the mean of *n*=3 independent biological replicates. The on-target editing efficiencies shown are for the most efficiently edited C•G within the spacing region. Source data are provided as a Source Data file.

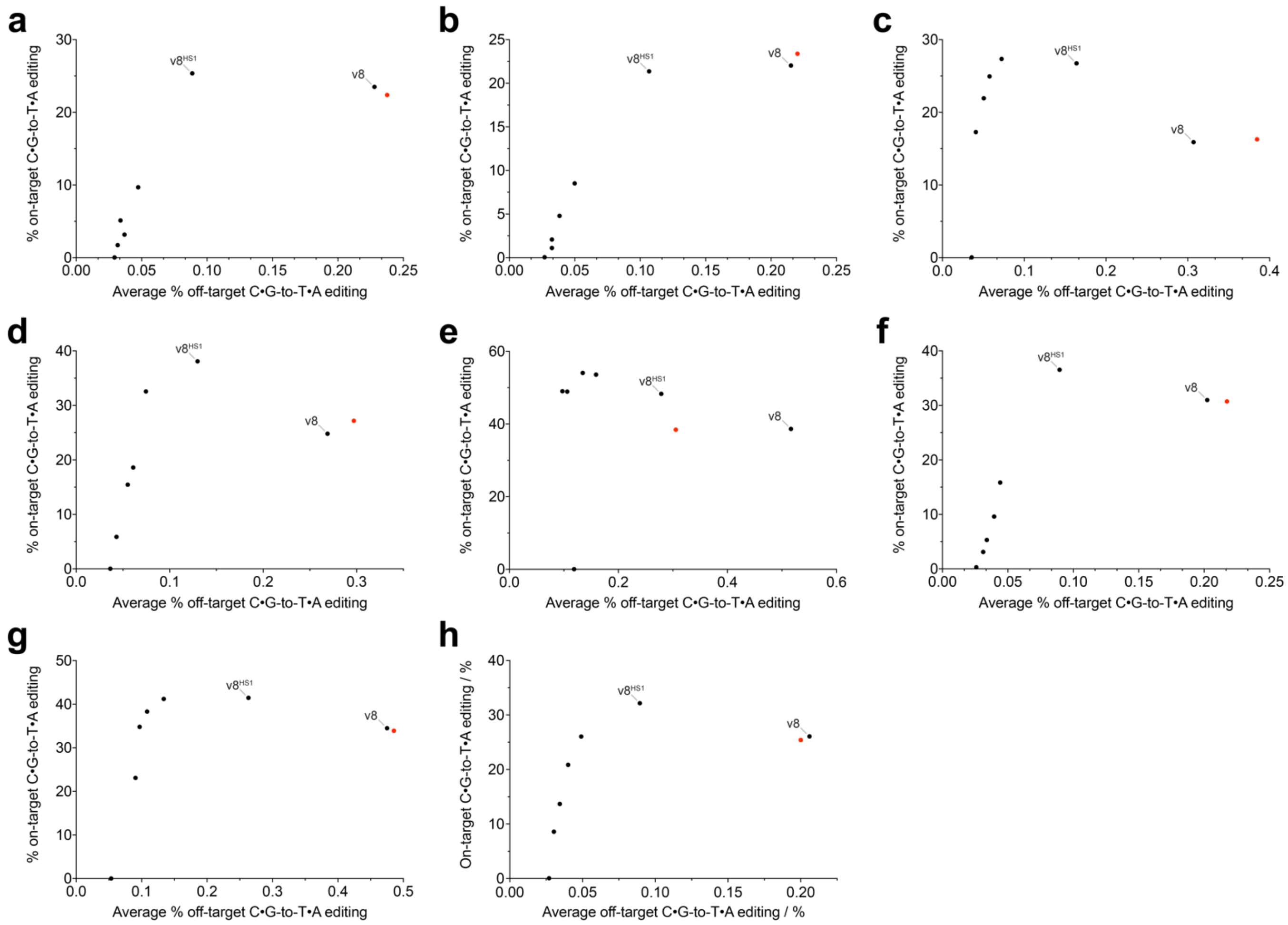

Supplementary Figure 22 | v8^{HS} ZF-DdCBE variants reduce off-target editing. a-h, On-target and average off-target editing efficiencies of HEK293T cells treated with v7 (colored in red), v8, or v8^{HS1} to v8^{HS5} ZF-DdCBE pairs (**a**) G24-R1b+G32-R1b, (**b**) G22-R13+G24-R13, (**c**) G32-R6a+G21-R6a, (**d**) G36-R6c+G212-R6c, (**e**) G33-V1+G35-V1, (**f**) G22-V2+G34-V2, (**g**) G33-V5+G36-V5, or (**h**) R8-3i-ATP8+10-3ii-ATP8. For **a-h**, values reflect the mean of *n*=3 independent biological replicates. The on-target editing efficiencies shown are for the most efficiently edited C•G within the spacing region. Source data are provided as a Source Data file.

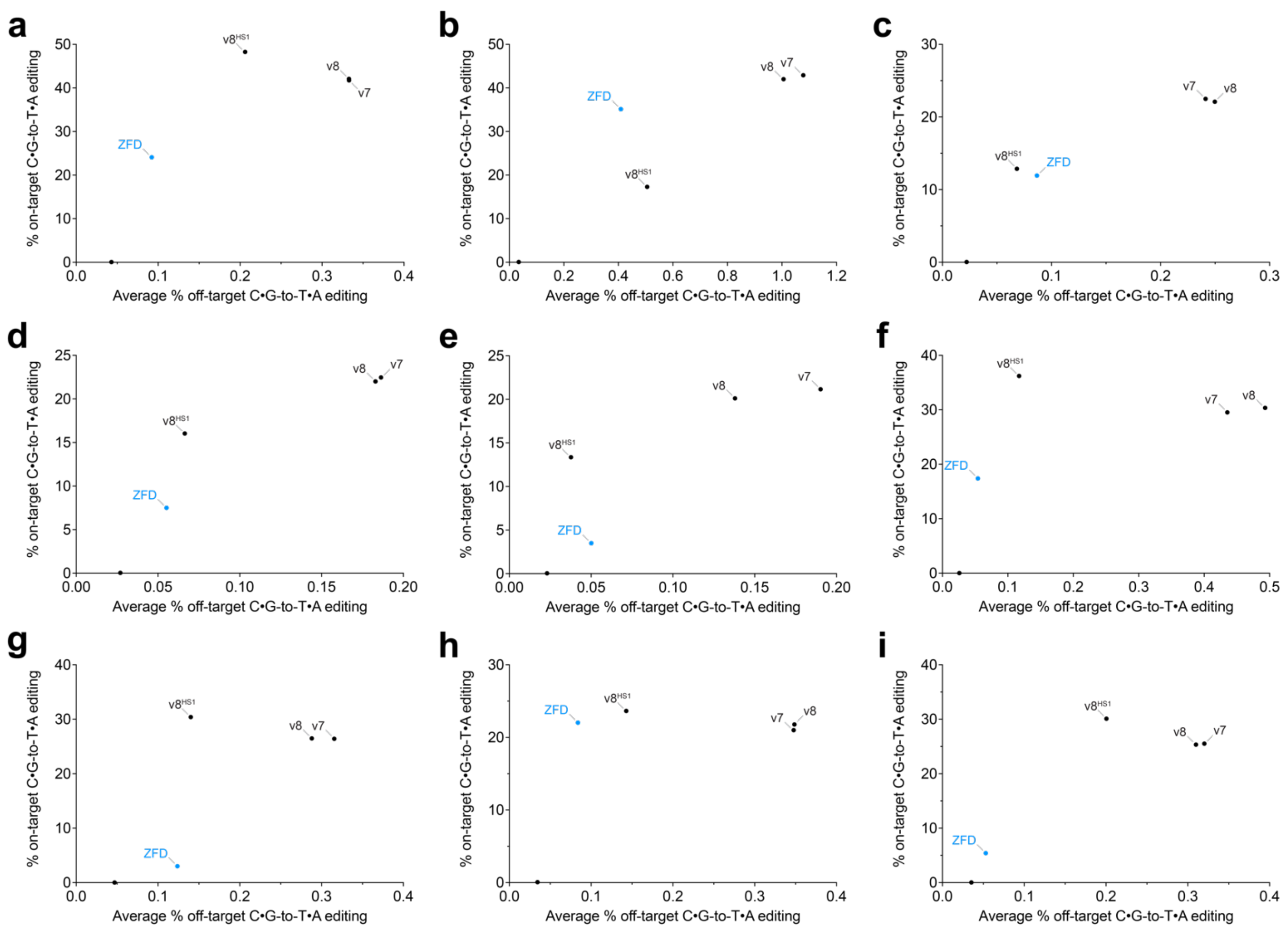

Supplementary Figure 23 | Comparison between v8^{HS1} ZF-DdCBEs and ZFDs. a-i, On-target and average off-target editing efficiencies of HEK293T cells treated with ZFDs (colored in blue), v7, v8, or v8^{HS1} ZF-DdCBE pairs (**a**) ND1-Left+ND1-Right, (**b**) ND2-Left+ND2-Right, (**c**) ND4L-Left+ND4L-Right, (**d**) ND4-Left+ND4-Right, (**e**) ND5-Left+ND5-Right, (**f**) ND52-Left+ND52-Right, (**g**) COX1-Left+COX1-Right, (**h**) COX2-Left+COX2-Right, or (**i**) CYB-Left+CYB-Right. For **a-g**, values reflect the mean of *n*=3 independent biological replicates. The on-target editing efficiencies shown are for the most efficiently edited C•G within the spacing region. Source data are provided as a Source Data file.

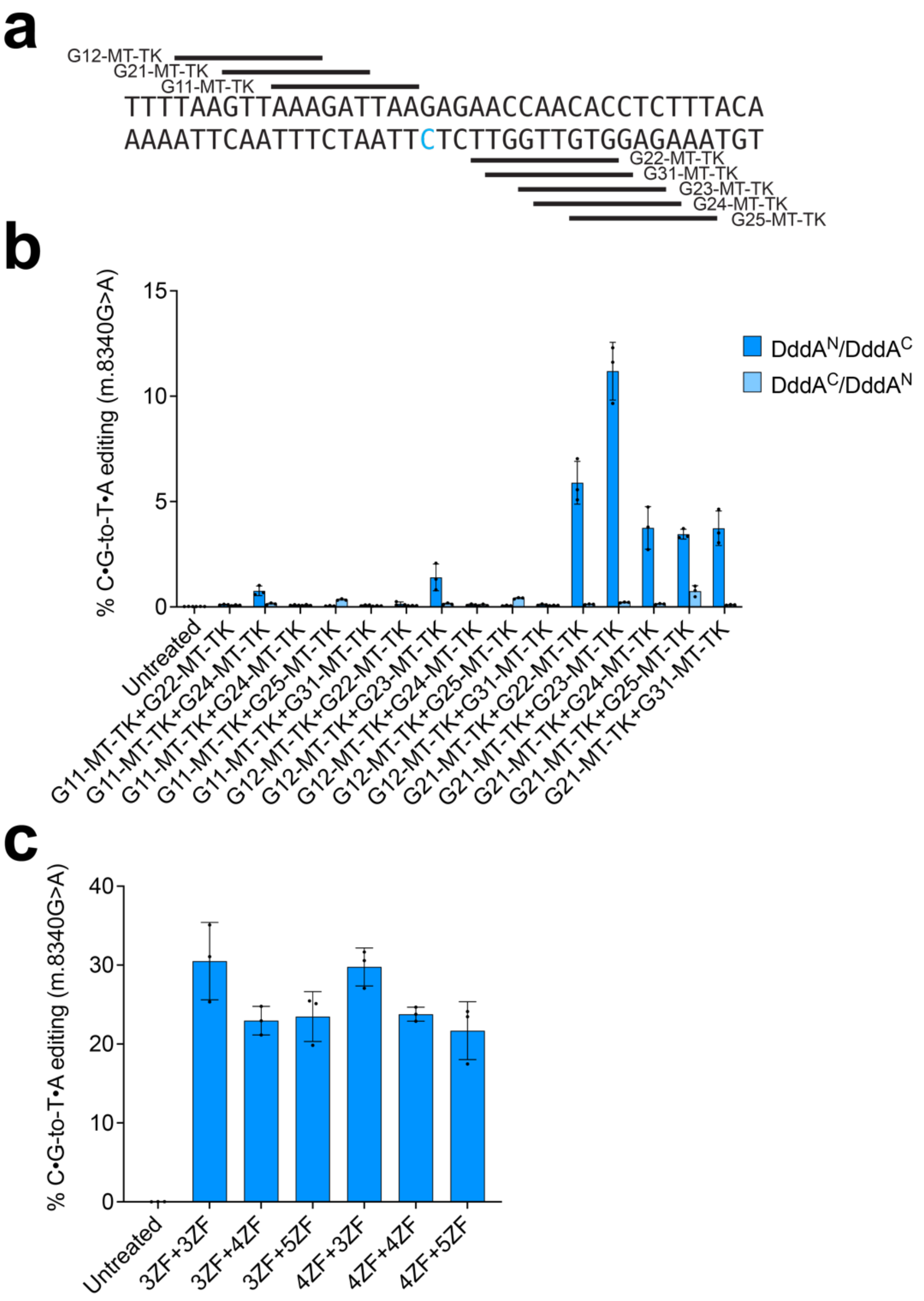

Supplementary Figure 24 | Optimized ZF-DdCBEs install m.8340G>A in HEK293T cells. a, Design of 3ZF arrays for ZF-DdCBE-mediated installation of m.8340G>A in human *MT-TK*. ZF-targeted DNA sequences are indicated by thick black lines vertically above or below the corresponding DNA sequence, and the target cytosine is colored blue. **b**, Mitochondrial DNA base editing efficiencies of HEK293T cells treated with v7 ZF-DdCBE pairs with the indicated split DddA orientation ($DddA^N/DddA^C$ signifies that the left ZF array is fused to $DddA^N$ and the right ZF array is fused to $DddA^C$). **c**, Mitochondrial DNA base editing efficiencies of HEK293T cells treated with 3ZF+3ZF $v7^{AGKS}$ ZF-DdCBE pair G21-MT-TK+G23-MT-TK or variants with the left and right ZF array extended to 4ZF or 5ZF as indicated. For **b** and **c**, values and errors reflect the mean±s.d. of *n*=3 independent biological replicates. The on-target editing efficiencies shown are for the most efficiently edited C•G within the spacing region. Source data are provided as a Source Data file.

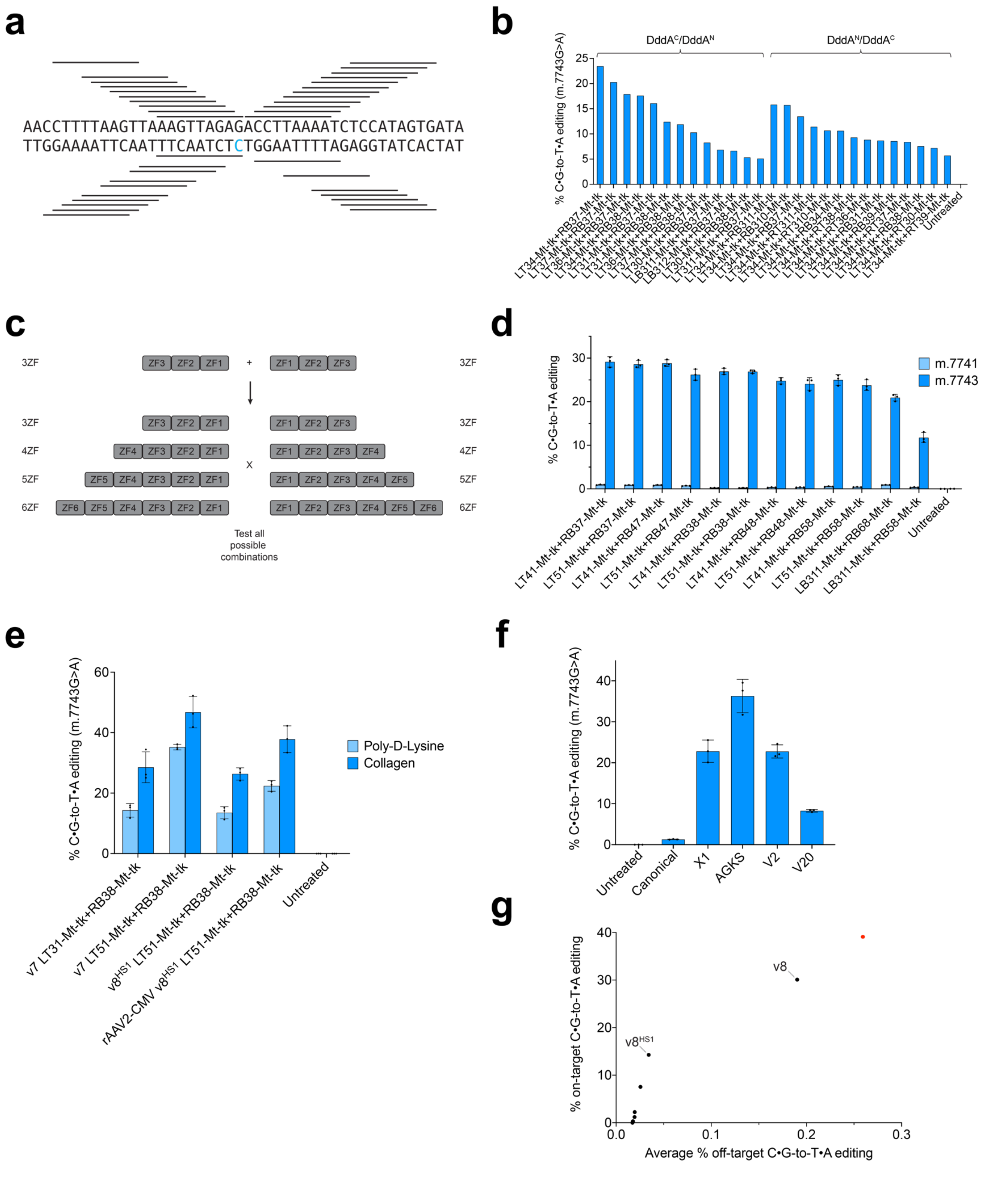

Supplementary Figure 25 | Optimized ZF-DdCBEs install m.7743G>A in C2C12 cells. a, 3ZF arrays for ZF-DdCBEs designed to install m.7743G>A in mouse *Mt-tk*. ZF-targeted DNA

sequences are indicated by thick black lines vertically above or below the corresponding DNA sequence, and the target cytosine is colored blue. **b, d** and **f**, Mitochondrial DNA base editing efficiencies of C2C12 cells treated with (**b**) the top 27 performing v7 ZF-DdCBE pairs from the initial 3ZF+3ZF panel designed to install m.7743G>A, (**d**) the top 12 performing extended v7 ZF-DdCBE pairs designed to install m.7743G>A, (**f**) the v7 ZF-DdCBE pair LT51-Mt-tk+RB38-Mt-tk with the indicated optimized ZF scaffolds. **c**, Extension of ZF arrays from 3ZF to 4ZF, 5ZF, or 6ZF (adding additional ZF repeats to the ZF arrays extending away from the spacing region in order to maintain a fixed deaminase positioning) to test the effects of ZF extension on ZF-DdCBE editing efficiency. **e**, Mitochondrial DNA base editing efficiencies of C2C12 cells plated on either poly-D-lysine- or collagen-coated plates treated with the indicated ZF-DdCBE pairs. **g**, On-target and average off-target editing efficiencies of C2C12 cells treated with v7 (colored in red), v8, or $v8^{HS1}$ to $v8^{HS5}$ ZF-DdCBE pair LT51-Mt-tk+ RB38-Mt-tk. For **d**-**f**, values and errors reflect the mean±s.d. of *n*=3 independent biological replicates. For **g**, values reflect the mean of *n*=3 independent biological replicates. The on-target editing efficiencies shown are for the most efficiently edited C•G within the spacing region. For **d-e**, all ZF-DdCBE pairs use the split DddA orientation $DddA^{C}/DddA^{N}$. Source data are provided as a Source Data file.

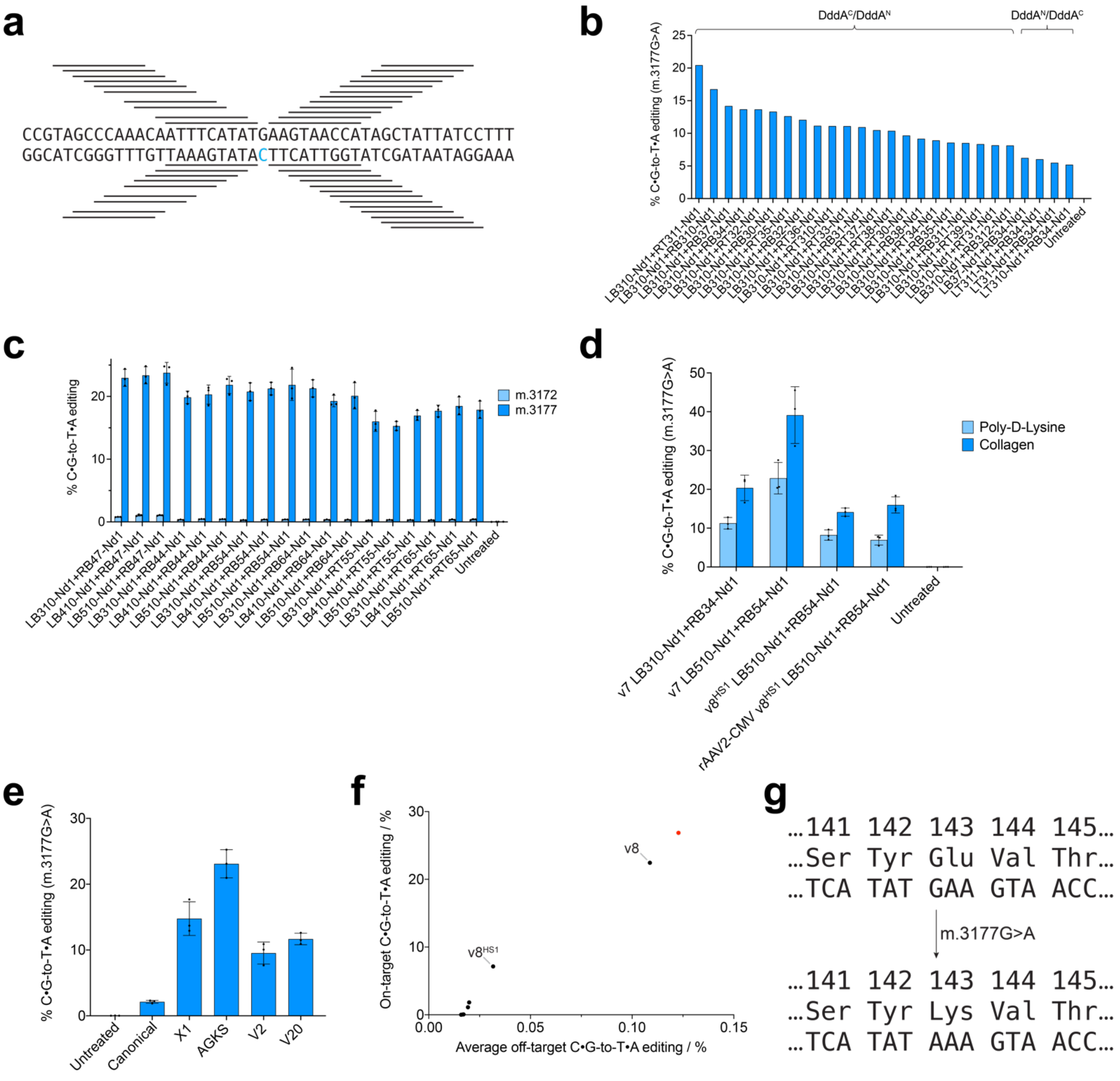

Supplementary Figure 26 | Optimized ZF-DdCBEs install m.3177G>A in C2C12 cells. a, 3ZF arrays for ZF-DdCBEs designed to install m.3177G>A in mouse *Nd1*. ZF-targeted DNA sequences are indicated by thick black lines vertically above or below the corresponding DNA sequence, and the target cytosine is colored blue. **b**, **c** and **e**, Mitochondrial DNA base editing efficiencies of C2C12 cells treated with (**b**) the top 26 performing v7 ZF-DdCBE pairs from the initial 3ZF+3ZF panel designed to install m.3177G>A, (**c**) the top 18 performing extended v7 ZF-DdCBE pairs designed to install m.3177G>A, (**e**) the v7 ZF-DdCBE pair LB510-Nd1+RB54-Nd1 with the indicated optimized ZF scaffolds. **d**, Mitochondrial DNA base editing efficiencies of C2C12 cells plated on either poly-D-lysine- or collagen-coated plates treated with the indicated ZF-DdCBE pairs. **f**, On-target and average off-target editing efficiencies of C2C12 cells treated

with v7 (colored in red), v8, or v8^{HS1} to v8^{HS5} ZF-DdCBE pair LB510-Nd1+RB54-Nd1. **g**, The m.3177G>A mutation in mouse *Nd1* creates a missense E143K mutation. For **b**-**e**, values and errors reflect the mean±s.d. of *n*=3 independent biological replicates. For **f**, values reflect the mean of *n*=3 independent biological replicates. The on-target editing efficiencies shown are for the most efficiently edited C•G within the spacing region. For **c-d**, all ZF-DdCBE pairs use the split DddA orientation DddA^{C}/DddA^{N}. Source data are provided as a Source Data file.

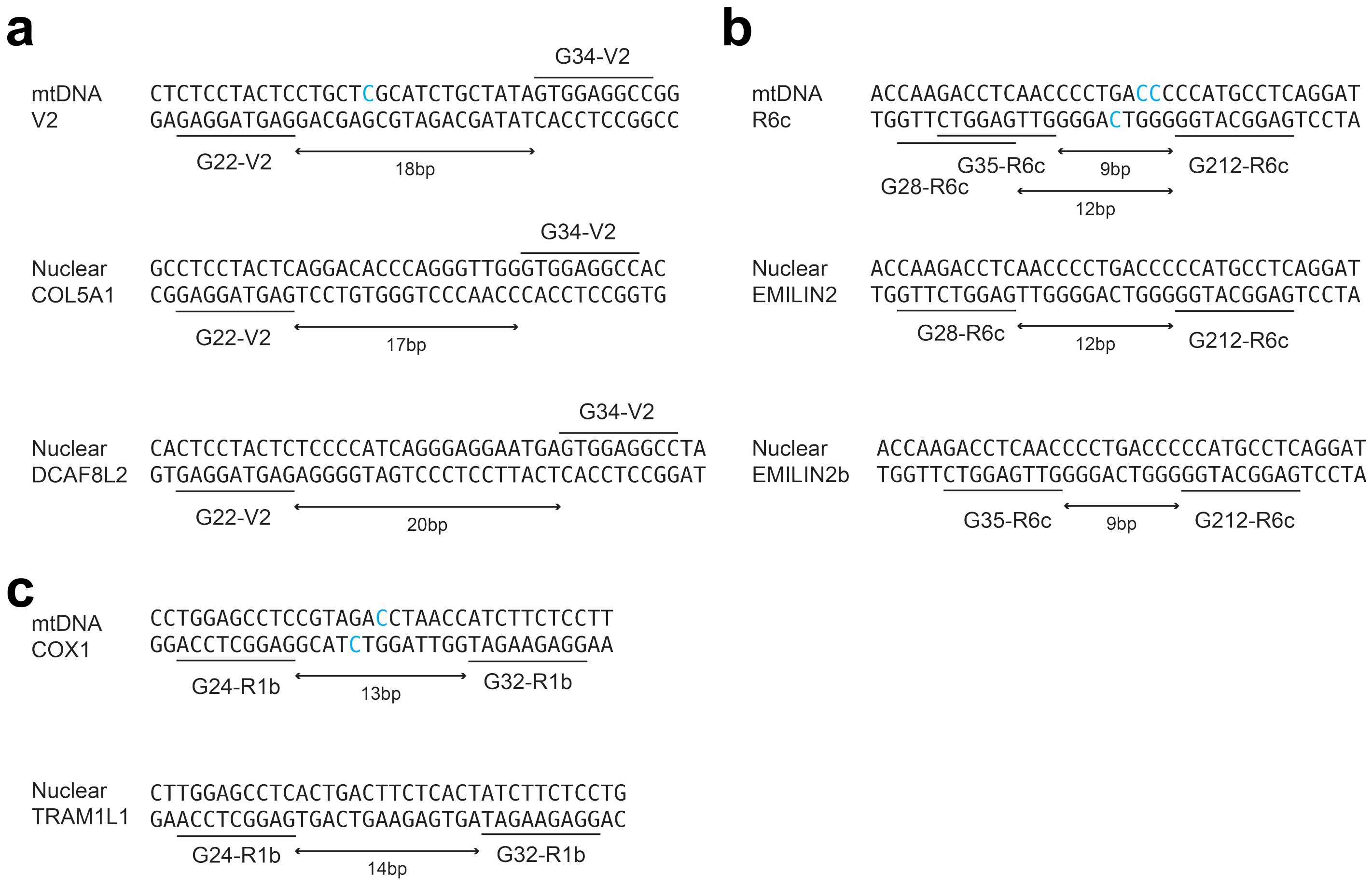

Supplementary Figure 27 | Converting mitochondrial ZF-DdCBEs into nuclear ZF-DdCBEs. a-c, 3ZF arrays for ZF-DdCBEs designed to edit mitochondrial sites, or nuclear sites with high sequence similarity. ZF-targeted DNA sequences are indicated by thick black lines vertically above or below the corresponding DNA sequence, spacing regions are marked with arrows, and the target cytosine(s) edited in mitochondrial DNA with high efficiency are colored blue.

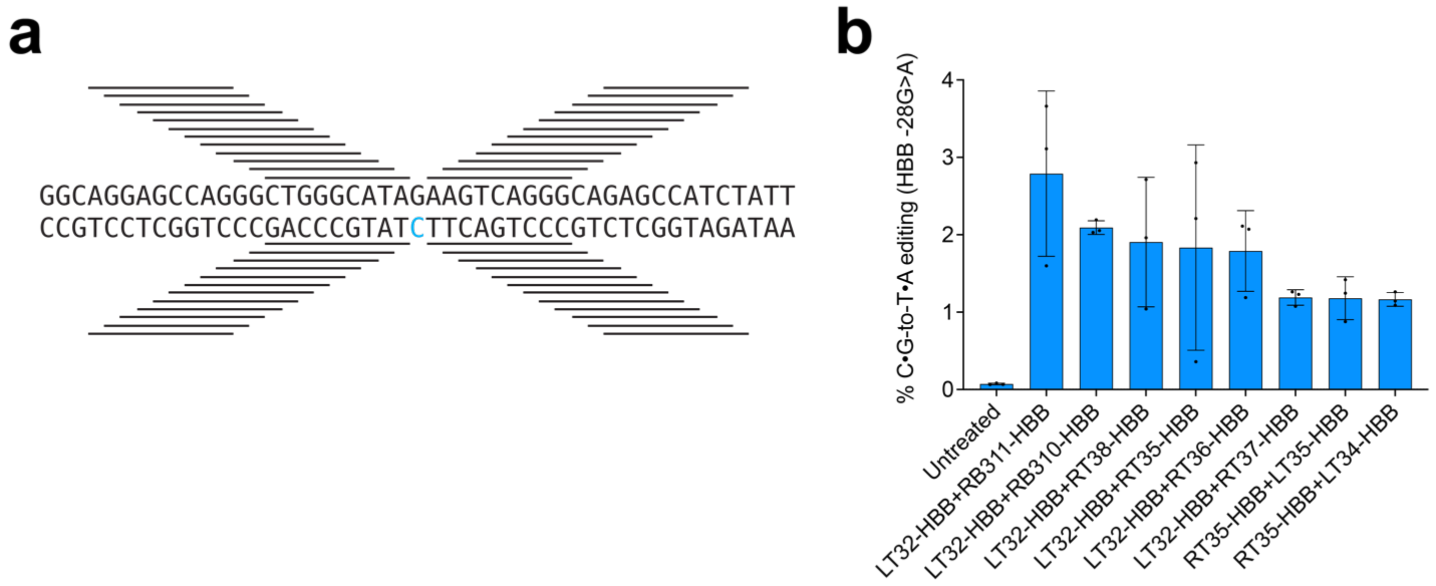

Supplementary Figure 28 | Correction of a nuclear disease-causing mutation using ZF-DdCBEs. a, 3ZF arrays for ZF-DdCBEs designed to correct human *HBB* -28(A>G). ZF-targeted DNA sequences are indicated by thick black lines vertically above or below the corresponding DNA sequence, and the target cytosine is colored blue. **b,** Mitochondrial DNA base editing efficiencies of HEK293T-HBB cells nuclear ZF-DdCBE pairs designed to correct *HBB* -28(A>G). All ZF-DdCBE pairs use the split DddA orientation $DddA^N/DddA^C$. For **b**, values and errors reflect the mean±s.d. of *n*=3 independent biological replicates. Source data are provided as a Source Data file.

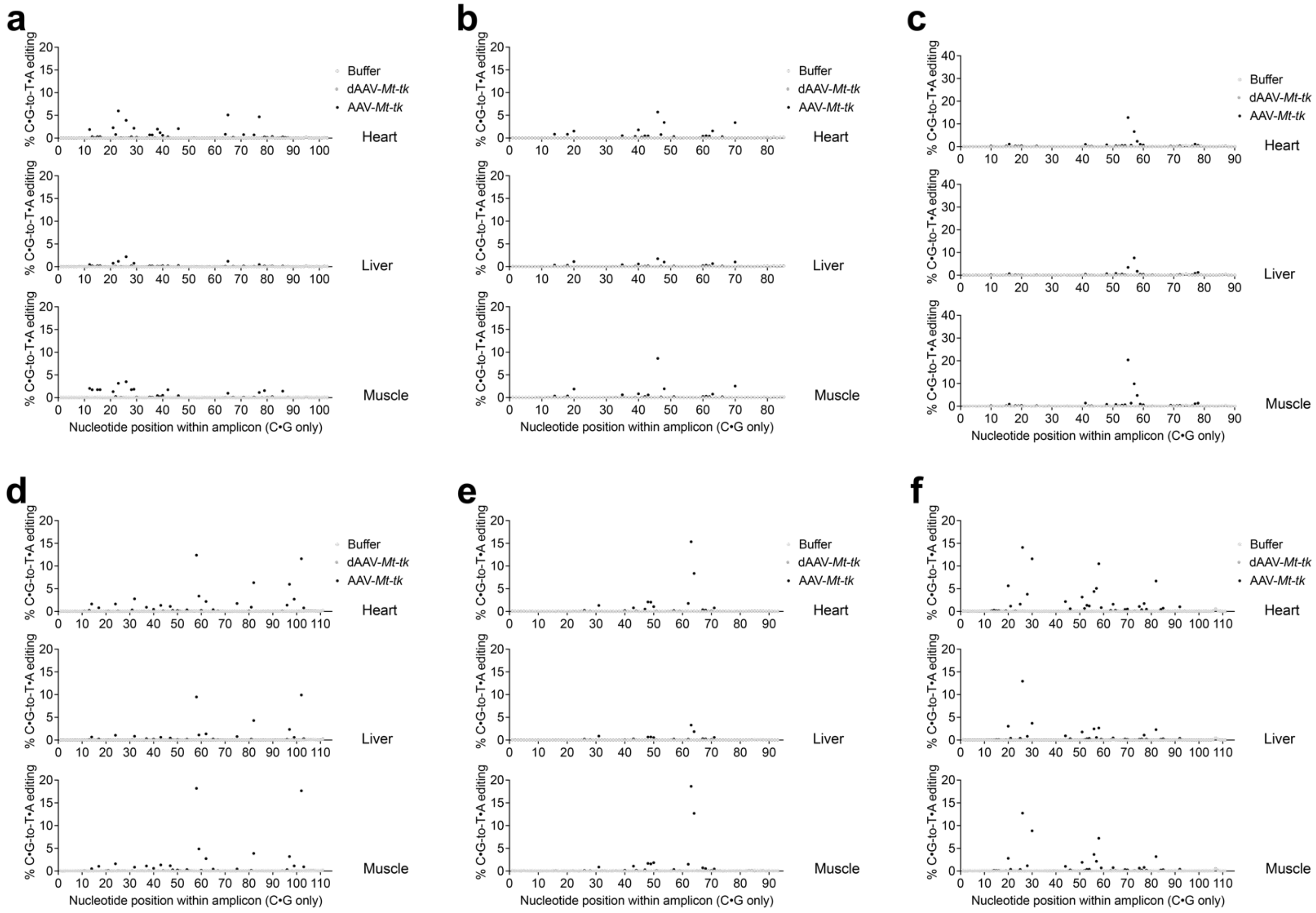

Supplementary Figure 29 | Off-target editing analysis of mice treated with AAV-*Mt-tk*. a-f, Off-target editing efficiencies within mitochondrial off-target amplicon (**a**) OT1, (**b**) OT3, (**c**) OT4, (**d**) OT10, (**e**) OT11, or (**f**) OT12 of tissue samples from mice treated with buffer, dAAV-*Mt-tk* or AAV-*Mt-tk*. Values reflect the mean of *n*=4, 4 and 7 for mice treated with buffer, AAV-*Mt-tk*, or dAAV-*Mt-tk*, respectively. Source data are provided as a Source Data file.

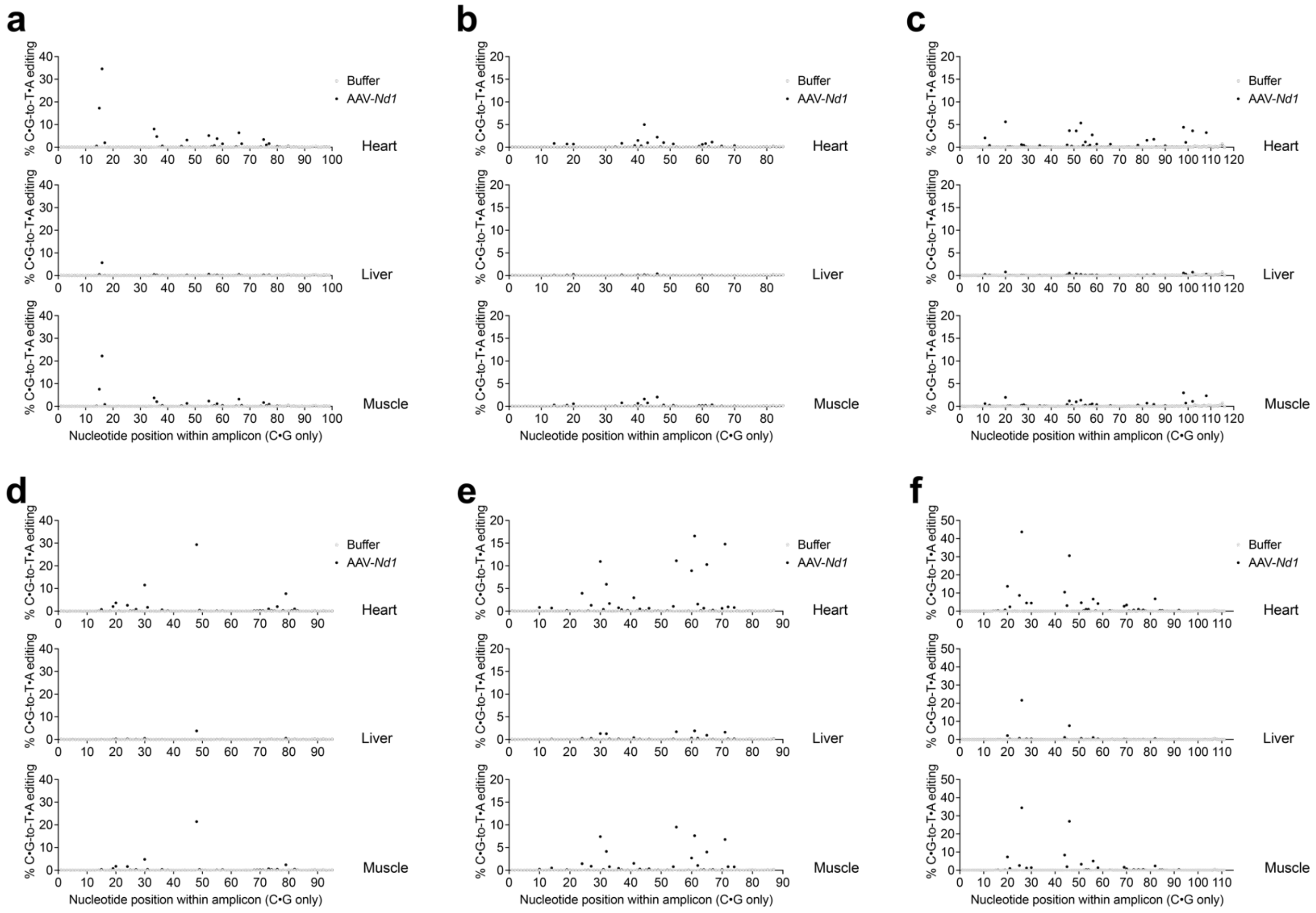

Supplementary Figure 30 | Off-target editing analysis of mice treated with AAV-*Nd1*. a-f, Off-target editing efficiencies within mitochondrial off-target amplicon (**a**) OT2, (**b**) OT3, (**c**) OT5, (**d**) OT6, (**e**) OT9, or (**f**) OT12 of tissue samples from mice treated with buffer or AAV-*Nd1*. Values reflect the mean of *n*=4 and 7 for mice treated with buffer or AAV-*Nd1*, respectively. Source data are provided as a Source Data file.

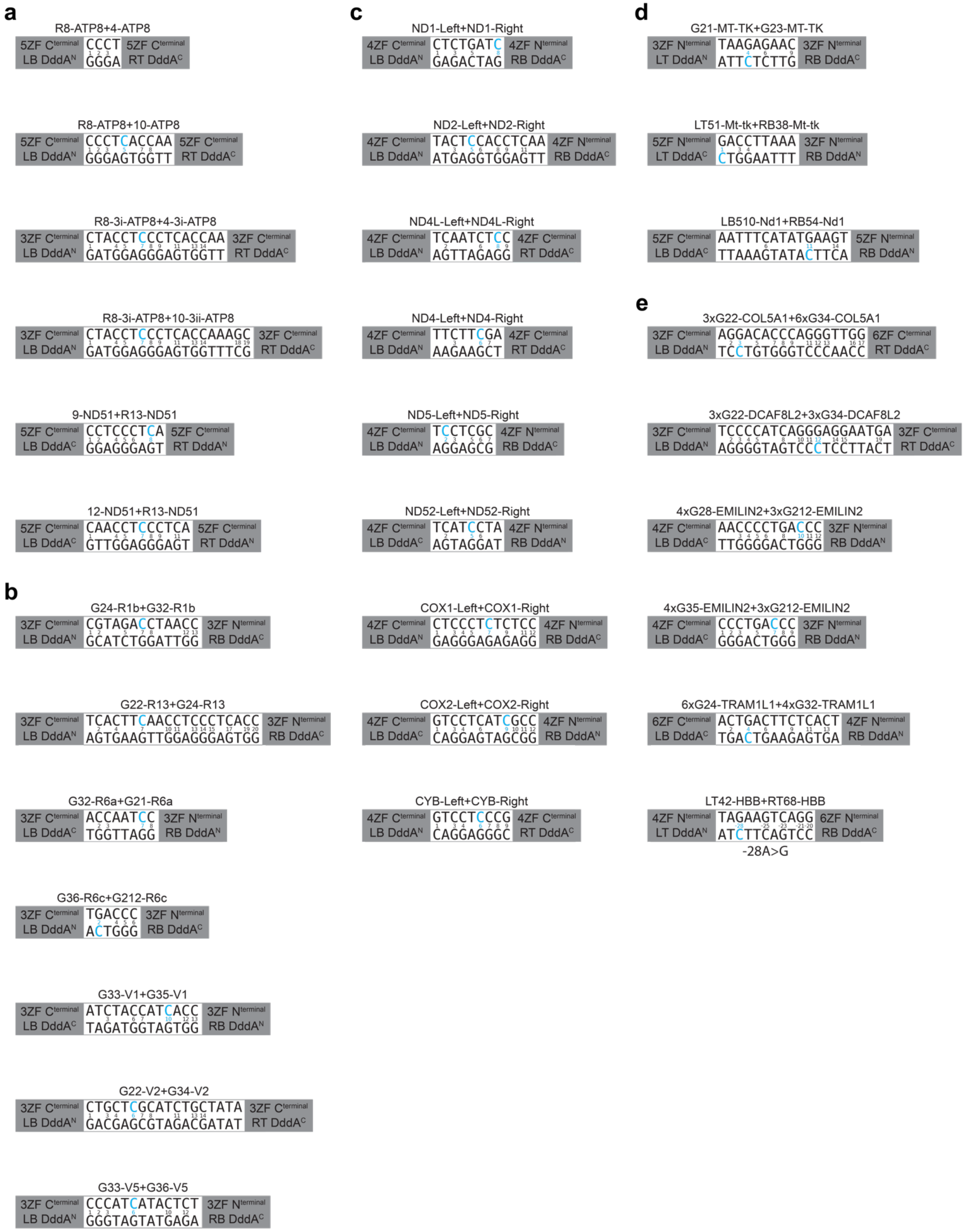

Supplementary Figure 31 | Configurations and DNA sequences of spacing regions for ZF-DdCBE pairs used in this study. a, Initial mitochondrial ZF-DdCBE pairs used to establish v1

to v8 architectural improvements. **b**, Additional mitochondrial ZF-DdCBE pairs used to validate optimized architectures and HS variants. **c**, ZFD-derived mitochondrial ZF-DdCBE pairs. **d**, Nuclear ZF-DdCBE pairs. For each site the DNA spacing region, split DddA orientation, ZF array lengths, architecture type (N- or C-terminal fusion of split DddA relative to the ZF array), and ZF-targeted DNA strands (LT, LB, RT, RB=left top, left bottom, right top, right bottom, respectively) are shown, and the cytosine with the highest editing efficiency is colored in blue. ZF-DdCBE naming convention follows A+B where A and B specify the left and right ZF, respectively. Nucleotide numbering starts with the first 5'-nucleotide in the spacing region designated position 1. For R8-ATP8+4-ATP8, nucleotide C5 has the highest editing efficiency.

Supplementary Note 1 | Defining new ZF scaffold AGKS

We noticed sequence similarity between the beta-motifs in *ZFN268*(F1) and Sp1C, YACPVESCDRRFS and YKCPECGKSFSQK respectively, which are also commonly used in designed ZF arrays[1]. These sequences differ by the insertion of two residues in addition to four substitutions. We defined a set of nine beta-motifs in which the sequences were progressively mutated to incrementally revert the *ZFN268*(F1) beta-motif towards the Sp1C beta-motif and vice versa (Fig. S9a). We constructed v5 ZF-DdCBE variants based on the X1 scaffold in which we changed only the beta-motif and tested two ZF-DdCBE pairs to determine if any of these new ZF scaffold sequences could improve editing efficiency. Compared to the canonical *ZNF268*-derived scaffold, we found that scaffold AGKS conferred an increase in editing efficiency of 1.7-fold across the two pairs tested (Fig. S9b,c). We included scaffold AGKS in our set of optimized ZF scaffolds.

Supplementary Note 2 | Shifting the position of the canonical G1397 split site within DddA

As an alternative to truncating $DddA^N$ and $DddA^C$ to reduce ZF-DdCBE off-target editing, we investigated the effects of shifting the position of the canonical G1397 split site within DddA to create split DddA halves with a longer $DddA^N$ and a shorter $DddA^C$. We tested a series of ZF-DdCBE pairs in which $DddA^N$ was incrementally extended at its C-terminus by between one and 15 residues, designated $\mathrm{DddA}^{\mathrm{N}}_{\mathrm{C+1}}$ to $\mathrm{DddA}^{\mathrm{N}}_{\mathrm{C+15}}$, while at the same time $DddA^C$ was incrementally truncated at its C-terminus by between one and 15 residues, designated $\mathrm{DddA}^{\mathrm{C}}_{\mathrm{N\Delta1-15}}$ (Fig. S17a). The best combination ($\mathrm{DddA}^{\mathrm{N}}_{\mathrm{C+5}}$ with $\mathrm{DddA}^{\mathrm{C}}_{\mathrm{N\Delta5}}$) exhibited a 1.2-fold reduction in off-target editing while retaining 97% of on-target editing relative to the canonical ZF-DdCBE pair. These results suggest that shifting the position of the split site can alter the ratio of on-target to off-target editing performance of ZF-DdCBEs, but this approach does not yield ZF-DdCBEs with a specificity profile better than can be achieved by truncation. We confirmed that the split halves $\mathrm{DddA}^{\mathrm{N}}_{\mathrm{C+1}}$ to $\mathrm{DddA}^{\mathrm{N}}_{\mathrm{C+14}}$ remained inactive by themselves by transfecting only a single ZF-DdCBE half carrying a $DddA^N$ variant, and observing no detectable base editing in the absence of a $DddA^C$ variant (Fig. S17b). We note that $\mathrm{DddA}^{\mathrm{N}}_{\mathrm{C+15}}$ displayed base editing activity, signifying that C-terminal truncations of DddA of greater than 16 amino acids are required to abolish DddA deaminase activity.

Supplementary Note 3 | Combining approaches to reduce ZF-DdCBE off-target editing

Having established four different approaches to reduce ZF-DdCBE off-target editing, we investigated whether these approaches could be combined additively to create variants with even better specificity profiles. To test the effects of combining point mutations, we selected a set of 10 single point mutations (K5A, R6A, G7A, T9A, V14A, P25A, T12K, V14K, N18K, P25K) and tested all 43 pairwise combinations of double mutants (Fig. S21a). To test the effects of combining point mutations and truncations, we selected a set of eight single point mutations (G7A, T9A, V14A, P25A, T12K, V14K, N18K, P25K) and tested 123 different ZF-DdCBE variants comprising all possible single or double point mutations either alone or in combination with the truncations $\mathrm{DddA}^{\mathrm{N}}_{\mathrm{C\Delta3}}$, $\mathrm{DddA}^{\mathrm{C}}_{\mathrm{N\Delta5}}$, or both (Fig. S21b,c). To investigate the effects of combining any of the approaches of single point mutations, truncations, electrostatic repulsion, and dDddA^{N} capping, we also tested combinations comprising one variant from any one, two or three of these four approaches (Fig. S21d). Collectively, these results revealed that combining more than one mutation or more than one approach not only leads to a greater reduction in off-target editing compared to using a single mutation or approach, but also a greater reduction in on-target editing. We concluded from these experiments that each of these four approaches is able to create ZF-DdCBEs with improved specificity profiles, but it is not apparent which approaches synergize better than any individual strategies. We decided that the best combinations of these strategies to minimize off-target ZF-DdCBE editing should be empirically determined.

Supplementary Note 4 | Optimizing ZF-DdCBEs to install m.7743G>A within *Mt-tk*

We initially identified 27 ZF-DdCBE pairs able to install the desired edit in mouse C2C12 cells with efficiencies ranging from 5% to 23% (Fig. S25b). To assess whether ZF extension could improve editing performance, for these 27 pairs we extended each 3ZF to 4ZF, 5ZF, or 6ZF where possible and tested the resulting ZF-DdCBE combinations (Fig. S25c). We added additional ZF repeats to the ZF arrays extending away from the spacing region in order to maintain a fixed deaminase positioning. From our 12 best-performing ZF-DdCBE combinations we selected a pair (LT51-Mt-tk+ RB38-Mt-tk) that showed a good balance between high on-target activity and low bystander or off-target editing (Fig. S25d). This final 3ZF+5ZF v7^{AGKS} ZF-DdCBE pair exhibited a 2.5-fold improvement relative to its corresponding 3ZF+3ZF pair, installing the m.7743G>A mutation at an efficiency of 35% and with excellent specificity (Fig. S25e). We tested alternative ZF scaffolds and confirmed that v7^{AGKS} architecture supported the highest on-target editing efficiency for this ZF-DdCBE pair (Fig. S25f). We also identified that editing efficiency could be increased to 47% by plating C2C12 cells on collagen-coated plates instead of poly-D-lysine-coated plates (Fig. S25e).

Supplementary Note 5 | Optimizing ZF-DdCBEs to install m.3177G>A within *Nd1*

We identified 26 ZF-DdCBE pairs able to install the desired edit with efficiencies ranging from 5% to 20% (Fig. S26b). To assess whether ZF extension could improve editing performance, for 34 pairs we extended each 3ZF to 4ZF, 5ZF, or 6ZF where possible and tested the resulting ZF-DdCBE combinations (Fig. S25c). From our 18 best-performing ZF-DdCBE combinations we selected a pair (LB510-Nd1/RB54-Nd1) that showed a good balance between high on-target activity and low bystander or off-target editing (Fig. S26c). This final 5ZF+5ZF $v7^{AGKS}$ ZF-DdCBE pair exhibited a 2.0-fold improvement relative to the unoptimized 3ZF+3ZF pair, installing the m.3177G>A mutation at an efficiency of 23% and with excellent specificity (Fig. S26d). We tested alternative ZF scaffolds and confirmed that $v7^{AGKS}$ architecture supported the highest on-target editing efficiency for this ZF-DdCBE pair (Fig. S26e). We also identified that editing efficiency could be increased to 39% by plating C2C12 cells on collagen-coated plates instead of poly-D-lysine-coated plates (Fig. S26d).

Supplementary Note 6 | Prevalence of GNN-binding modules in ZF arrays within active ZF-DdCBE pairs

To investigate the whether the number of GNN-binding modules in ZF arrays correlated with ZF-DdCBE performance, we analyzed data from the experiments in which we screened ZF-DdCBE pairs for the installation of m.7743G>A in *Mt-tk,* and m.3177G>A in *Nd1*. Because these datasets involve large numbers of ZF-DdCBE pairs tested at the same sites, we considered these data the most appropriate for investigating any such trend.

For the installation of m.7743G>A in *Mt-tk*, we tested all possible ZF-DdCBE combinations arising from a set of 39 individual 3ZF arrays (Fig. S25a), containing an average of 0.31 GNN-targeting repeats per 3ZF array. The 27 top-performing ZF-DdCBE pairs comprised a total of 22 unique 3ZF arrays (Fig. S25b), containing an average of 0.55 GNN-targeting repeats. The top ten ZF-DdCBE pairs comprised eight unique 3ZF arrays, with an average of 1.13 GNN-targeting repeats. The top five ZF-DdCBE pairs comprised six unique 3ZF arrays, with an average of 1.17 GNN-targeting repeats.

For the installation of m.3177G>A in *Nd1*, we tested all possible ZF-DdCBE combinations arising from a set of 44 individual 3ZF arrays (Fig. S26a), containing an average of 0.48 GNN-targeting repeats per 3ZF array. The 26 top-performing ZF-DdCBE pairs comprised a total of 27 unique 3ZF arrays (Fig. S26b), containing an average of 0.59 GNN-targeting repeats. The top ten ZF-DdCBE pairs comprised 11 unique 3ZF arrays, with an average of 0.91 GNN-targeting repeats. The top five ZF-DdCBE pairs comprised six unique 3ZF arrays, with an average of 1.33 GNN-targeting repeats.

This analysis suggests that there is some level of enrichment for GNN-targeting repeats within more highly active ZF-DdCBE pairs, presumably due to higher binding affinity and specificity. Drawing conclusions from any single site is strongly dependent on the particular sequence context of the site tested, and therefore we believe that a more expansive and systematic investigation would support more generalizable conclusions. That said, ZF arrays designed with a higher proportion of GNN-binding modules on average may lead to ZF-DdCBEs with higher performance.

Supplementary Reference

1 Beerli, R. R., Segal, D. J., Dreier, B. & Barbas, C. F., 3rd. Toward controlling gene expression at will: specific regulation of the erbB-2/HER-2 promoter by using polydactyl zinc finger proteins constructed from modular building blocks. *Proc Natl Acad Sci U S A* **95**, 14628-14633, doi:10.1073/pnas.95.25.14628 (1998).
