# Supplementary material for: Compact zinc finger base editors that edit mitochondrial or nuclear DNA in vitro and in vivo
Source: Nat Commun. 2022 Nov 23;13:7204. doi: 10.1038/s41467-022-34784-7 (PMC9684478; doi:10.1038/s41467-022-34784-7)
Supplement: Supplementary file 2 — Reporting Summary [file 41467_2022_34784_MOESM2_ESM.pdf]

nature portfolio

Corresponding author(s): David R. Liu

Last updated by author(s): Oct 29, 2022

Reporting Summary

Nature Portfolio wishes to improve the reproducibility of the work that we publish. This form provides structure for consistency and transparency in reporting. For further information on Nature Portfolio policies, see our Editorial Policies and the Editorial Policy Checklist.

Statistics

For all statistical analyses, confirm that the following items are present in the figure legend, table legend, main text, or Methods section.

n/a	Confirmed	
☐	☒	The exact sample size (*n*) for each experimental group/condition, given as a discrete number and unit of measurement
☐	☒	A statement on whether measurements were taken from distinct samples or whether the same sample was measured repeatedly
☒	☐	The statistical test(s) used AND whether they are one- or two-sided<br>*Only common tests should be described solely by name; describe more complex techniques in the Methods section.*
☒	☐	A description of all covariates tested
☒	☐	A description of any assumptions or corrections, such as tests of normality and adjustment for multiple comparisons
☐	☒	A full description of the statistical parameters including central tendency (e.g. means) or other basic estimates (e.g. regression coefficient) AND variation (e.g. standard deviation) or associated estimates of uncertainty (e.g. confidence intervals)
☒	☐	For null hypothesis testing, the test statistic (e.g. *F*, *t*, *r*) with confidence intervals, effect sizes, degrees of freedom and *P* value noted<br>*Give P values as exact values whenever suitable.*
☒	☐	For Bayesian analysis, information on the choice of priors and Markov chain Monte Carlo settings
☒	☐	For hierarchical and complex designs, identification of the appropriate level for tests and full reporting of outcomes
☒	☐	Estimates of effect sizes (e.g. Cohen's *d*, Pearson's *r*), indicating how they were calculated

Our web collection on statistics for biologists contains articles on many of the points above.

Software and code

Policy information about availability of computer code

Data collection	High-throughput DNA sequencing data was collected on Illumina MiSeq Control software (3.1) and instruments. CRISPResso output files were imported into Microsoft Excel (version 16.61.1) for analyses.
Data analysis	Sequencing reads were demultiplexed using MiSeq Reporter (Illumina) and analyzed using Crispresso2 (version 2.1.3). Prism 8 (GraphPad) was used to generate dot plots and bar plots of these data. Further details and references are provided in the Methods.

For manuscripts utilizing custom algorithms or software that are central to the research but not yet described in published literature, software must be made available to editors and reviewers. We strongly encourage code deposition in a community repository (e.g. GitHub). See the Nature Portfolio guidelines for submitting code & software for further information.

Data

Policy information about availability of data

All manuscripts must include a data availability statement. This statement should provide the following information, where applicable:

- Accession codes, unique identifiers, or web links for publicly available datasets
- A description of any restrictions on data availability
- For clinical datasets or third party data, please ensure that the statement adheres to our policy

High-throughput DNA sequencing FASTQ files will be available from the NCBI SRA under BioProject PRJNA835113. Plasmids encoding select ZF-DdCBEs will be made available at Addgene for distribution. Amino acid sequences of all ZF-DdCBEs in this study are provided in Supplementary Data 1. Data from all ZF-DdCBE specificity optimization experiments are provided in Supplementary Data 2.

Human research participants

Policy information about studies involving human research participants and Sex and Gender in Research.

Reporting on sex and gender	N/A
Population characteristics	N/A
Recruitment	N/A
Ethics oversight	N/A

Note that full information on the approval of the study protocol must also be provided in the manuscript.

Field-specific reporting

Please select the one below that is the best fit for your research. If you are not sure, read the appropriate sections before making your selection.

[x] Life sciences [ ] Behavioural & social sciences [ ] Ecological, evolutionary & environmental sciences

For a reference copy of the document with all sections, see nature.com/documents/nr-reporting-summary-flat.pdf

Life sciences study design

All studies must disclose on these points even when the disclosure is negative.

Sample size	Sample sizes were n=3 independent biological replicates. No statistical methods were used to predetermine sample size. We chose this sample size based on existing procedures and standards in the field, and because have found it to be sufficient in mammalian cell gene editing experiments to yield reproducible mean values.
Data exclusions	No data was excluded.
Replication	Biological triplicate experiments were done with distinct aliquots of cells at intervals ranging from days to months between experiments. Findings have been replicated and the base editors developed in this work have been independently tested by several researchers.
Randomization	All independent biological replicates were treated identically. Thus randomization was not relevant to this study.
Blinding	Mammalian cells used in this study were treated under identical conditions; blinding was not used.

Reporting for specific materials, systems and methods

We require information from authors about some types of materials, experimental systems and methods used in many studies. Here, indicate whether each material, system or method listed is relevant to your study. If you are not sure if a list item applies to your research, read the appropriate section before selecting a response.

Materials & experimental systems

n/a	Involved in the study
☒	☐ Antibodies
☐	☒ Eukaryotic cell lines
☒	☐ Palaeontology and archaeology
☐	☒ Animals and other organisms
☒	☐ Clinical data
☒	☐ Dual use research of concern

Methods

n/a	Involved in the study
☒	☐ ChIP-seq
☒	☐ Flow cytometry
☒	☐ MRI-based neuroimaging

Eukaryotic cell lines

Policy information about cell lines and Sex and Gender in Research

Cell line source(s)	HEK293T (CRL-3216) and C2C12 (CRL-1772) cells were purchased from American Type Culture Collection (ATCC).
Authentication	Cells were authenticated by the supplier using STR analysis.
Mycoplasma contamination	HEK293T cell lines tested negative for mycoplasma. C2C12 cell lines were not tested for mycoplasma contamination.
Commonly misidentified lines (See ICLAC register)	None used.

Animals and other research organisms

Policy information about studies involving animals; ARRIVE guidelines recommended for reporting animal research, and Sex and Gender in Research

Laboratory animals	Mice (Mus musculus) in a C57BL/6J background were obtained from Charles River Laboratories. The animals were maintained in a temperature- and humidity-controlled animal care facility with a 12 hour light/12 hour dark cycle and free access to water and food and sacrificed by cervical dislocation. Newborn mice were injected at postnatal day 1.
Wild animals	The study did not involve wild animals.
Reporting on sex	Mice cohorts were n=4 or 7 animals. Sex was not considered in study design due to insufficient sample size to draw conclusions based on sex, which reflected the natural litter composition.
Field-collected samples	The study did not involve samples collected from the field.
Ethics oversight	All animal experiments were carried out in accordance with the UK Animals (Scientific Procedures) Act 1986 (Procedure Project Licence: P6C20975A) and EU Directive 2010/63/EU and authorized by the University of Cambridge Animal Welfare and Ethical Review Body.

Note that full information on the approval of the study protocol must also be provided in the manuscript.
