# Supplementary material for: Compact zinc finger base editors that edit mitochondrial or nuclear DNA in vitro and in vivo
Source: Nat Commun. 2022 Nov 23;13:7204. doi: 10.1038/s41467-022-34784-7 (PMC9684478; doi:10.1038/s41467-022-34784-7)
Supplement: Supplementary file 6 — Description of Additional Supplementary Files [file 41467_2022_34784_MOESM6_ESM.pdf]

Title: Supplementary Data 1
Description: Sequences and primers used in this study

Title: Supplementary Data 2
Description: Experimental data for strategies to reduce ZF-DdCBE off-target editing

Title: Supplementary Data 3
Description: ZF design and cloning guide
